# The Use of Social Media to Increase the Impact of Health Research: Systematic Review

**DOI:** 10.2196/15607

**Published:** 2020-07-06

**Authors:** Marco Bardus, Rola El Rassi, Mohamad Chahrour, Elie W Akl, Abdul Sattar Raslan, Lokman I Meho, Elie A Akl

**Affiliations:** 1 Department of Health Promotion and Community Health Faculty of Health Sciences American University of Beirut Beirut Lebanon; 2 Clinical Research Institute American University of Beirut Beirut Lebanon; 3 Department of Pediatric and Adolescent Medicine American University of Beirut Medical Center Beirut Lebanon; 4 University Libraries American University of Beirut Beirut Lebanon; 5 Department of Internal Medicine American University of Beirut Medical Center Beirut Lebanon

**Keywords:** social media, research, bibliometrics, Altmetrics, journal impact factor, translational medical research

## Abstract

**Background:**

Academics in all disciplines increasingly use social media to share their publications on the internet, reaching out to different audiences. In the last few years, specific indicators of social media impact have been developed (eg, Altmetrics), to complement traditional bibliometric indicators (eg, citation count and h-index). In health research, it is unclear whether social media impact also translates into research impact.

**Objective:**

The primary aim of this study was to systematically review the literature on the impact of using social media on the dissemination of health research. The secondary aim was to assess the correlation between Altmetrics and traditional citation-based metrics.

**Methods:**

We conducted a systematic review to identify studies that evaluated the use of social media to disseminate research published in health-related journals. We specifically looked at studies that described experimental or correlational studies linking the use of social media with outcomes related to bibliometrics. We searched the Medical Literature Analysis and Retrieval System Online (MEDLINE), Excerpta Medica dataBASE (EMBASE), and Cumulative Index to Nursing and Allied Health Literature (CINAHL) databases using a predefined search strategy (International Prospective Register of Systematic Reviews: CRD42017057709). We conducted independent and duplicate study selection and data extraction. Given the heterogeneity of the included studies, we summarized the findings through a narrative synthesis.

**Results:**

Of a total of 18,624 retrieved citations, we included 51 studies: 7 (14%) *impact studies* (answering the primary aim) and 44 (86%) *correlational studies* (answering the secondary aim). Impact studies reported mixed results with several limitations, including the use of interventions of inappropriately low intensity and short duration. The majority of correlational studies suggested a positive association between traditional bibliometrics and social media metrics (eg, number of mentions) in health research.

**Conclusions:**

We have identified suggestive yet inconclusive evidence on the impact of using social media to increase the number of citations in health research. Further studies with better design are needed to assess the causal link between social media impact and bibliometrics.

## Introduction

### Social Media and Its Impact in Health Research

Individual researchers and academic institutions use social media to disseminate their research findings to a broad audience that includes the public and health care practitioners. According to Carr and Hayes [1], social media can be broadly defined as “internet-based, disentrained, and persistent channels of mass personal communication facilitating perceptions of interactions among users, deriving value primarily from user-generated content”. For example, social media can be used by clinicians who would benefit from the findings in dealing with patients. A recent scoping review of the literature on social media use in health research revealed that social media are increasingly used to recruit patients, collect data, and establish and maintain user engagement, especially in the name of research dissemination [2]. At the same time, in the era of the internet and web 2.0 technologies that allow content generation and sharing [3], social media have been increasingly used as a source for measuring the impact of research, as they contribute to knowledge generation, dissemination, and translation [4]. Indeed, social media are increasingly required to measure their research performance and demonstrate the value of their research to governments and funding organizations [5]. Researchers in the domain of health and medical sciences have been particularly concerned about demonstrating the impact of their work as it usually bears implications for public health [6]. Hence, measuring the impact of health research is essential for influencing policy-making processes, improving health systems, and health-related socioeconomic impact [6]. According to the research impact framework (RIF) for health research [4], the domain of research-related impact is generally and traditionally evaluated according to conventional and traditional bibliometric approaches, which generally include the number of citations, the impact factor (IF), or the h-index [7]. Some academic institutions in the United States, Canada, and Europe started including social media impact as an evaluation criterion in their tenure and promotion policies [8]. According to the systematic review by Cruz Rivera et al [6] on the impact of health care research, researchers should consider indicators such as “the number of reads for published articles; article download rate and number of journal webpage visits; and citations rates in non-journal media such as newspapers and mass and social media (ie, Twitter and blogs)”.

Researchers can now discuss their papers and share their publications on various social networking sites [9] or with the *general public* interested in their topic. Using typology of social media by Constantinides and Fountain [10], health researchers can use 5 types of dissemination platforms: (1) blogs or web-based journals; (2) social networking sites, such as Facebook or Twitter, or professionally oriented platforms such as LinkedIn, ResearchGate, and Academia, which have recently emerged as social networking sites for academics [11]; (3) content communities; (4) forums or bulletin boards; and (5) content aggregators, such as Diigo, CiteULike, Delicious, Evernote, or through reference management software such as EndNote, Mendeley, RefWorks, Papers, and Zotero. These software companies have developed proprietary communities of users who share citations. In this way, an article can reach new and widespread audiences, broader than those of the limited subscribers of academic journals whose content is generally protected by paywalls. Some publishers have also embraced the movement of social media dissemination by including options for authors to semiautomatically share their output on academic content aggregators that include dashboards to measure the reach and impact of social media posts, based on the link or digital object identifier (DOI) associated with each article. Examples include free platforms such as the nonprofit-owned ImpactStory; or the profit-oriented Kudos, independent from publishing houses; or Publons, owned and managed by Clarivate Analytics; and PlumX [12], owned by Elsevier.

### Social Media Impact Measures

How is social media impact defined and measured? In the late 1990s, researchers started studying the phenomenon of internet-based dissemination of knowledge and information, forging the terms of *webometrics* or *cybermetrics* [13]. In 2010, a group of researchers defined a new set of metrics, Altmetrics (which stands for *alternative metrics*) that include web-based metrics (eg, number of link shares, likes, tweets, and views) and qualitative data that are complementary to traditional, citation-based metrics [14]. The so-called Altmetrics attention score (AAS) includes various indices of performance of a paper, such as the number of views; the number of discussions on social media (tweets, Facebook posts, and Wikipedia pages); recommendations (eg, Faculty of 1000); saved articles on popular social bookmarking services such as Mendeley or CiteULike; and the number of citations obtained from Google Scholar, CrossRef, PubMed Central, and Scopus [15]. Despite some intrinsic limitations—as the AAS can be inflated by self-citations, automatic retweeting, or sharing on various social media platforms [7]—Altmetrics is considered the current standard for measuring the impact of research on the internet and beyond [14-16].

Similar to the number of citations, the number of social media mentions of an article is a function of time since its publication. In 2011, Eysenbach published, in this journal, a seminal paper entitled, *Can tweets predict citation metrics on social media* [17]. The author proposed a set of measures for social media impact that would account for the dimension of *time*. The paper introduced the concepts of *Tweetations*, *Twimpact* *factor*, and *Twindex*. Tweetations were defined as “citations in a tweet,” that is, tweets mentioning the exact journal article URL (hence excluding links to DOI, PubMed entries, or other links). The twimpact factor was defined as the “cumulative number of tweetations within n days after publication” (eg, tw7 means the total number of tweetations after 7 days) [17]. The Twindex (or tweetation index) was defined as a “metric ranging from 0 to 100 indicating the relative standing of an article compared to other articles.” It is based on a percentile, rank-ordering of an article by tweetations, relative to other articles published in the same journal, which were published around the same time [17]. The author recommended that papers investigating the relationship between citations and social media mentions should adjust for time since the publication of an article (or specify a timeframe when these metrics were obtained), journal type, seasonal variations, and other possible confounders that might explain a nonlinear distribution of social media mentions.

### Social Media and Bibliometrics

How is social media dissemination related to citations? Social media dissemination is generally associated with the higher reach of an article. Some research, sponsored by Academia.edu, showed that by sharing an article on this specific academic, social networking site, a researcher could receive up to 69% more citations over 5 years [18]. In Eysenbach's (2011) paper, the author found a positive correlation between social media and subsequent citations and social media [17]. However, to what extent is there a causal link between the use of social media and subsequent citations? In other words, what is the impact of social media on citations and bibliometric indicators? To the best of our knowledge, to date, there is only one experimental study, using a randomized controlled trial (RCT) design, that has clearly shown a causal relationship between subsequent citations and the dissemination of articles through a proprietary web-based distribution platform (TrendMD) [19]. Although TrendMD does not meet the definition of social media as intended in this paper [1], as it is not based on user-generated content, but on content that is pushed by an algorithm of sponsored recommendations, the study provides a useful benchmark. The authors found significant effects on citations and Mendeley saves for the intervention group (TrendMD) compared with the control (no diffusion on TrendMD) after 6 and 12 months [19]. In addition, the intervention had positive effects on citations at both 6 and 12 months for articles in the area of health and medical sciences [19].

However, evidence from systematic reviews on this matter seems to be scarce. A basic search for systematic reviews on Altmetrics on Google Scholar, on April 2, 2019 (in titles of articles) yielded only 24 results, 3 of which were reviews: 1 systematic review mapping the evidence for marketing research [20] and 2 literature reviews on Altmetrics used for generic scholarly research output [21,22]. In terms of health research, the review evidence is also limited. To the best of our knowledge, there is only one ongoing systematic review—whose protocol is registered in the International Prospective Register of Systematic Reviews (PROSPERO) [23]—that is aimed at evaluating the use of social media to disseminate research findings among health care professionals. A similar research conducted on April 2, 2019, on PubMed, yielded 18 hits, 2 of which were systematic reviews: the first focused on medical research output and reported significant associations between Altmetrics and traditional citations, without linking measures of impact [24]; the second is a systematic review of reviews and meta-analyses and focused on the evaluation of methodological quality in the domain of skin psoriasis [25]. The latter reported that a journal’s IF could predict the number of tweets, whereas the years of publication and number of Mendeley readers predicted the number of citations on Google Scholar. Nevertheless, the authors concluded there does not seem to be a connection between scientific quality, social media, activity, and article usage [25].

Therefore, the primary aim of this study was to systematically review the literature on the impact of using social media on the dissemination of health research. The secondary aim was to assess the correlation between Altmetrics and traditional citation-based metrics.

## Methods

### Protocol and Eligibility Criteria

We developed a priori protocol for this systematic review and registered it in the PROSPERO database (CRD42017057709) [23]. This review focuses on health research, which includes biomedical, epidemiological, clinical, public health, and health systems research [26]. The inclusion criteria were as follows:

- *Population*: The unit of analysis of this review is studies (ie, study reports) published in health-related journals, including primary and secondary research, and editorials.
- *Interventions/exposures*: For both aims, we included studies that evaluated the use of social media to disseminate health research, which reported the outcomes described below. In the protocol, we included examples of social media and web 2.0 applications, defined as “a group of internet-based applications that build on the ideological and technological foundations of web 2.0, and that allow the creation and exchange of user generated content” [27]. Web 2.0 applications included interactive websites, blogging platforms, social networking, and social sharing sites, as described in the *Introduction* section.
- *Comparator*: The comparator for the primary aim (assessing the impact) was not using social media.
- *Outcome measures*: To be included, studies had to report measures of research dissemination such as traditional bibliometrics, Altmetrics, or webometrics indicators. Traditional bibliometric indicators were defined as quantitative data and statistics to publications such as journal articles and their accompanying citation counts. Altmetrics were defined as web-sourced metrics and qualitative data that are complementary to traditional, citation-based bibliometrics [15]. Webometrics were defined as the study of the quantitative aspects of the construction and use of information resources, structures, and technologies on the web, by drawing on bibliometric and infometric approaches [13]. Studies were included if they measured Altmetrics about webometrics and traditional bibliometrics, without any restrictions to any specific types of metrics.
- *Study design*: Experimental studies (eg, RCTs and nonrandomized studies or cohort studies), case series, and case studies.
- *Publication type*: We included original research papers, including scientific meeting abstracts or research letters, if they contained sufficient information to fill the extraction forms.

### Search Strategy

We searched the following electronic databases on July 12, 2017: Medical Literature Analysis and Retrieval System Online (MEDLINE; access via Ovid), Excerpta Medica dataBASE (EMBASE; access via Ovid), and Cumulative Index to Nursing and Allied Health Literature (CINAHL; access via EBSCO). We updated the searches on August 22, 2019. We developed a search strategy with the help of a health information specialist with experience in systematic reviews. The search strategy encompassed 2 main concepts: *social media* (based on the search strategy of a related systematic review by one of the authors [28]) and impact on dissemination (including *bibliometrics*, *Altmetric* and *academic impact*). The search strategy used both keywords and medical subject heading terms. The search strategies used are provided in Multimedia Appendix 1.

We did not use language restrictions. We did restrict the search timeframe by using the start date of January 2005, the year of the introduction of the *web 2.0* concept [3]. We also reviewed the reference lists of relevant papers and searched our files for both published and unpublished studies.

### Selection Process

Before starting the selection process, we conducted calibration exercises to train the reviewers and clarify the eligibility criteria. In all, 3 reviewers (EWA, MC, and ASR) and 2 research assistants screened the titles and abstracts of identified references in duplicate and independently for potential eligibility.

We obtained the full texts for all references judged as potentially eligible by at least one reviewer through our local library. For articles not found through the library, we searched on Google, Google Scholar, ResearchGate, or Academia.edu to locate self-archived copies. We contacted corresponding authors via ResearchGate or emailed them to obtain a copy of the articles. Then, the same 3 reviewers screened the full texts in duplicate and independently for eligibility, using a standardized pilot-tested full-text screening form. Once acceptable interrater reliability was achieved, the reviewers completed the selection tasks independently. All disagreements were resolved by discussion and with the help of a third reviewer (MB), who double-checked all decisions and confirmed the reasons for exclusion.

We assessed the interrater reliability for titles and abstract screening using Gwet AC1 index, as it is less prone to bias when there is a large disproportion in binary categories (ie, excluded vs included articles) that are not judged as relevant [29,30]. We assessed the interrater reliability for full-text screening using the kappa statistic.

### Data Abstraction Process

The review teams abstracted data from eligible studies in duplicate and an independent manner, using a standardized and pilot-tested data abstraction form with detailed instructions. Disagreements were resolved through discussions and with the help of a third reviewer (MB and EAA). Any inconsistencies in the abstraction tables were discussed within the research team (MB, EAA, RER, EWA, MC, and ASR) until consensus was reached. The abstracted data items included:

1. general information about the report, such as the first author’s name, year of publication, type of study (eg, experimental, cross-sectional, cohort, or qualitative), health area, journal, population/unit of analysis, sources of data, and period of investigation;
2. metrics reported, such as type of social media used and type of metrics (eg, social media metrics, such as Altmetric attention score; bibliometrics, such as citations; and webometrics, such as page views and number of downloads);
3. results, as reported by the study authors; and
4. funding and reported conflicts of interest.

For experimental studies, we collected additional specific information about the intervention (eg, sample size, frequency and reach of the intervention, duration and frequency of the intervention, and profile owner) and control conditions, where applicable. We also extracted the information about effects (eg, *F* tests and *t* tests) and *P* values, as reported by the authors. We used a web-based effect size calculator [31] to estimate effect sizes if they were not included in the original publication. One author (MB) checked all abstraction tables for consistency.

When correlations between social media metrics and citations were reported, following Eysenbach recommendations [17], we also extracted details about whether the paper: (1) reported social media metrics adjusted for time (eg, Twimpact factor) or provided a rationale for selecting a timeframe to assess the relationship, (2) included social media metrics that adjust for some kind of confounders (eg, using Twindex metric or stratifying by article type and/or topic), (3) the type of correlation test used (eg, using Spearman rho and/or Pearson *r*), and (4) explored the correlation using scatterplots or employed tests for nonlinear relationships (eg, log-linear and/or nonparametric tests).

### Data Synthesis

Given the heterogeneity of the included studies in terms of characteristics of the population, health area, study design, and reported outcomes (including *P* values and correlation coefficients), we summarized the findings through a narrative synthesis. In the summary tables included in this review, we reported *P* values and correlation coefficients and measures of effect sizes, as explicitly mentioned by the authors of the selected studies. We followed *Journal of Medical Internet Research’s* convention for reporting *P* values (3 digits) and correlation coefficients (2 digits). We included the text of the original source in quotation marks. For studies reporting correlations between social media and citation metrics, we defined the methodological quality of the paper using 4 indicators, using Eysenbach (2011) paper as a benchmark [17]: (1) appropriately adjusting for time, (2) appropriately adjusting for confounders, (3) appropriately exploring correlations, and (4) appropriately reporting nonlinear correlations tests and statistics. Appropriately adjusting for time means that the article accounted for the variability in the metric by time or specified a time when the social media metric was obtained about the time of data analysis. Appropriately adjusting for confounders means that the social media metric was adjusted for confounders, such as journal type, article type, and for the journal and the season, for example by using the Twindex metric, which is a percentile ranking relative to other articles published in the same journal and the same period. Appropriately exploring correlations involves the authors checking for correlations between social media metrics and bibliometrics by inspecting scatterplots. Appropriate reporting of tests for nonlinear correlations, such as Spearman rho correlations, log-linear tests of relationships, or other statistics, was based on ranking for non-normal distributions in the citations and social media mentions. We used the following scoring convention: (1) accounting for the time when selecting the data timeframe or acknowledging the role of time since publication (+) and adjusting the social media metric for time (++); (2) appropriately adjusting for confounders such as article type, topic, and/or subject (+) and seasonality or time factors using Twindex or similar metrics that account for the relative ranking of the article to the journal and season (++); (3) appropriately exploring correlations by including scatterplots (+); and (4) appropriately reporting nonlinear correlation tests and statistics (+) as well as log-linear relationship tests (++).

## Results

### Study Selection

**Figure 1:**
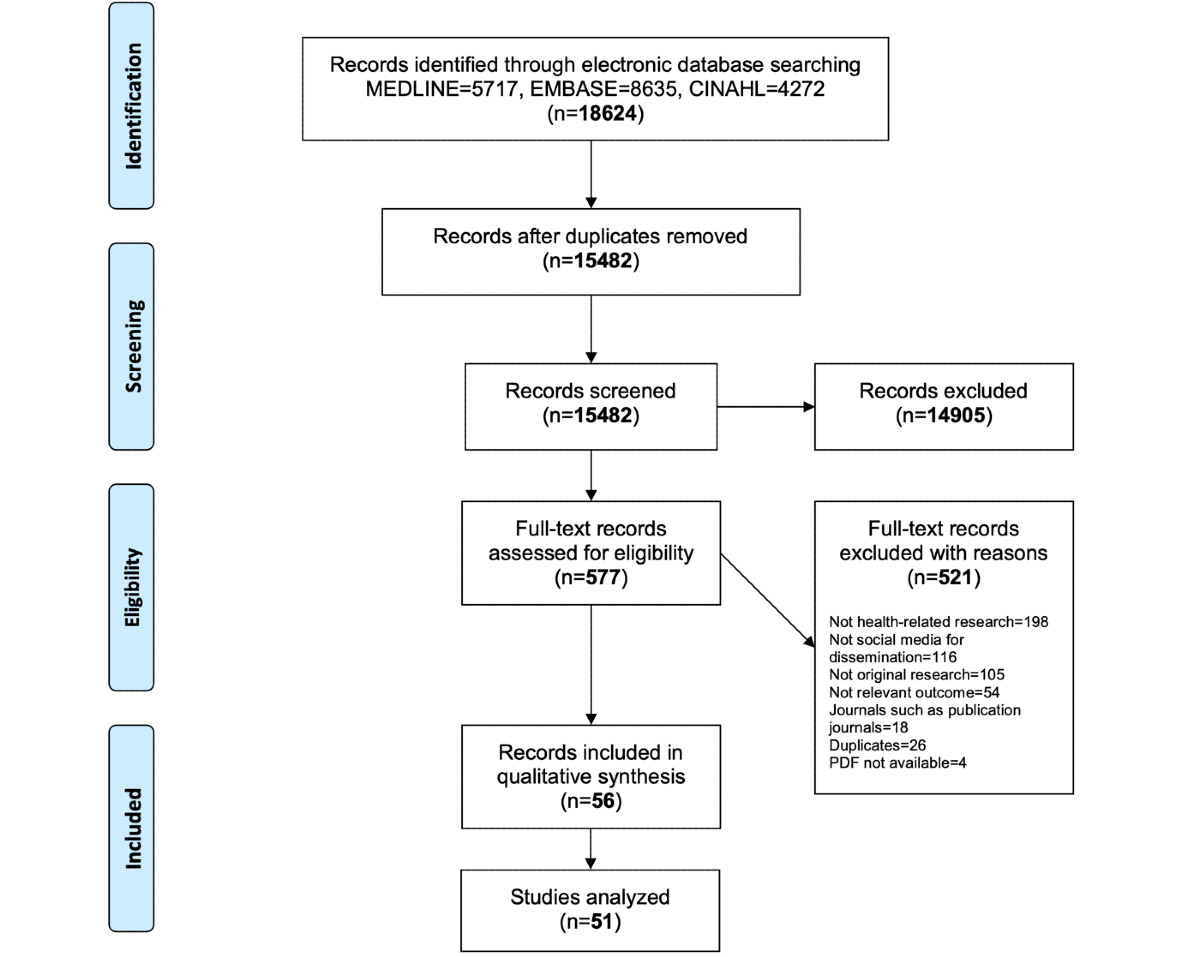
PRISMA flow diagram. MEDLINE=Medical Literature Analysis and Retrieval System Online, EMBASE=Excerpta Medica dataBASE, CINAHL=Cumulative Index to Nursing and Allied Health Literature.

Figure 1 shows the Preferred Reporting Items for Systematic Reviews and Meta-Analyses (PRISMA) flow diagram. Searches in the selected electronic databases yielded 13,576 records in July 2017 and 5048 in August 2019, totaling 18,624. Of these, 577 were selected for full-text screening, following a duplicate and independent selection process. The level of agreement at the title and abstract screening phase was high (mean Gwet AC1 0.96, SD 0.03). Similarly, we excluded 521 of 577 full-text records, achieving a substantial interrater agreement (mean Kappa 0.69, SD 0.22; mean Gwet AC1 0.77, SD 0.04). We excluded these 521 articles for the following reasons (a list of excluded records is provided in Multimedia Appendix 2): 198 did not discuss health-related research; 116 discussed social media application use, but not for disseminating research; 105 were not original research articles (ie, editorials, commentaries, conceptual papers, literature reviews, praising the use of social media for research dissemination); 54 did not report relevant study outcomes (ie, focusing either on Altmetrics, social media, or citation metrics separately); and 18 were records discussing the use of social media to increase the impact of a journal. Other reasons for exclusion were as follows: 26 were duplicates and 4 were citations of conference abstracts. After consensus-seeking discussions, we judged 56 records as eligible for inclusion in this systematic review, representing 51 unique studies, as 5 studies presented the same data in different publications (an abstract followed by a publication in a journal). The studies were by Amath [32,33], Hayon [34], Knight [35,36], Nolte [37,38], and O’Connor [39,40]. A total of 5 articles included only a conference abstract but were deemed to provide sufficient data for inclusion [41-45].

### Characteristics of the Included Studies

A total of 7 of 51 studies (14%) were categorized as *impact studies* [46-52], as they presented some interventions that tested the use of social media to disseminate research articles. The main characteristics of impact studies are summarized in Table 1. In all, 86% (44/51) studies were categorized as *correlational studies* [16,17,25,32,34,36,38,39,41-45,53-83], as they described the associations between traditional bibliometrics and Altmetrics. Correlational studies were classified according to the number of quality indicators as follows: *very good quality*, with 4/4 indicators (7/44, 16% studies; Table 2); *good quality*, with 3/4 indicators (8/44, 18% studies; Table 3); *fair quality*, with 2/4 indicators (10/44, 23% studies; Table 4); *poor quality*, with 1/4 indicators (12/44, 27% studies; Table 5); and *very poor quality*, with 0/4 indicators (7/44, 16% studies; Table 6). Detailed information of all 51 studies is included in Multimedia Appendix 3. In the next paragraphs, the results are presented separately, following the primary and secondary aims of this study.

**Table 1:** Characteristics of studies assessing the impact of social media interventions (n=7).

References	Health research area	Unit and period of analysis	Type of study	Social media interventions	Metrics reported	Results
Allen, 2013 [48]	Clinical pain sciences	16 original research articles from *PLOS ONE* (2006-2011)	Quasi-experimental (before-after)	Blog posts shared on Facebook, Twitter, LinkedIn, and ResearchBlogging.org	Citations (Scopus); HTML views and PDF downloads	Significant increase in HTML and PDF views; no significant effect on citations approximately 1 year after publication
Cawcutt 2019 [50]	Women’s health	8 original research articles published in 8 journals (2018)	Quasi-experimental (before-after)	Tweets chatted during a Physician’s Weekly tweet chat event (#PWChat)	Article downloads; AAS^a^	Increased AAS; increased downloads (statistical significance not reported)
Fox, 2015 [46]	Cardiology	243 articles, 121 intervention and 122 control, available from *Circulation* journal (2013-2014)	Experimental (RCT^b^)	Twitter and Facebook posts through the official circulation of social media accounts	HTML views and PDF downloads	No significant difference in 30 days’ HTML views (and downloads)
Fox, 2016 [47]	Cardiology	152 articles, 74 intervention and 78 control, available from *Circulation* journal (2015)	Experimental (RCT)	Twitter and Facebook posts through the official circulation of social media accounts	HTML views and PDF downloads	No statistically significant difference in 6-day or 30-day page views (and downloads)
Hoang, 2015 [52]	Radiology	2 research articles appearing on the *American Journal of Neuroradiology* and the *American Journal of Roentgenology* (2013-2014)	Quasi-experimental (retrospective cohort)	Blog posts on Radiopedia.org; podcast shared on Twitter and Facebook	HTML views and PDF downloads	Increased page views during the intervention; no increased activity beyond the podcast
Thoma, 2018 [51]	Emergency medicine	29 articles selected for intervention and control from the *Canadian Journal of Emergency Medicine* (2016)	Experimental (RCT)	Podcast or infographic or *standard social media promotion* through Twitter and Facebook	HTML views	Using podcasts and infographics was associated with increased Altmetric scores and abstract views but not full-text article views; they did not significantly increase full-text readership
Tonia, 2016 [49]	Public health	130 articles, 65 intervention and 65 control, from the *International Journal of Public Health*	Experimental (RCT)	*International Journal of Public Health* blog, Twitter, and Facebook accounts dissemination	Article abstract, PDF views, and downloads; citations; AAS	Number of downloads and the number of citations significantly correlated for all papers, with the correlation being stronger in the intervention group

^a^AAS: Altmetrics attention score.

^b^RCT: randomized controlled trial.

**Table 2:** Characteristics of correlational studies of very good quality (n=7).

Study ID and reference	Health research area/unit and period of analysis	Metrics reported	Results	Methodological quality indicators^a^			
				1	2	3	4
Costas, 2015 [61]	Biomedical and health sciences; 217,115 articles in health sciences available from WoS^b^ (2011-2013)	Social media: Altmetrics-Bibliometrics: Citations (WoS^b^)	Positive relationship between number of Altmetrics and the average citation impact and citation scores	+	+	+	+
Delli, 2017 [64]	Dental medicine; 100 articles with highest AAS^c^ from Altmetric Explorer and JCR^d^ (2015)	Social media: Altmetrics-Bibliometrics: Citations (Scopus)	No significant correlation between Altmetrics and citations	+	+	+	+
Eysenbach, 2011 [17]	Medical informatics; 208 tweets including links to 286 *JMIR*^e^ articles (2008-2010)	Social media: Twitter-Bibliometrics: Citations (Google Scholar and Scopus)	*Moderate* correlations	+	++	+	++
Haustein, 2014 [16]	Biomedical and health sciences; 1,431,576 biomedical and health sciences articles available on PubMed (2010-2012)	Social media: Twitter; Altmetrics-Bibliometrics: Citations (WoS^b^)	*Moderate* correlations	+	+	+	+
Knight, 2014 [35,36]	Organ transplantation; 6979 articles with citation data available; 1346 with social media mention (2011-2012)	Social media: Altmetrics-Bibliometrics: Citations (Scopus)	Significant correlations between social media mentions and citations	+	+	+	+
Livas, 2018 [71]	Orthodontics; Top 200 articles in orthodontics available from Altmetrics Explorer (2017)	Social media: Altmetrics-Bibliometrics: Citations (Scopus)	No correlation was observed between Altmetrics score and citations	+	+	+	+
Maggio, 2018 [72]	Health profession education; 2486 articles with Altmetrics published in health profession education (2013-2015)	Social media: Altmetrics-Bibliometrics: Citations (WoS^b^)	Significant correlations between Altmetrics and bibliometrics, but moderate effects	+	+	+	+

^a^1: appropriately adjusting for time of the social media metric (+); 2: appropriately adjusting for confounders such as article type (+) and seasonality/time factors (++); 3: appropriately exploring correlations by including scatterplots (+); 4: appropriately reporting nonlinear correlations tests and statistics (+) as well as log-linear relationship tests (++).

^b^WoS: Web of Science.

^c^AAS: Altmetrics attention score.

^d^JCR: Journal Citation Reports.

^e^JMIR: Journal of Medical Internet Research.

**Table 3:** Characteristics of correlational studies of good quality (n=8).

Study ID and reference	Health research area/unit and period of analysis	Metrics reported	Results	Methodological quality indicators^a^			
				1	2	3	4
Dal-Ré, 2017 [62]	Medical sciences; 410 original investigations and 182 opinion articles published in the first 4 printed issues of 4 top-ranked general medicine journals and 1 top-ranked journal on 5 different medical specialties that provide Altmetric scores (2015-2016)	Social media: Altmetrics-Bibliometrics: Citations (Google Scholar)	AAS^b^ was *strongly/moderately* associated with citation count	+	+	−	+
Haustein, 2015 [66]	Biomedical and health sciences; 1,339,297 articles, of which 595,254 in biomedical and health sciences, available from WoS^c^ (2012)	Social media: Altmetrics-Bibliometrics: Citations (WoS^c^)	No significant correlation between Altmetrics and citations	−	+	+	+
Jabaley, 2018 [67]	Sepsis research; Top 50 articles available on PubMed (via query; 2012-2017)	Social media: Altmetrics-Bibliometrics: Citations (Scopus and WoS^c^)	*Weak* to *moderate* correlations between Altmetrics and citations	+	+	+	−
O’Connor, 2017 [39,40]	Urology; Top 5 articles of top 10 journals in urology (2014-2015)	Social media: Altmetrics-Bibliometrics: Citations (Scopus)	*Weak* positive correlation between Altmetric score and citations	−	+	+	+
Rosenkrantz, 2017 [76]	Radiology; 892 articles from selected radiology journals (2013)	Social media: Altmetrics-Bibliometrics: Citations (WoS^c^)	Significant but *weak* correlation between the citation count and both the AAS^b^ and the number of Twitter mentions	−	+	+	+
Scotti, 2017 [78]	Not specified, hospital; 268 articles with Altmetric score out of 646 articles published in 2013 in indexed journals (with a 2012 IF^d^ score) by researchers affiliated to the authors’ hospital (2013)	Social media: Altmetrics-Bibliometrics: Citations (WoS^c^)	Altmetrics significantly associated with IF^d^ as well as Facebook, Twitter, and Mendeley	−	+	+	+
Thelwall, 2013 [81]	Not specified; 171-135,331 articles with nonzero Altmetric score and a valid PubMed ID (2011)	Social media: Altmetrics-Bibliometrics: Citations (WoS^c^)	Significant correlations between most Altmetrics and citations	+	+	−	++
Thelwall, 2016 [82]	Medical sciences; 290,282 articles from 45 fields in Scopus Medicine (2009)	Social media: Altmetrics-Bibliometrics: Citations (Scopus)	Significant correlations between Mendeley readers and citations	+	+	−	+

^a^1: appropriately adjusting for time of the social media metric (+); 2: appropriately adjusting for confounders such as article type (+) and seasonality/time factors (++); 3: appropriately exploring correlations by including scatterplots (+); 4: appropriately reporting nonlinear correlations tests and statistics (+) as well as log-linear relationship tests (++).

^b^AAS: Altmetrics attention score.

^c^WoS: Web of Science.

^d^IF: impact factor.

**Table 4:** Characteristics of correlational studies of fair quality (n=10).

Study ID and reference	Health research area/unit and period of analysis	Metrics reported	Results	Methodological quality indicators^a^			
				1	2	3	4
Araujo, 2018 [54]	Physiotherapy; 200 randomly selected articles from physiotherapy evidence database (PEDro; 2013-2016)	Social media: Altmetrics mentioned/reader-Bibliometrics: Citations (WoS^b^)	Significant correlation with citations	+	+	−	−
Calopedos, 2017 [58]	Urology; 22 urology articles in English language identified via PubMed (2010-2015)	Social media: Altmetrics-Bibliometrics: Citations (Google Scholar)	Significant correlation between Altmetrics and citations	+	−	+	−
Chang, 2019 [59]	Pediatric surgery; 140 articles appearing on 14 core journals on pediatric surgery (2012-2015)	Social media: Altmetrics-Bibliometrics: Citations (Scopus); IF^c^ (JCR^d^)	*Strong* correlations between Altmetric scores and citations, but not IF^c^	+	−	+	−
Dardas, 2019 [63]	Nursing; 100 articles in nursing with highest AAS^e^ from WoS^b^ (2012-2018)	Social media: Altmetrics-Bibliometrics: Citations (WoS^b^ and Scopus)	Significant *moderate* correlation between Altmetrics and citation counts	−	+	+	−
Hassona, 2019 [65]	Dental medicine; 100 articles with highest AAS^e^ from Altmetric Explorer (2018)	Social media: Altmetrics-Bibliometrics: Citations (Google Scholar and Scopus)	No significant correlation between Altmetrics and citations	−	+	−	+
Liu, 2013 [70]	Field not specified; 33,128 articles appearing in *PLOS One* (2011)	Social media: Altmetrics-Bibliometrics: HTML views, PDF downloads, and citations (Scopus, PubMed, and CrossRef)	Significant correlations between Altmetrics and bibliometrics	−	−	+	+
Nolte, 2019 [37,38]	Urology; 44 articles tweeted about the 2015 American Urological Association meeting (2015)	Social media: Twitter-Bibliometrics: IF^c^	Positive significant correlation with subsequent publication IF^c^ within 18 months of presentation	−	+	+	−
Punia, 2019 [73]	Neurological research; Top 100 articles from top 5 neurology journals (2016)	Social media: Altmetrics-Bibliometrics: Citations	*Weak* positive correlation between Altmetric score and citations	−	−	+	+
Quintana, 2016 [74]	Psychiatry; 438 articles in the *American Journal of Psychiatry* (2013-2015)	Social media: Twitter-Bibliometrics: Citations (WoS^b^)	Positive correlation between Twitter mentions and citations	+	−	−	++
Ruano, 2018 [25]	Psoriasis research; 164 systematic reviews or meta-analyses available from MEDLINE^f^, EMBASE^g^, and Cochrane databases (2016)	Social media: Altmetrics-Bibliometrics: Citations (Google Scholar)	No significant correlation between Altmetrics and citations; The number of Mendeley readers was significantly associated with citations	−	+	+	−

^a^1: appropriately adjusting for time of the social media metric (+); 2: appropriately adjusting for confounders such as article type (+) and seasonality/time factors (++); 3: appropriately exploring correlations by including scatterplots (+); 4: appropriately reporting nonlinear correlations tests and statistics (+) as well as log-linear relationship tests (++).

^b^WoS: Web of Science.

^c^IF: impact factor.

^d^JCR: Journal Citation Reports.

^e^AAS: Altmetrics attention score.

^f^MEDLINE: Medical Literature Analysis and Retrieval System Online.

^g^EMBASE: Excerpta Medica database.

**Table 5:** Characteristics of correlational studies of poor quality (n=12).

Study ID and reference	Health research area/unit and period of analysis	Metrics reported	Results	Methodological quality indicators^a^			
				1	2	3	4
Amath, 2017 [32,33]	Medical education: 482 articles appearing on Medical Education journal (2012-2013)	Social media: Twitter, Mendeley; Altmetrics-Bibliometrics: Citations (Scopus)	*Very strong* correlation between Tweet counts and Altmetrics score; Citations were *strongly* correlated with access counts and Mendeley downloads, and *weakly* and *moderately* correlated respectively with Twitter mentions and Altmetric scores	-	-	+	-
Azer, 2019 [55]	Medical professionalism; 50 most-cited articles in medical professionalism identified by searching WoS^b^ (1994-2011)	Social media: Altmetrics-Bibliometrics: Citations (WoS^b^)	No significant correlation between Altmetrics and citations	−	+	−	−
Baan, 2017 [56]	Transplantation; All articles published on transplantation in 2015 (volume 99)	Social media: Twitter-Bibliometrics: number of views and downloads	Significant correlation between downloads and Twitter activity	−	−	−	+
Batooli, 2016 [57]	Medical sciences; 533 articles published by faculty at Kashan University of Medical Sciences (1997-2014)	Social media: ResearchGate, Mendeley-Bibliometrics: Citations (Scopus)	Positive correlation between the number of views of articles in ResearchGate and citations; positive correlation between reading frequency in Mendeley and citations; number of views of articles in ResearchGate correlated with higher reading frequency in Mendeley and citations	−	−	−	+
Chen, 2019 [41]	Rheumatology; 1460 articles appearing in *Rheumatology* journal (2010-2015)	Social media: Altmetrics-Bibliometrics: Citations and downloads	*Strong* correlations between Altmetric and downloads, but not citations	−	+	−	−
Chiang, 2016 [42]	Gastroenterology; 1671 articles appearing on 5 core gastroenterology journals, 482 being tweeted (2012)	Social media: Twitter-Bibliometrics: Citations (Google Scholar)	No significant correlation between Twitter and citations	−	+	−	−
Cho, 2017 [60]	Medical sciences; 98 articles from medical sciences from Korean researchers in Scopus (2010-2014)	Social media: ImpactStory; Altmetrics-Bibliometrics: Citations (Scopus)	The more the papers are cited in the journal, the more papers saved on Mendeley	−	+	−	−
Hayon, 2019 [34]	Urology; 213 articles from 7 prominent urology journals (2014-2015)	Social media: Altmetrics-Bibliometrics: Citations (Google Scholar and Scopus)	Positive relationship between Twitter activity and Scopus citations	+	−	−	−
Jedhav, 2019 [68]	Neurointerventional surgery; 451 articles first published on the web on the *Journal of Neurointerventional Surgery* (2015-2016)	Social media: Twitter-Bibliometrics: Citations (WoS^b^)	The level of evidence of the publication and the topic of research strongly predicts future citations. The number of clicks also appears to be a strong predictor of future citations, and the number of clicks increases as the number of Twitter users also grows	−	−	+	−
Jeong, 2019 [69]	Coloproctology; 404 articles published on 3 journals with Twitter profiles (2015-2016)	Social media: Twitter-Bibliometrics: Citations (WoS^b^)	Significant correlations between citations and Twitter activity	−	+	−	−
Konstantiniuk, 2015 [44]	Sepsis research; 12 articles on sepsis compared with 8 articles on ICU^c^ (period not indicated)	Social media: Twittter; Altmetrics; ResearchGate-Bibliometrics: Citations (Google Scholar and WoS^b^)	The Altmetric score neither correlated with Google Citations nor publishing date	−	+	−	−
Shirazi, 2018 [79]	Health literacy; 615 articles with a digital object identifier and indexed in WoS^b^ (2015)	Social media: Altmetrics-Bibliometrics: Citations (WoS^b^)	Significant correlations between Altmetrics and citations	−	−	−	+

^a^1: appropriately adjusting for time of the social media metric (+); 2: appropriately adjusting for confounders such as article type (+) and seasonality/time factors (++); 3: appropriately exploring correlations by including scatterplots (+); 4: appropriately reporting nonlinear correlations tests and statistics (+) as well as log-linear relationship tests (++).

^b^WoS: Web of Science.

^c^ICU: Intensive Care Unit.

**Table 6:** Characteristics of correlational studies of very poor quality (n=7).

Study ID and reference	Health research area/unit and period of analysis	Metrics reported	Results	Methodological quality indicators^a^			
				1	2	3	4
Araujo, 2017 [53]	Parkinson disease research; Top 20 articles with highest AAS^b^ appearing on the *Journal of Parkinson's Disease* (2013-2016)	Social media: Twitter, Facebook, Altmetrics-Bibliometrics: Citations (Scopus)	Qualitative summary in support of correlation	−	−	−	−
Heydarpour, 2017 [43]	Multiple sclerosis research; 4693 articles on multiple sclerosis retrieved from Altmetric Explorer and PubMed (2016)	Social media: Altmetrics-Bibliometrics: Citations (WoS^c^)	*Moderate* correlations between AAS^b^ and citations	−	−	−	−
Matava, 2017 [45]	Pediatric anesthesiology; Top 100 articles on pediatric anesthesiology available from Altmetrics Explorer (2016)	Social media: Altmetrics-Bibliometrics: Citations (Scopus)	No significant correlation between Altmetrics or Twitter mentions and Citations; The number of Mendeley mentions was significantly associated with citations	−	−	−	−
Ramezani-Pakpour-Langeroudi, 2018 [75]	Clinical medicine; 55 highly cited articles on Thomson Reuters' Essential Science Indicator (2015)	Social media: ResearchGate, Mendeley, Academia, LinkedIn-Bibliometrics: Citations (Scopus)	A positive direct relationship was observed between visibility at social networking sites with citation and h‐index rate	−	−	−	−
Ruan, 2018 [77]	Plastic and reconstructive surgery; 55 most-cited articles published in *Plastic and Reconstructive Surgery* (2014-2015)	Social media: Altmetrics, Mendeley-Bibliometrics: Citations (Scopus)	No significant correlation between Altmetrics and citations; The number of Mendeley mentions was significantly associated with citations	−	−	−	−
Smith, 2019 [80]	Gastrointestinal endoscopy; 2361 original research articles published in G*astrointestinal Endoscopy* of which 2050 were cited at least once (2010-2016)	Social media: Altmetrics-Bibliometrics: Citations (Scopus)	Significant correlations between tweets and citations	−	−	−	−
Wiehn, 2017 [83]	Medical sciences; 36 Shire-sponsored articles (2016)	Social media: Altmetrics-Bibliometrics: Article downloads and IF^d^	No correlation was observed between Altmetrics score and IF^d^, downloads	−	−	−	−

^a^1: appropriately adjusting for time of the social media metric (+); 2: appropriately adjusting for confounders such as article type (+) and seasonality/time factors (++); 3: appropriately exploring correlations by including scatterplots (+); 4: appropriately reporting nonlinear correlations tests and statistics (+) as well as log-linear relationship tests (++).

^b^AAS: Altmetrics attention score.

^c^WoS: Web of Science.

^d^IF: impact factor.

### Is There Evidence of the Impact of Social Media?

In this section we elaborated on the findings related to the different areas of research, study type, social media intervention characteristics, metrics assessed, and reported results, as summarized in Table 1.

#### Areas of Health Research

Impact studies have reported the use of social media interventions to promote original research articles published in academic journals in the subject areas of clinical pain sciences (ie, *PLOS ONE*) [48], cardiology (ie, *Circulation*) [46,47], radiology [52], emergency health [51] public health (ie, *International Journal of Public Health*) [49], and women’s health (various journals) [50].

#### Study Types

We identified 4 randomized controlled experiments, further referred to as *RCTs* [46,47,49,51], and 3 quasi-experimental trials [48,50,52].

#### Social Media Intervention Characteristics

Articles in the intervention conditions were shared on Twitter or Facebook social media profiles of the targeted journals using automated or manually made posts leveraging the organic (ie, unpaid) reach of each social networking site. One study used advertising to increase the views of Facebook posts (ie, *boosted* content) to increase reach, which is the number of viewers of the post [47]. Intervention duration ranged from 12 [49], 18 [48], 34 [47], to 52 weeks [46]. The intensity varied considerably: only once the content was blogged [48], 1 post every 2 weeks and 12 weeks [49], 1 or 2 posts per week [46], or several posts per day [47]. Three studies used blogs to diffuse the studies further [48,49,52]. Thoma et al [51] tested the use of a podcast and infographics as complementary information in addition to Facebook and Twitter content sharing. The interventions varied in nature of the message posted, the profile of the social media owner(s), the duration of the posting, its intensity, and whether an incentive or paid promotion was used. For example, in one of the quasi-experimental trials [48], the authors advertised the posts related to 16 original research articles about clinical pain sciences, published on *PLOS ONE*, through a systematic intervention targeting Facebook, Twitter, LinkedIn, and ResearchBlogging.org. The authors used a systematic protocol (ie, timing the frequency of release of the messages) to direct social media users to read a web-based version of the original research article.

#### Metrics Assessed

These studies investigated the effect of social media interventions on subsequent access to web-based journal content and article downloads. Two studies (1 experimental [49] and 1 quasi-experimental [48]) reported the effect on the number of citations.

#### Reported Results

Although the quasi-experimental trials reported an increase in HTML and PDF views during the intervention [48,50,52], the RCTs reported no significant changes in the same metrics [46,47,49,51]. There were no significant effects on the number of citations in both the quasi-experiment [48] and the RCT [49].

### What Is the Association Between Altmetrics and Bibliometrics in Health Research?

In this section we present the results related to the correlational studies identified.

#### Areas of Health Research

The 44 correlational studies evaluated the relationship between Altmetrics and bibliometrics in a variety of health research fields and disciplines, covering generic medical or biomedical research disciplines [16,57,60-62,66,70,78,81-83], or more specific disease-related research fields, such as multiple sclerosis [43], neurological research [73], Parkinson disease [53], psoriasis [25], and sepsis [44,67]. Some articles covered clinical or dental medicine [64,65,75] and different kinds of surgery [59,68,77]; others focused on specialized branches of medicine, such as urology [34,38,39,58], radiology [76], and coloproctology [69].

#### Study Types

Almost all correlational studies were quantitative cross-sectional studies (43/44, 97%) examining the relationship between bibliometrics and various social media metrics or monitored citation trends over time [52,83]. The association between social media use and citations was also discussed in a qualitative study [53].

#### Metrics Assessed

Correlational studies encompassed a wide variety of Altmetrics and bibliometrics. Some correlational studies have investigated the association between Altmetrics scores and citations in Web of Science, Scopus, or Google Scholar [36,39,53,61,62,66,70,74,76,81-83]. One study investigated the correlation between ImpactStory indices and Scopus citations [60]. Other studies focused on the usage metrics of specific social media platforms, such as Twitter [17,42,56,74], blogs, web-based posts [52,58], ResearchGate, or Mendeley [57]. A total of 4 studies reported data on the association between the Altmetric of articles and the IF of journals in which they were published [38,59,60,83]; 3 studies investigated the relationship between sharing articles on academic social media platforms, such as ResearchGate and reference management software Mendeley [44,57,75].

#### Methodological Quality

As shown in the last 4 columns of Tables 2 to 6, the methodological quality of the papers varied according to the type of article and amount of detail included in the publication. The 5 conference abstracts included in the list of correlational studies [41-45] did not provide sufficient information to meet most of the methodological quality indicators. Only 3 of these [41,42,44] provided different correlation results according to the type of disease area, topic, or article type; hence, they were deemed to have adjusted for confounders. Of the remaining 39 studies with full-text, 16 adjusted the social media metric for time (16/39, 41%); 22 studies adjusted for confounders such as article type or topic (22/39, 56%), but none included the seasonality and timeframe of the article publication. Some 21 studies included scatterplots to explore the type of correlation (21/39, 54%), and 18 studies reported the use of Spearman rho or other nonparametric tests when comparing social media citation counts (18/39, 46%). However, only 2 studies [74,81] reported analyses for tests based on ranking similar to log-linear correlations included in a study by Eysenbach [17]. The method used in a study by Quintana and Doan [74], but described in the paper by Thelwall et al [81], “compares a given publication against the publications that appeared immediately before and after it. A successful test occurs when the number of Twitter mentions and citations for a given publication are both higher (or both lower) than the average of Twitter mentions and citations of the two adjacent articles” [81].

A total of 6 of 39 studies (15%) [16,36,61,64,71,72] met the minimum standard in all 4 criteria mentioned above, but none followed exemplar paper by Eysenbach [17] when reporting correlations between social media metrics and citations. In all, 7 studies scored at least three criteria (7/39, 18%). Of these studies, 4 scored criteria 2-3-4 [40,66,76,78], 2 studies scored 1-2-4 [62,82], and 1 study 1-2-3 [67]. The remaining 18 studies scored less than 2 of any quality criteria (18/39, 46%).

#### Reported Results

Twitter was the most popular social media platform discussed in correlational studies (21 of 44 studies) [16,17,32,34,38,42,45,53,55,56,59,62,63,68,69,72,74,76,78-80], followed by Mendeley (15 studies) [25,45,55,57,60,63,71,72,75-79,82,83]. In reference to the association between Altmetric scores and bibliometrics, the results were mixed. No significant correlations were reported in 12 of 44 studies (27%) [25,41,42,44,45,55,64-66,71,77,83]; *weak* or *moderate* correlations were reported by authors in 13 of 44 studies (30%) [16,17,39,43,53,61-63,67,72-74,76]. *Strong* positive associations were reported by the authors of half (22) of the correlational studies. In particular, strong associations were found between Mendeley readership (reads and downloads) and subsequent citations [25,45,57,60,75,77-79,82]. This is also consistent with the findings reported in one of the impact studies [49], which found significant correlations between the number of downloads of a paper and the subsequent number of citations (r=0.52; *P*<.001), which were larger in the intervention group (r=0.67; *P*<.001). Only 2 studies reported no significant associations between traditional and social media metrics [66] and between Altmetric score and journal IF, ResearchGate reads, and the number of article downloads [83].

When focusing only on the 7 high quality studies (ie, those meeting all methodological quality criteria 1-4) [16,17,36,61,64,71,72], 5 studies reported correlation coefficients of moderate size [16,17,36,61,72] and 2 studies, from the same discipline (orthodontics), reported no significant correlations [64,71].

## Discussion

### Is There Evidence of the Impact of Social Media?

The primary aim of this review was to evaluate the effect of social media on the dissemination of health research output. Of the 51 identified studies, only 7 were experimental studies aimed at establishing a causal link between social media use and subsequent citations. The identified impact studies provide suggestive yet inconclusive evidence on the effect of using social media to increase the number of citations, thus contributing to the dissemination of health research according to traditional bibliometric indicators. This result is consistent with findings reported in a systematic review, which was aimed at describing the interactions between bibliometric factors and social media activity on the impact of reviews in the field of psoriasis [25]. The findings suggest that although Google Scholar citations were predicted by the number of readers on Mendeley and year of publication (ie, 2015 and 2016), the number of tweets and the IF of a journal were not. Moreover, a journal's IF was the sole significant predictor of the number of tweets [25]. Careful considerations should be made, as the limited number of studies we identified does not allow us to generate strong conclusions or recommendations.

In our review, we identified four impact studies [46,47,49,51], which used randomized controlled experiments. However, unlike in the TrendMD dissemination study [19], these RCTs did not show consistent effects of the social media dissemination strategies on citations over time. This finding might be due to several limitations. First, the experiments used *social media interventions*, which had very different intensities (eg, once vs several posts per day) and duration (ie, 12-52 weeks). This fact does not allow us to determine whether the effect was due to a *dose-response* or to mere exposure to the intervention. Consequently, the long-term effects of social media interventions are still unclear as the only long-term study (52 weeks) [46] reported no difference in median 30-day page views between the intervention and control conditions. However, a larger, longitudinal RCT on TrendMD distribution showed higher citation counts in health and medical science articles after 6 and 12 months than in a control group [19]. Second, the reported social media impact outcomes differed in terms of content and type of social media channel used, frequency and intensity of social media use, type of exposure, and unit of analysis. It is important to note that careful consideration should be made when comparing social media interventions across the spectrum of platforms and types of social networking sites. A journal’s or author’s Facebook page or Twitter handle that originally has 100,000 followers would very likely increase the reach of an article shared, compared with a page that has only 1000 followers. This finding could have implications on the effect of increased citations in journals that already have a high IF compared with journals that have a low IF (as the accounts of the former would have a higher number of followers). In other words, the *social media interventions* were too heterogeneous to compare among each another and to perform meta-analyses.

Future impact studies should maintain a rigorous study design; consistently report social media outcomes using standard Altmetric scores; provide better and more detailed explanations about the specific timeframes, exposure, frequency, and intensity of interventions for comparability. In other words, future studies should answer research questions such as to what extent does the frequency of social media posting influence short-term indicators (eg, number of PDF downloads) and long-term indicators (eg, Altmetric score and citations)? To what extent does posting on Mendeley and Twitter, as opposed to Twitter, have an impact on the Altmetric score and citations?

This information could help researchers specialized in systematic reviews to develop accurate evidence, including meta-analyses; the information could also be helpful for researchers aimed at testing different social media intervention strategies or at comparing similar methodologies in different domains or disciplines. Other factors that can explain the lack of findings are in the topics of the information shared on social media. Some very specific health disciplines have limited readership, as they require specific knowledge to understand the content that is shared.

### What Is the Association Between Altmetrics and Bibliometrics in Health Research?

Another finding of this review is that most of the available evidence focuses on describing correlations between traditional and social media metrics in health research. The included 44 correlational studies provide further support that, in general, the higher the AAS, the higher the subsequent citations will be. However, the studies reported wide variability in the magnitude of correlation coefficients and provided a variety of interpretations for the strength of these correlations, which warns some caution. Most reports did not provide in-depth evaluations of the correlations, including, for example, confidence intervals of the correlation coefficients; an analysis of the distribution of citation counts and social media metrics, which tend to violate the assumptions of normality; and the use of visual representations such as scatterplots, as recommended by some researchers [84,85]. We identified only 6 correlational studies [16,36,61,64,71,72] meeting the minimum methodological quality criteria (1-4) described in the methodology, in addition to the seminal paper by Eysenbach, 2011 [17]. Most notably and quite surprisingly, none of the identified 44 studies followed the recommendations and metrics suggested by Eysenbach. No paper included the Twindex or the Twimpact factor or correctly adjusted the social media metrics for the time since publication or seasonality in publication and for the skewed distribution of the metric, for example, by dichotomizing result or testing for log-linear correlations.

Twitter and Mendeley seem to be the indicators that contribute the most to the Altmetric score. Mendeley and ResearchGate were positively associated with subsequent citations [25,45,77,79]. Although Twitter can be used to disseminate research output among a broader, general public, the use of Mendeley and ResearchGate seems to be restricted to specific target audience of researchers or media professionals. Unsurprisingly, the more an article is shared on ResearchGate or Mendeley, the more it will be cited. This finding might indicate that researchers use reference management software (Mendeley) to organize their libraries for research purposes and then share their research on a specialized social networking site (ResearchGate). This software allows users to discover new *related research* because the platform itself suggests new evidence based on the users’ previous reads and mentions. Although this does not imply that research has been disseminated among the wider public, ResearchGate and Mendeley appear fundamental for the research community.

Our findings are consistent with some existing review evidence investigating the domain of medical research output [24], as well as other multidisciplinary research fields [21,22], or marketing [20]. Another review that focused on orthopedic research journals [86] reported that web-based mentions were weakly yet positively related to various bibliometric indices, such as the number of citations, journal IF, Eigenfactors, and h-index values for the first and last authors. In addition, a systematic review of reviews published in scientific journals related to skin psoriasis [25] found an association between Altmetrics and bibliometric indicators. The association between traditional bibliometrics (eg, number of citations) and social media metrics (eg, number of mentions) could be an indicator of a positive effect of using social media on research dissemination. However, not all correlational articles identified showed strong positive correlations. The association could be confounded by several factors, such as the value of the research paper or the popularity of the topic, as we have previously discussed. In other words, a high-value research paper could lead to both high exposure on social media and a high number of citations, depending on the intrinsic subject. An article discussing skin psoriasis, or another more common disease, might attract the attention of the wider general public, compared with coloproctology and neuro-interventional surgery, which attract audiences of specialized health care professionals. Moreover, researchers can discover articles to cite from other *traditional* sources, such as electronic databases and libraries. Once an article is published and cited, other researchers might want to share the discovery on their social networking sites. Hence, using social media to diffuse health research may generate a virtuous circle that can be beneficial for both individual researchers and journals, as this will result in an increased IF. We need more evidence of good quality experimental, *impact studies* rather than correlational studies to establish a causal link between social media use and impact on citations. We also need better reporting of correlational studies; following the suggestions included in the seminal paper by Eysenbach [17], researchers should strive to provide more insight (and data) on the actual distribution of bibliometrics and social media metrics analyzed so that more meaningful interpretations of the relationship between these variables could be drawn.

### Recommendations

Scientific impact is a multidimensional concept that cannot be adequately measured by a single indicator [87]. As the AAS suggests, social media impact is even more multidimensional, as it is linked to various web-based tools that a particular researcher can use. This researcher is also embedded in a unit within an institution [88]; the researcher’s work is then published in a journal, which has a specific and independent impact (IF, citations, and Altmetrics). The evaluation of the researcher’s scientific impact on social media should then take into account various dimensions and indicators and be aligned with more qualitative evaluation on other domains of the RIF [4], which includes policy, service, and societal impacts.

In conclusion, is social media dissemination worth the effort? On the basis of the findings of this review, we recommend researchers in health sciences to continue using social media to disseminate their research, as there is some data suggesting its long-term impact on citations following dissemination on the internet using paid services (eg, TrendMD study) [19]. Researchers should use popular social networking platforms, such as Twitter or Facebook, first to engage with the general public and the media, to design more citizen-oriented research and democratize their findings. Researchers should also use popular social networking sites to interact with peers and discuss their research as well as disseminate their findings. Researchers should also diffuse their work on specialized social networking sites for academics, such as Mendeley, ResearchGate, and Academia. The content shared on these networks is very likely to be cited, as researchers who are on Mendeley may use it as a reference management software. Mendeley and ResearchGate databases may be used in combination with traditional electronic databases for literature reviews and similar activities. Storing copies of articles on these platforms could increase the chances of a paper being cited in the future.

Although more rigorous longitudinal research needs to establish whether social media activity can be linked to increased citations, it is important to consider social media as useful tools to reach a wider public, not just specialized audiences. Researchers should put effort into translating knowledge for different target audiences, bearing in mind the users of each social media channel. The AAS can be a useful instrument that allows researchers and institutions to evaluate social media impact by distinguishing among the attention generated by specialized applications (Mendeley and ResearchGate) or by the wider public and traditional media (Facebook, Twitter, and blogs).

### Strengths and Limitations

This review sheds light on the use of social media to disseminate health research output. The main strength of this review is the use of standard methods of a systematic review, including a comprehensive search strategy, a duplicate approach to study selection and data abstraction, and detailed data abstraction. The main limitation is that we were not able to conduct a meta-analysis because of the substantial variability in the included studies. For example, the impact studies varied in terms of the interventions evaluated (eg, type of social media used, the message posted, and its duration and intensity) and the outcomes assessed.

### Conclusions

Our findings have implications for research in the field of health-related metrics. There is a need for more and better designed experimental studies testing the use of social media to increase the dissemination of health research. These studies should be of a randomized design, evaluate the appropriate use of social media, and assess a variety of outcomes (both all Altmetrics dimensions and traditional bibliometrics) over a meaningfully long period. For example, experimental trials could test different strategies to diffuse research articles on social media, by comparing paid (or *boosted*) content with nonpaid, *organic* posts. Other trials could include the diffusion of research article links on numerous social networking sites versus a limited number of sites. Once there is evidence of the effectiveness of using social media (as opposed to not using them), different approaches should be investigated.

Our findings also have implications for the practice of using social media for research dissemination. Researchers should not use social media for the sole purpose of increasing their research productivity (ie, number of publications), as there is currently no evidence to support such an effect. They can use it for other purposes, such as disseminating their findings to social media users. When using social media, they have to be attentive to details such as the content of the message, its frequency, and the use of incentives or paid promotions, as this could affect the reach of the posts.

## Appendix

Multimedia Appendix 1 Search strategies.

Multimedia Appendix 2 Records excluded at the full-text screening phase with full citation.

Multimedia Appendix 3 Detailed characteristics of included studies.

## Abbreviations

AAS: Altmetrics attention score
DOI: digital object identifier
IF: impact factor
PROSPERO: International Prospective Register of Systematic Reviews
RCT: randomized controlled trial
RIF: research impact framework
