# Supplementary material for: The Use of Social Media to Increase the Impact of Health Research: Systematic Review
Source: J Med Internet Res. 2020 Jul 6;22(7):e15607. doi: 10.2196/15607 (PMC7380994; doi:10.2196/15607)
Supplement: Multimedia Appendix 1 [file jmir_v22i7e15607_app1.docx]

## Multimedia Appendix 1: Search strategies

### Search strategy used in Medline (OVID)

Database: Ovid MEDLINE(R) Epub Ahead of Print, In-Process & Other Non-Indexed Citations, Ovid MEDLINE(R) Daily and Ovid MEDLINE(R) <1946 to Present>

Search Strategy:

--------------------------------------------------------------------------------

1 Social Media/ (3758)

2 ((social or digital) adj2 (medium or media* or network* or net-work* or bookmark* or book-mark* or application* or debate* or channel* or communication* or collaborat*)).mp. (28906)

3 Social Networking/ (1851)

4 (facebook or twitter* or tweet* or LinkedIn or pinterest).mp. (3882)

5 ((Google adj plus) or google%252B).mp. (8)

6 (Tumblr or Instagram or myspace or researchgate or academia or figshare or mendeley).mp. (5992)

7 Blogging/ (808)

8 (blog* or microblog* or weblog* or micro-blog*).mp. (2015)

9 (tout or wordpress or yammer or citeulike or zotero or evernote or delicious or Digg or picasa or youtube or Vimeo or reddit or snapchat).mp. (2738)

10 Webcasts as Topic/ (283)

11 (podcast* or pod-cast* or webcast* or web-cast*).mp. (1347)

12 (rss adj2 feed*).mp. (46)

13 Electronic Mail/ (2402)

14 listserv*.mp. (534)

15 (weibo or flickr).mp. (121)

16 ((virtual or content or video* or project* or audio or digital or online or forum* or web*) adj2 (world* or reality or place* or communit* or communicat* or collaborat* or sharing* or share*)).mp. (22006)

17 (web adj2 application*).mp. (2426)

18 (((user adj generated) or usergenerated) adj2 content*).mp. (115)

19 ((knowledge or internet) adj2 (share* or communicat* or sharing* or collaborat*)).mp. (3632)

20 (wikipedia or wiki*).mp. (1090)

21 "web 2.0".mp. (591)

22 analytic*.mp. (220935)

23 or/1-22 (289839)

24 exp Bibliometrics/ or bibliometric*.mp. (9566)

25 citometric*.mp. (32)

26 cybermetric*.mp. (3)

27 scientometric*.mp. (389)

28 infodemiology.mp. (65)

29 webometric*.mp. (12)

30 infometric*.mp. (3)

31 (altmetric* or (alternative adj2 metrics)).mp. (200)

32 ((citation* or publication*) adj2 (analysis or count* or impact or metric* or number* or pattern*)).mp. (6774)

33 ((research or scientific) adj2 (assessment* or productivity or evaluation or impact)).mp. (20917)

34 impact factor.mp. (4692)

35 Journal Impact Factor/ (2778)

36 h-index.mp. (711)

37 ((academic or publication* or citation* or metric* or article* or bibliographical) adj2 (ranking or impact or output or evaluation or productivity or production or assessment or analysis)).mp. (8712)

38 or/24-37 (41960)

39 Universities/ or universit*.mp. (337148)

40 Schools, Medical/ (23834)

41 (medical adj2 school*).mp. (46440)

42 academic medical center.mp. or exp Academic Medical Centers/ (89989)

43 (academic adj2 (medical or health) adj5 center*).mp. (24549)

44 faculty.mp. or Faculty/ or researcher*.mp. or investigator*.mp. (261304)

45 or/39-44 (633794)

46 efficency.mp. (62)

47 productivity.mp. or Efficiency/ (54526)

48 motivation.mp. or Motivation/ (95302)

49 Employee Performance Appraisal/ or performance*.mp. (827392)

50 Reward/ or promotion*.mp. or advancement*.mp. or incentive*.mp. or reward*.mp. (242187)

51 Achievement/ or achiev*.mp. (856809)

52 (appointment* or tenure* or compensation*).mp. (78454)

53 or/46-52 (1992716)

54 45 and 53 (91353)

55 23 and (38 or 54) (5095)

56 limit 55 to yr="2005 -Current" (4118)

### Search strategy used in Embase (OVID)

Database: Embase <1980 to 2017 Week 28>

Search Strategy:

--------------------------------------------------------------------------------

1 social media/ (8935)

2 ((social or digital) adj2 (medium or media* or network* or net-work* or bookmark* or book-mark* or application* or debate* or channel* or communication* or collaborat*)).mp. (38097)

3 social network/ (11028)

4 (facebook or twitter* or tweet* or LinkedIn or pinterest).mp. (4944)

5 ((Google adj plus) or google%2B).mp. (20)

6 (Tumblr or Instagram or myspace or researchgate or academia or figshare or mendeley).mp. (7372)

7 blogging/ (137)

8 (blog* or microblog* or weblog* or micro-blog*).mp. (2347)

9 (tout or wordpress or yammer or citeulike or zotero or evernote or delicious or Digg or picasa or youtube or Vimeo or reddit or snapchat).mp. (2996)

10 webcast/ (268)

11 (podcast* or pod-cast* or webcast* or web-cast*).mp. (1442)

12 (rss adj2 feed*).mp. (66)

13 e-mail/ (14699)

14 listserv*.mp. (795)

15 (weibo or flickr or wikipedia or wiki*).mp. (1525)

16 ((virtual or content or video* or project* or audio or digital or online or forum* or web*) adj2 (world* or reality or place* or communit* or communicat* or collaborat* or sharing* or share*)).mp. (33731)

17 (web adj2 application*).mp. (2943)

18 (((user adj generated) or usergenerated) adj2 content*).mp. (134)

19 ((knowledge or internet) adj2 (share* or communicat* or sharing* or collaborat*)).mp. (4546)

20 "web 2.0".mp. (740)

21 analytic*.mp. (361616)

22 or/1-21 (464444)

23 exp Bibliometrics/ or bibliometric*.mp. (5048)

24 citometric*.mp. (29)

25 cybermetric*.mp. (10)

26 scientometric*.mp. (502)

27 infodemiology.mp. (46)

28 (altmetric* or (alternative adj2 metrics)).mp. (226)

29 webometric*.mp. (19)

30 ((citation* or publication*) adj2 (analysis or count* or impact or metric* or number* or pattern*)).mp. (9269)

31 infometric*.mp. (4)

32 ((research or scientific) adj2 (assessment* or productivity or evaluation or impact)).mp. (23423)

33 impact factor.mp. (5564)

34 h-index.mp. (809)

35 journal impact factor/ (2648)

36 ((academic or publication* or citation* or metric* or article* or bibliographical) adj2 (ranking or impact or output or evaluation or productivity or production or assessment or analysis)).mp. (11998)

37 or/23-36 (46961)

38 university/ (106599)

39 universit*.mp. (575125)

40 medical school/ (51036)

41 (medical adj2 school*).mp. (68089)

42 academic medical center.mp. or exp Academic Medical Centers/ (95135)

43 (academic adj2 (medical or health) adj5 center*).mp. (16491)

44 faculty.mp. or Faculty/ or researcher*.mp. or investigator*.mp. (371841)

45 or/38-44 (876752)

46 efficency.mp. (164)

47 productivity/ (32931)

48 productivity.mp. (69930)

49 motivation.mp. or motivation/ (112446)

50 employee performance appraisal.mp. (25)

51 performance.mp. or performance/ (1197282)

52 reward/ or reward.mp. (45270)

53 (promotion* or advancement* or incentive*).mp. (228735)

54 Achievement/ or achiev*.mp. (1078220)

55 (appointment* or tenure* or compensation*).mp. (83793)

56 or/46-55 (2604578)

57 45 and 56 (137281)

58 22 and (37 or 57) (9464)

59 limit 58 to yr="2005 -Current" (7995)

***************************

### Search strategy used in CINAHL (EBSCO)

Interface - EBSCOhost Research Databases
Search Screen - Advanced Search Database - CINAHL Plus with Full Text

Limiters/expanders: Search modes: Boolean Phrase

| **#** | **Query** | **Results** |
| --- | --- | --- |
| S1 | (MH "Social Media") | 5,559 |
| S2 | (MH "Social Networking") | 1,126 |
| S3 | TI ((social or digital) N2 (medium or media* or network* or net- work* or bookmark* or book-mark* or application* or debate* or channel* or communication* or collaborat*)) | 5,948 |
| S4 | AB ((social or digital) N2 (medium or media* or network* or net- work* or bookmark* or book-mark* or application* or debate* or channel* or communication* or collaborat*)) | 11,881 |
| S5 | TI ((facebook or twitter* or tweet* or LinkedIn or pinterest) | 2,608 |
| S6 | AB ((facebook or twitter* or tweet* or LinkedIn or pinterest) | 1,899 |
| S7 | TI (Google N plus) | 366 |
| S8 | TI (Google N plus) | 366 |
| S9 | AB (Google N plus) | 366 |
| S10 | AB (Google N plus) | 366 |
| S11 | AB (Tumblr or Instagram or myspace or researchgate or academia or figshare or mendeley) | 1,406 |
| S12 | TI (Tumblr or Instagram or myspace or researchgate or academia or figshare or mendeley) | 668 |
| S13 | (MH "Blogs") OR "blog" | 3,014 |
| S14 | (MH "Blogs") OR AB (blog* or microblog* or weblog* or micro-blog*) | 3,276 |
| S15 | TI (blog* or microblog* or weblog* or micro-blog*) | 1,303 |
| S16 | AB (tout or wordpress or yammer or citeulike or zotero or evernote or delicious or Digg or picasa or youtube or Vimeo or snapchat or reddit) | 756 |
| S17 | TI (tout or wordpress or yammer or citeulike or zotero or evernote or delicious or Digg or picasa or youtube or Vimeo or snapchat or reddit) | 567 |
| S18 | (MH "Webcasts") OR "webcasts" | 119 |
| S19 | AB (podcast* or pod-cast* or webcast* or web-cast*) | 290 |
| S20 | TI (podcast* or pod-cast* or webcast* or web-cast*) | 405 |
| S21 | TI (rss N2 feed*) | 29 |
| S22 | AB (rss N2 feed*) | 41 |
| S23 | (MH "Electronic Mail") | 5,006 |
| S24 | (MH "Listserv") OR "listserv" | 886 |
| S25 | AB (weibo or flickr or wikipedia or wiki*) | 434 |
| S26 | TI (weibo or flickr or wikipedia or wiki*) | 414 |
| S27 | AB ((virtual or content or video* or project* or audio or digital or online or forum* or web*) N2 (world* or reality or place* or communit* or communicat* or collaborat* or sharing* or share*)) | 8,375 |
| S28 | TI ((virtual or content or video* or project* or audio or digital or online or forum* or web*) N2 (world* or reality or place* or communit* or communicat* or collaborat* or sharing* or share*)) | 4,559 |
| S29 | AB (web N2 application*) | 436 |
| S30 | TI (web N2 application*) | 13 |
| S31 | AB (user-generated N2 content*) | 37 |
| S32 | TI (user-generated N2 content*) | 2 |
| S33 | AB ((knowledge or internet) N2 (share* or communicat* or sharing* or collaborat*)) | 2,336 |
| S34 | TI ((knowledge or internet) N2 (share* or communicat* or sharing* or collaborat*)) | 259 |
| S35 | "web 2.0" | 587 |
| S36 | (MH "Data Analytics") |  |
| S37 | AB (analytic or analytics) | 7,371 |
| S38 | TI (analytic or analytics) | 1,931 |
| S39 | (MH "Bibliometrics+") OR "bibliometrics" | 3,645 |
| S40 | TI (citometric* or citometrics) | 1,171 |
| S41 | AB (citometric* or citometrics) | 2,460 |
| S42 | AB (scientometric* or infodemiology or webometric* or cybermetric* or infometric*) | 88 |
| S43 | TI (scientometric* or infodemiology or webometric* or cybermetric* or infometric*) | 65 |
| S44 | AB (altmetric* or (alternative N2 metrics)) | 93 |
| S45 | TI (altmetric* or (alternative N2 metrics)) | 62 |
| S46 | AB ((citation* or publication*) N2 (analysis or count* or impact or metric* or number* or pattern*)) | 2,401 |
| S47 | TI ((citation* or publication*) N2 (analysis or count* or impact or metric* or number* or pattern*)) | 385 |
| S48 | AB ((research or scientific) N2 (assessment* or productivity or evaluation or impact)) | 5,614 |
| S49 | TI ((research or scientific) N2 (assessment* or productivity or evaluation or impact)) | 1,579 |
| S50 | (MH "Journal Impact Factor") OR "impact factor" | 1,203 |
| S51 | "h-index" | 215 |
| S52 | AB ((academic or publication* or citation* or metric* or article* or bibliographical) N2 (ranking or impact or output or evaluation or productivity or production or assessment or analysis)) | 6,989 |
| S53 | TI ((academic or publication* or citation* or metric* or article* or bibliographical) N2 (ranking or impact or output or evaluation or productivity or production or assessment or analysis)) | 1,028 |
| S54 | S39 OR S40 OR S41 OR S42 OR S43 OR S44 OR S45 OR S46 OR S47 OR S48 OR S49 OR S50 OR S51 OR S52 OR S53 | 18,595 |
| S55 | (MH "Colleges and Universities") | 18,322 |
| S56 | AB (universit*) or TI (universit*) | 84,427 |
| S57 | (MH "Schools, Medical") | 3,886 |
| S58 | AB (medical N2 school*) or TI (medical N2 school*) | 6,472 |
| S59 | (MH "Academic Medical Centers") | 52,978 |
| S60 | AB (academic medical center) or TI (academic medical center) | 3,974 |
| S61 | AB (academic N2 (medical or health) N5 center*) | 4,122 |
| S62 | TI (academic N2 (medical or health) N5 center*) | 962 |
| S63 | (MH "Faculty") | 5,671 |
| S64 | AB (faculty) or TI (faculty) | 17,687 |
| S65 | S55 OR S56 OR S57 OR S58 OR S59 OR S60 OR S61 OR S62 OR S63 OR S64 | 163,927 |
| S66 | (MH "Productivity") | 8,168 |
| S67 | (MH "Motivation") | 26,472 |
| S68 | (MH "Employee Performance Appraisal") | 2,317 |
| S69 | AB (performance* or reward* or advancement* or efficiency or productivity or achiev* or motivation or promotion* or incentive* or appointment* or tenure* or compensation*) or TI (performance* or reward* or advancement* or efficiency or productivity or achiev* or motivation or promotion* or incentive* or appointment* or tenure* or compensation*) | 319,602 |
| S70 | (MH "Reward") | 2,942 |
| S71 | (MH "Promotion and Tenure") | 1,480 |
| S72 | (MH "Achievement") OR (MH "Academic Achievement") | 5,532 |
| S73 | (MH "Physician Incentive Plans") OR (MH "Employee Incentive Programs") | 2,290 |
| S74 | S66 OR S67 OR S68 OR S69 OR S70 OR S71 OR S72 OR S73 | 347,579 |
| S75 | S65 AND S74 | 22,046 |
| S76 | S1 OR S2 OR S3 OR S4 OR S5 OR S6 OR S7 OR S8 OR S9 OR S10 OR S11 OR S12 OR S13 OR S14 OR S15 OR S16 OR S17 OR S18 OR S19 OR S20 OR S21 OR S22 OR S23 OR S24 OR S25 OR S26 OR S27 OR S28 OR S29 OR S30 OR S31 OR S32 OR S33 OR S34 OR S35 | 55,168 |
| S77 | S76 AND (S75 OR S54) | 1,463 |
