# Supplementary material for: The Use of Social Media to Increase the Impact of Health Research: Systematic Review
Source: J Med Internet Res. 2020 Jul 6;22(7):e15607. doi: 10.2196/15607 (PMC7380994; doi:10.2196/15607)
Supplement: Multimedia Appendix 2 [file jmir_v22i7e15607_app2.docx]

## Multimedia Appendix 2: Records excluded at the full-text screening phase with full citation

Notes: References are in JMIR reference style. Reasons for exclusion are indicated in the heading of each section

### Not health related (n=198)

#### Altmetrics in general research (n=35)

1. Alternative measures gaining ground. CILIP Update. 2015:19-. PMID: 107783763. Language: English. Entry Date: 20150424. Revision Date: 20150712. Publication Type: Journal Article. Journal Subset: Computer/Information Science.

2. Allen R. Onward & upward. Biomedical Signal Processing and Control. 2009 Jan;4(1):1-. PMID: WOS:000263017000001. doi: 10.1016/j.bspc.2008.11.005.

3. Alperin JP. Ask Not What Altmetrics Can Do for You, But What Altmetrics Can Do for Developing Countries. Bulletin of the Association for Information Science & Technology. 2013;39(4):18-21. PMID: 104187573. Language: English. Entry Date: 20130710. Revision Date: 20150820. Publication Type: Journal Article. Journal Subset: Computer/Information Science.

4. Bean JR. Altmetrics in Scientific Research: Flash in the Pan or Transformative Innovation? World Neurosurg. 2017 Aug;104:993-5. PMID: 28532921. doi: 10.1016/j.wneu.2017.05.065.

5. Black EL. Web analytics: a picture of the academic library Web site user. Journal of Web Librarianship. 2009;3(1):3-14. PMID: 105349887. Language: English. Entry Date: 20090807. Revision Date: 20150818. Publication Type: Journal Article.

6. Blakeman K. Bibliometrics in a digital age: help or hindrance. Sci Prog. 2018 Sep 1;101(3):293-310. PMID: 30131092. doi: 10.3184/003685018X15337564592469.

7. Brigham TJ. An introduction to altmetrics. Med Ref Serv Q. 2014;33(4):438-47. PMID: 25316077. doi: 10.1080/02763869.2014.957093.

8. Buschman M, Michalek A. Are Alternative Metrics Still Alternative? Bulletin of the Association for Information Science & Technology. 2013;39(4):35-9. PMID: 104187578. Language: English. Entry Date: 20130710. Revision Date: 20150820. Publication Type: Journal Article.

9. Carpenter TA, Lagace N, Bahnmaier S. Developing Standards for Emerging Forms of Assessment: The NISO Altmetrics Initiative. The Serials Librarian. 2016;70(1-4):85-8. PMID: 116415437. Language: English. Entry Date: 20160707. Revision Date: 20161102. Publication Type: Article. Journal Subset: Computer/Information Science. doi: 10.1080/0361526x.2016.1157737.

10. Dinsmore A, Allen L, Dolby K. Alternative Perspectives on Impact: The Potential of ALMs and Altmetrics to Inform Funders about Research Impact. Plos Biology. 2014 Nov;12(11). PMID: WOS:000345627300012. doi: ARTN e1002003

10.1371/journal.pbio.1002003.

11. Duin D, King D, van den Besselaar P. Identifying audiences of e-infrastructures--tools for measuring impact. PLoS One. 2012;7(12):e50943. PMID: 23239995. doi: 10.1371/journal.pone.0050943.

12. Evers JLH, Williams AC. Altmetrics, huh? 2016.

13. Fichter D, Wisniewski J. Social Media Metrics: Tracking Your Impact. Online. 2009 Jan-Feb;33(1):54-7. PMID: WOS:000262345100011.

14. Garcia-Milian R, Norton HF, Tennant MR. The presence of academic health sciences libraries on Facebook: the relationship between content and library popularity. Med Ref Serv Q. 2012;31(2):171-87. PMID: 22559180. doi: 10.1080/02763869.2012.670588.

15. Hall N. The Kardashian index: a measure of discrepant social media profile for scientists. Genome Biol. 2014 Jul 30;15(7):424. PMID: 25315513. doi: 10.1186/s13059-014-0424-0.

16. Konkiel S. Altmetrics A 21st-Century Solution to Determining Research Quality. Online Searcher. 2013;37(4):11-5. PMID: 107969911. Language: English. Entry Date: 20130904. Revision Date: 20150712. Publication Type: Journal Article.

17. Kousha K, Thelwall M. Are wikipedia citations important evidence of the impact of scholarly articles and books? Journal of the Association for Information Science and Technology. 2017;68(3):762-79. PMID: 121236337. Language: English. Entry Date: 20170221. Revision Date: 20170222. Publication Type: Article. Journal Subset: Computer/Information Science. doi: 10.1002/asi.23694.

18. Kwok R. Research impact: Altmetrics make their mark. 2013.

19. Melling J, Hambleton P, Mantzavinos D, Keane M, Van der Bruggen B. Measuring Value. Journal of Chemical Technology and Biotechnology. 2017 Jan;92(1):7-8. PMID: WOS:000389443600001. doi: 10.1002/jctb.5142.

20. Memon AR. ResearchGate is no longer reliable: leniency towards ghost journals may decrease its impact on the scientific community. 2016.

21. Mounce R. Open Access and Altmetrics: Distinct but Complementary. Bulletin of the Association for Information Science & Technology. 2013;39(4):14-7. PMID: 104187574. Language: English. Entry Date: 20130710. Revision Date: 20150820. Publication Type: Journal Article. Journal Subset: Computer/Information Science.

22. Osterrieder A. The value and use of social media as communication tool in the plant sciences. Plant Methods. 2013 Jul 11;9. PMID: WOS:000321923800001. doi: Artn 26

10.1186/1746-4811-9-26.

23. Padgett L. Word of Mouth Matters. Information Today. 2016;33(8):19-. PMID: 118524032. Language: English. Entry Date: 20161006. Revision Date: 20170202. Publication Type: Article. Journal Subset: Computer/Information Science.

24. Peters HP, Dunwoody S, Allgaier J, Lo YY, Brossard D. Public communication of science 2.0-Is the communication of science via the "new media" online a genuine transformation or old wine in new bottles? Embo Reports. 2014 Jul;15(7):749-53. PMID: WOS:000338919900007. doi: 10.15252/embr.201438979.

25. Piwowar H. Altmetrics: Value all research products. 2013.

26. Priem J, Groth P, Taraborelli D. The altmetrics collection. PLoS One. 2012;7(11):e48753. PMID: 23133655. doi: 10.1371/journal.pone.0048753.

27. Tattersall A. Supporting the research feedback loop Why and how library and information professionals should engage with altmetrics to support research. Performance Measurement and Metrics. 2017;18(1):28-37. PMID: WOS:000401169700005. doi: 10.1108/Pmm-08-2016-0037.

28. Taylor D, Busch C, Patel N, Mushrock G, Martin S, Sjostedt P. Getting the word out: developing a multichannel social media strategy for publication-based initiatives. Current Medical Research and Opinion. 2014 Apr;30:S22-S. PMID: WOS:000334146500047.

29. Tomaiuolo N. Research blogging: outstanding in its field. CyberSkeptic's Guide to Internet Research. 2012;17(6):6-. PMID: 108127106. Language: English. Entry Date: 20120706. Revision Date: 20150712. Publication Type: Journal Article.

30. Trost MJ, Webber EC, Wilson KM. Getting the Word Out: Disseminating Scholarly Work in the Technology Age. Acad Pediatr. 2017 Apr;17(3):223-4. PMID: 28126611. doi: 10.1016/j.acap.2017.01.007.

31. Trueger NS, Thoma B, Hsu CH, Sullivan D, Peters L, Lin M. The Altmetric Score: A New Measure for Article-Level Dissemination and Impact. Ann Emerg Med. 2015 Nov;66(5):549-53. PMID: 26004769. doi: 10.1016/j.annemergmed.2015.04.022.

32. Vinyard M. Altmetrics: AN OVERHYPED FAD OR AN IMPORTANT TOOL FOR EVALUATING SCHOLARLY OUTPUT? Computers in Libraries. 2016;36(10):26-9. PMID: 120245545. Language: English. Entry Date: 20161221. Revision Date: 20161222. Publication Type: Article. Journal Subset: Computer/Information Science.

33. Warren HR, Raison N, Dasgupta P. The Rise of Altmetrics. JAMA. 2017 Jan 10;317(2):131-2. PMID: 28097363. doi: 10.1001/jama.2016.18346.

34. Williams C. WHAT ALTMETRICS CAN DO FOR YOU. inCite. 2017;38(1/2):28-. PMID: 120656084. Language: English. Entry Date: 20170120. Revision Date: 20170123. Publication Type: Article. Journal Subset: Australia & New Zealand.

35. Yeong CH, Abdullah BJJ. Altmetrics: The right step forward. 2012.

#### Bibliometrics or Altmetrics not related to health research (n=22)

1. Webometrics releases world ranking of repositories. Access (02175673). 2008 (64):13-. PMID: 105752509. Language: English. Entry Date: 20080627. Revision Date: 20150711. Publication Type: Journal Article.

2. Promoting research on social media has little impact. 2014.

3. Bornmann L. What do altmetrics counts mean? A plea for content analyses. Journal of the Association for Information Science and Technology. 2016;67(4):1016-7. PMID: 113880045. Language: English. Entry Date: In Process. Revision Date: 20170403. Publication Type: Article. Journal Subset: Computer/Information Science. doi: 10.1002/asi.23633.

4. Bornmann L, Marx W. The Wisdom of Citing Scientists. Journal of the Association for Information Science and Technology. 2014 Jun;65(6):1288-92. PMID: WOS:000335583900015. doi: 10.1002/asi.23100.

5. Butler JS, Kaye ID, Sebastian AS, Wagner SC, Morrissey PB, Schroeder GD, et al. The Evolution of Current Research Impact Metrics: From Bibliometrics to Altmetrics? Clin Spine Surg. 2017 Jun;30(5):226-8. PMID: 28338492. doi: 10.1097/BSD.0000000000000531.

6. Chandra A. Field-Weighted Citation Index (FWCI) and Mendeley provide informative metrics for evaluating publication uptake. Current Medical Research and Opinion. 2017 May;33:11-. PMID: WOS:000400586700018.

7. Fausto S, Machado FA, Bento LF, Iamarino A, Nahas TR, Munger DS. Research blogging: indexing and registering the change in science 2.0. PLoS One. 2012;7(12):e50109. PMID: 23251358. doi: 10.1371/journal.pone.0050109.

8. Heinemann MK. On metrics. Thorac Cardiovasc Surg. 2013 Aug;61(5):377-8. PMID: 23918700. doi: 10.1055/s-0033-1351655.

9. Kaser D. Just Imagine. Computers in Libraries. 2016;36(10):2-. PMID: 120245536. Language: English. Entry Date: 20161221. Revision Date: 20161222. Publication Type: Article. Journal Subset: Computer/Information Science.

10. Konkiel S, Scherer D. New Opportunities for Repositories in the Age of Altmetrics. Bulletin of the Association for Information Science & Technology. 2013;39(4):22-6. PMID: 104187575. Language: English. Entry Date: 20130710. Revision Date: 20150820. Publication Type: Journal Article. Journal Subset: Computer/Information Science.

11. Lindsay JM. PlumX from Plum Analytics: Not Just Altmetrics. Journal of Electronic Resources in Medical Libraries. 2016 2016;13(1):8-17. PMID: 114608068. Language: English. Entry Date: 20160420. Revision Date: 20190214. Publication Type: Article.

12. Mandavilli A. Peer review: Trial by Twitter. Nature. 2011 Jan 20;469(7330):286-7. PMID: 21248816. doi: 10.1038/469286a.

13. Memon AR. ResearchGate and Impact Factor: A step further on predatory journals. 2017.

14. Moed HF, Halevi G. Multidimensional assessment of scholarly research impact. Journal of the Association for Information Science and Technology. 2015 Oct;66(10):1988-2002. PMID: WOS:000361184500002. doi: 10.1002/asi.23314.

15. Neda Z, Varga L, Biro TS. Science and Facebook: The same popularity law! PLoS One. 2017;12(7):e0179656. PMID: 28678796. doi: 10.1371/journal.pone.0179656.

16. Orduna-Malea E, Martin-Martin AM, Delgado Lopez-Cozar E. Metrics in academic profiles: a new addictive game for researchers? Rev Esp Salud Publica. 2016 Sep 22;90:e1-e5. PMID: 27653216.

17. Ovadia S. When Social Media Meets Scholarly Publishing. Behavioral & Social Sciences Librarian. 2013;32(3):194-8. PMID: 104220625. Language: English. Entry Date: 20130904. Revision Date: 20150819. Publication Type: Journal Article. Journal Subset: Computer/Information Science. doi: 10.1080/01639269.2013.817886.

18. Ovadia S. ResearchGate and Academia.edu: Academic Social Networks. Behavioral & Social Sciences Librarian. 2014;33(3):165-9. PMID: 103988292. Language: English. Entry Date: 20140813. Revision Date: 20150819. Publication Type: Journal Article. Journal Subset: Computer/Information Science. doi: 10.1080/01639269.2014.934093.

19. Pooladian A, Borrego A. Twenty years of readership of library and information science literature under Mendeley's microscope. Performance Measurement and Metrics. 2017;18(1):67-77. PMID: WOS:000401169700008. doi: 10.1108/Pmm-02-2016-0006.

20. Scardilli B. Kudos: Increasing Research Impact. Information Today. 2014;31(4):29-. PMID: 103941412. Language: English. Entry Date: 20140508. Revision Date: 20150710. Publication Type: Journal Article.

21. Scotti V. [Bibliometrics and web use: the birth of altmetrics]. 2015.

22. Wacogne ID. Article metrics: measuring the impact and importance of papers. Arch Dis Child Educ Pract Ed. 2016 Jun;101(3):156-7. PMID: 26767783. doi: 10.1136/archdischild-2015-309312.

#### Bibliometrics not related to health research (n=33)

1. Agarwal A, Durairajanayagam D, Tatagari S, Esteves SC, Harlev A, Henkel R, et al. Bibliometrics: tracking research impact by selecting the appropriate metrics. Asian J Androl. 2016 Mar-Apr;18(2):296-309. PMID: 26806079. doi: 10.4103/1008-682X.171582.

2. Bohannon J. Google scholar wins raves - But can it be trusted? 2014.

3. Bounova G, de Weck O. Overview of metrics and their correlation patterns for multiple- metric topology analysis on heterogeneous graph ensembles. Physical Review E. 2012 Jan 30;85(1). PMID: WOS:000299990300002. doi: ARTN 016117

10.1103/PhysRevE.85.016117.

4. Butler JS, Kaye ID, Sebastian AS, Wagner SC, Morrissey PB, Schroeder GD, et al. The Evolution of Current Research Impact Metrics: From Bibliometrics to Altmetrics? Clin Spine Surg. 2017 Jun;30(5):226-8. PMID: 28338492. doi: 10.1097/BSD.0000000000000531.

5. Buttliere BT. Using science and psychology to improve the dissemination and evaluation of scientific work. Frontiers in Computational Neuroscience. 2014 Aug 19;8. PMID: WOS:000341951600001. doi: UNSP 82

10.3369/fncom.2014.00082.

6. Citrome L. How we rate: Is impact factor the most important measure? 2013.

7. Clobridge A. Tracking Progress With and Impact of Open Access and Open Data. Online Searcher. 2015;39(5):68-70. PMID: 109965307. Language: English. Entry Date: 20150930. Revision Date: 20151002. Publication Type: Article. Journal Subset: Computer/Information Science.

8. Cronin B. Metrics a la mode. Journal of the American Society for Information Science and Technology. 2013 Jun;64(6):1091-. PMID: WOS:000323377600001. doi: 10.1002/asi.22989.

9. Dasgupta P, Millar S, Lee N. Learning from The Lancet. BJU Int. 2015 Jun;115(6):847. PMID: 26018952. doi: 10.1111/bju.13167.

10. Dasgupta P, Millar S, Wixon J. Scientific impact and beyond. BJU Int. 2015 Dec;116(6):833. PMID: 26542322. doi: 10.1111/bju.13357.

11. Ellero NP, Horne AS, Son IK, Ragon B, Moody D. Maintaining and Enhancing a Customized Online Journal Web Service. Journal of Electronic Resources in Medical Libraries. 2011;8(3):225-33. PMID: 104684256. Language: English. Entry Date: 20111007. Revision Date: 20150819. Publication Type: Journal Article. doi: 10.1080/15424065.2011.601987.

12. Fenner M. What can article-level metrics do for you? 2013.

13. Fichman P, Rosenbaum H. SIG/SI 8th Annual Research Symposium a Success! Bulletin of the American Society for Information Science & Technology. 2013;39(3):25-7. PMID: 104262869. Language: English. Entry Date: 20130408. Revision Date: 20150711. Publication Type: Journal Article. Journal Subset: Computer/Information Science.

14. Fiske M, DebBurman S, Smith P, Davis S. Eukaryon: An undergraduate peer-reviewed and managed life sciences scholarship journal. Faseb Journal. 2010 Apr;24. PMID: WOS:000208675502640.

15. Franck G. Open access: A revolution in scientific publication? Or just a minor amendment of accessibility? 2012.

16. Gasparyan AY, Nurmashev B, Yessirkepov M, Udovik EE, Baryshnikov AA, Kitas GD. The Journal Impact Factor: Moving Toward an Alternative and Combined Scientometric Approach. J Korean Med Sci. 2017 Feb;32(2):173-9. PMID: 28049225. doi: 10.3346/jkms.2017.32.2.173.

17. Guilak F, Jacobs CR. The H-index: Use and overuse. Journal of Biomechanics. 2011 Jan 4;44(1):208-9. PMID: WOS:000286550500034. doi: 10.1016/j.jbiomech.2010.11.006.

18. Haddad FS. The impact factor: yesterday's metric? Bone Joint J. 2014 Mar;96-B(3):289-90. PMID: 24589780. doi: 10.1302/0301-620X.96B3.33905.

19. Handel MJ. Article-level metrics-it's not just about citations. 2014.

20. Handoll HH, Atkinson G. Snowballing citations. BMJ. 2015 Dec 14;351:h6309. PMID: 26666656. doi: 10.1136/bmj.h6309.

21. Hartelius EJ, Mitchell GR. Big data and new metrics of scholarly expertise. 2014.

22. Hicks D, Wouters P, Waltman L, de Rijcke S, Rafols I. Bibliometrics: The Leiden Manifesto for research metrics. Nature. 2015 Apr 23;520(7548):429-31. PMID: 25903611. doi: 10.1038/520429a.

23. Kapil A, Jain NC. Impact factor: Is it the ultimate parameter for the quality of publication? 2016.

24. Leydesdorff L. How are new citation-based journal indicators adding to the bibliometric toolbox? Journal of the American Society for Information Science & Technology. 2009;60(7):1327-36. PMID: 105356185. Language: English. Entry Date: 20090821. Revision Date: 20150820. Publication Type: Journal Article.

25. Masic I, Begic E. Evaluation of Scientific Journal Validity, It's Articles and Their Authors...International Conference on Informatics, Management, and Technology in Healthcare, July 2016, Athens, Greece. Studies in Health Technology & Informatics. 2016;226:9-16. PMID: 121613025. Language: English. Entry Date: 20170314. Revision Date: 20170314. Publication Type: Article. Journal Subset: Computer/Information Science. doi: 10.3233/978-1-61499-664-4-9.

26. Melero R. Altmetrics - a complement to conventional metrics. Biochem Med (Zagreb). 2015;25(2):152-60. PMID: 26110028. doi: 10.11613/BM.2015.016.

27. Ojala M. CONFERENCE corral. Online Searcher. 2014;38(2):62-4. PMID: 107899530. Language: English. Entry Date: 20140401. Revision Date: 20150712. Publication Type: Journal Article. Journal Subset: Computer/Information Science.

28. Thakuria B, Saikia P. Predatory publisher and impact factor: The murky landscape of scholastic publication. Indian J Med Microbiol. 2016 Jul-Sep;34(3):392-3. PMID: 27514972. doi: 10.4103/0255-0857.188372.

29. Walther A, van den Bosch JJ. FOSE: a framework for open science evaluation. Front Comput Neurosci. 2012;6:32. PMID: 22754522. doi: 10.3389/fncom.2012.00032.

30. Wiley DL. Web Metrics for Library and Information Professionals. Online Searcher. 2014;38(4):77-8. PMID: 103984820. Language: English. Entry Date: 20140805. Revision Date: 20150710. Publication Type: Journal Article.

31. Wilsdon J. We need a measured approach to metrics. Nature. 2015 Jul 9;523(7559):129-. PMID: WOS:000357695900004. doi: DOI 10.1038/523129a.

32. Zafar A, Bolger JC, Burke D, Coulter J, McKevitt K, Peirce C, et al. Conference presentation: A route to increased citation metrics? 2017.

33. Zimmermann J, Roebroeck A, Uludag K, Sack AT, Formisano E, Jansma B, et al. Network-based statistics for a community driven transparent publication process. Front Comput Neurosci. 2011 Dec 27;6:11. PMID: 22403537. doi: 10.3389/fncom.2012.00011.

#### Dissemination of research via Internet, conceptual documents (n = 4)

1. Brown HM, Cruz M, Mol BW. Social media is essential for research engagement: FOR: Facilitating stakeholder engagement is key! BJOG. 2017 May;124(6):902. PMID: 28429441. doi: 10.1111/1471-0528.14531.

2. Ford C, Tolmie D. Breaking the Limits of Time and Space: How #medlibs Are Collaborating via Twitter. Journal of the Canadian Health Libraries Association (JCHLA). 2015;36(2):90-. PMID: 108975101. Language: English. Entry Date: 20151013. Revision Date: 20151013. Publication Type: Article.

3. Huh S. Revision of the instructions to authors to require a structured abstract, digital object identifier of each reference, and author's voice recording may increase journal access. J Educ Eval Health Prof. 2013;10:3. PMID: 23755319. doi: 10.3352/jeehp.2013.10.3.

4. Kousha K, Thelwall M. Disseminating Research with Web CV Hyperlinks. Journal of the Association for Information Science and Technology. 2014 Aug;65(8):1615-26. PMID: WOS:000339066500007. doi: 10.1002/asi.23070.

#### Social media for career advancement (n=17)

1. Azer SA, Holen A, Wilson I, Skokauskas N. Impact factor of medical education journals and recently developed indices: Can any of them support academic promotion criteria? 2016.

2. Bean JR. Academic Output and Social Media: A Marriage of Opposites. World Neurosurg. 2016 Jun;90:651-3. PMID: 26862024. doi: 10.1016/j.wneu.2016.02.004.

3. Cameron CB, Nair V, Varma M, Adams M, Jhaveri KD, Sparks MA. Does Academic Blogging Enhance Promotion and Tenure? A Survey of US and Canadian Medicine and Pediatric Department Chairs. 2016.

4. Carpenter J. The role of networking and social media in career advancement. Faseb Journal. 2014 Apr;28(1). PMID: WOS:000346646705177.

5. Eisen JA, Maccallum CJ, Neylon C. Expert failure: re-evaluating research assessment. PLoS Biol. 2013 Oct;11(10):e1001677. PMID: 24115910. doi: 10.1371/journal.pbio.1001677.

6. Farnan JM, Paro JA, Higa JT, Reddy ST, Humphrey HJ, Arora VM. Commentary: The relationship status of digital media and professionalism: it's complicated. Acad Med. 2009 Nov;84(11):1479-81. PMID: 19858794. doi: 10.1097/ACM.0b013e3181bb17af.

7. Flexman J. Climbing the ladder in academia. IEEE Eng Med Biol Mag. 2007 Jul-Aug;26(4):8-9. PMID: 17672225. doi: 10.1109/memb.2007.384086.

8. Gast KM, Kuzon WM, Waljee JF. Bibliometric Indices and Academic Promotion within Plastic Surgery. Plastic and Reconstructive Surgery. 2014 Nov;134(5):838e-44e. PMID: WOS:000344546000021. doi: 10.1097/Prs.0000000000000594.

9. Hafler JP, Lovejoy FH, Jr. Scholarly activities recorded in the portfolios of teacher-clinician faculty. Acad Med. 2000 Jun;75(6):649-52. PMID: 10875511. doi: 10.1097/00001888-200006000-00018.

10. Iyengar R, Wang Y, Chow J, Charney DS. An integrated approach to evaluate faculty members' research performance. Acad Med. 2009 Nov;84(11):1610-6. PMID: 19858825. doi: 10.1097/ACM.0b013e3181bb2364.

11. Posner MH. The Internet and academic freedom. Science. 2012 Jul 6;337(6090):13. PMID: 22767899. doi: 10.1126/science.1226099.

12. Sarewitz D. The pressure to publish pushes down quality. Nature. 2016 May 12;533(7602):147. PMID: 27172010. doi: 10.1038/533147a.

13. Shukla SK, Dixit VK. Publication for promotion in medical academia. Indian J Gastroenterol. 2016 Sep;35(5):398-9. PMID: 27638704. doi: 10.1007/s12664-016-0689-1.

14. Smith DR. Assessing and Developing Early Careers in Academic Research: How Can We Improve the Current Systems? 2015.

15. Soares MB. Collaborative research in light of the prevailing criteria for promotion and tenure in academia. Genomics. 2015 Oct;106(4):193-5. PMID: 26232606. doi: 10.1016/j.ygeno.2015.07.009.

16. Walker EA, Petscavage-Thomas JM, Fotos JS, Bruno MA. Quality metrics currently used in academic radiology departments: results of the QUALMET survey. British Journal of Radiology. 2017;90(1071). PMID: WOS:000396597200027. doi: ARTN 20160827

10.1259/bjr.20160827.

17. Ziehm S, Fontaine DK. Clinical faculty: tips for joining the ranks. AACN Adv Crit Care. 2009 Jan-Mar;20(1):71-81. PMID: 19174639. doi: 10.1097/NCI.0b013e31819453ab.

#### Social media research (n=16)

1. Bornmann L, Haunschild R. How to normalize Twitter counts? A first attempt based on journals in the Twitter Index. 2016.

2. Eom YH, Puliga M, Smailovic J, Mozetic I, Caldarelli G. Twitter-Based Analysis of the Dynamics of Collective Attention to Political Parties. PLoS One. 2015;10(7):e0131184. PMID: 26161795. doi: 10.1371/journal.pone.0131184.

3. Farina-Henry E, Waterston LB, Blaisdell LL. Social Media Use in Research: Engaging Communities in Cohort Studies to Support Recruitment and Retention. Jmir Research Protocols. 2015 Jul-Sep;4(3). PMID: WOS:000362730300026. doi: UNSP e90

10.2196/resprot.4260.

4. Garrett BM, Cutting R. Using social media to promote international student partnerships. 2012.

5. Greene EA, Griffin AS, Whittle J, Williams CA, Howard AB, Anderson KP. Development and usage of eXtension's HorseQuest: an online resource. J Anim Sci. 2010 Aug;88(8):2829-37. PMID: 20382882. doi: 10.2527/jas.2010-2810.

6. Hammer MJ. Ethical Considerations When Using Social Media for Research. Oncology Nursing Forum. 2017 Jul;44(4):410-2. PMID: WOS:000426406400003. doi: 10.1188/17.Onf.410-412.

7. Haustein S, Bowman TD, Holmberg K, Tsou A, Sugimoto CR, Lariviere V. Tweets as impact indicators: Examining the implications of automated "bot" accounts on Twitter. Journal of the Association for Information Science and Technology. 2016 Jan;67(1):232-8. PMID: WOS:000368340100016. doi: 10.1002/asi.23456.

8. Ke Q, Ahn YY, Sugimoto CR. A systematic identification and analysis of scientists on Twitter. PLoS One. 2017;12(4):e0175368. PMID: 28399145. doi: 10.1371/journal.pone.0175368.

9. Khairuddin MA, Rao A. Significance of likes: Analysing passive interactions on Facebook during campaigning. PLoS One. 2017;12(6):e0179435. PMID: 28622350. doi: 10.1371/journal.pone.0179435.

10. Lee RB, Baring R, Maria MS, Reysen S. Attitude towards technology, social media usage and grade-point average as predictors of global citizenship identification in Filipino University Students. 2015.

11. Mohammadi E, Thelwall M, Kousha K. Can Mendeley bookmarks reflect readership? A survey of user motivations. Journal of the Association for Information Science and Technology. 2016;67(5):1198-209. PMID: 114604948. Language: English. Entry Date: 20160602. Revision Date: 20170501. Publication Type: Article. Journal Subset: Computer/Information Science. doi: 10.1002/asi.23477.

12. Nam HK, Bang SM, Rhie YJ, Park SH, Lee KH. Qualitative assessment of precocious puberty-related user-created contents on YouTube. Ann Pediatr Endocrinol Metab. 2015 Sep;20(3):143-9. PMID: 26512350. doi: 10.6065/apem.2015.20.3.143.

13. Orduña-Malea E, Torres-Salinas D, Delgado López-Cózar E. Hyperlinks embedded in twitter as a proxy for total external in-links to international university websites. Journal of the Association for Information Science and Technology. 2015;66(7):1447-62. PMID: 103107679. Language: English. Entry Date: 20151110. Revision Date: 20160630. Publication Type: Article. doi: 10.1002/asi.23291.

14. Phan TQ, Airoldi EM. A natural experiment of social network formation and dynamics. Proc Natl Acad Sci U S A. 2015 May 26;112(21):6595-600. PMID: 25964337. doi: 10.1073/pnas.1404770112.

15. Phillippi JC, Buxton M. TwitterTM as a study prompt: engaging adult learners on the go. 2014.

16. Yarkoni T. Designing next-generation platforms for evaluating scientific output: what scientists can learn from the social web. Frontiers in Computational Neuroscience. 2012 Oct 1;6. PMID: WOS:000309292500001. doi: ARTN 72

10.3389/fncom.2012.00072.

#### Social network analyses (n=4)

1. Benton DC, Fernandez Fernandez MP. Social network analysis: a tool for the identification of next generation trainers. Collegian. 2014;21(4):311-8. PMID: 25632728. doi: 10.1016/j.colegn.2013.08.001.

2. Jordan K. Separating and Merging Professional and Personal Selves Online: The Structure and Processes That Shape Academics' Ego‐Networks on Academic Social Networking Sites and Twitter. Journal of the Association for Information Science & Technology. 2019 2019;70(8):830-42. PMID: 137268236. Language: English. Entry Date: 20190704. Revision Date: 20190716. Publication Type: Article. Journal Subset: Computer/Information Science.

3. Valente TW. Network interventions. Science. 2012 Jul 6;337(6090):49-53. PMID: 22767921. doi: 10.1126/science.1217330.

4. Vanderelst D. Social Network Analysis As a Tool for Research Policy.[Erratum appears in PLoS Negl Trop Dis. 2016 Jan;10(1):e0004410; PMID: 26835684]. 2015.

#### Research on bibliometrics and Altmetrics (n=45)

1. Facebook: A Bibliographic Analysis of the PsycINFO Database. Journal of Instructional Psychology. 2012;39(1):63-5. PMID: 108096631. Language: English. Entry Date: 20120924. Revision Date: 20150712. Publication Type: Journal Article.

2. Alperin JP, Gomez CJ, Haustein S. Identifying diffusion patterns of research articles on Twitter: A case study of online engagement with open access articles. Public Underst Sci. 2019 Jan;28(1):2-18. PMID: 29607775. doi: 10.1177/0963662518761733.

3. Aung HH, Zheng H, Erdt M, Aw AS, Sin SCJ, Theng YL. Investigating Familiarity and Usage of Traditional Metrics and Altmetrics. Journal of the Association for Information Science and Technology. 2019 Aug;70(8):872-87. PMID: WOS:000486779500008. doi: 10.1002/asi.24162.

4. Bornmann L, Haunschild R. Overlay maps based on Mendeley data: The use of altmetrics for readership networks. Journal of the Association for Information Science and Technology. 2016 Dec;67(12):3064-72. PMID: WOS:000387878600017. doi: 10.1002/asi.23569.

5. Botting N, Dipper L, Hilari K. The Effect of Social Media Promotion on Academic Article Uptake. Journal of the Association for Information Science and Technology. 2017 Mar;68(3):795-800. PMID: WOS:000396413100020. doi: 10.1002/asi.23704.

6. De Gregori M, Scotti V, De Silvestri A, Curti M, Fanelli G, Allegri M, et al. Does a research group increase impact on the scientific community or general public discussion? Alternative metric-based evaluation. 2016.

7. Di Girolamo N, Reynders RM. Health care articles with simple and declarative titles were more likely to be in the Altmetric Top 100. 2016.

8. Didegah F, Bowman TD, Holmberg K. On the Differences Between Citations and Altmetrics: An Investigation of Factors Driving Altmetrics Versus Citations for Finnish Articles. Journal of the Association for Information Science and Technology. 2018 Jun;69(6):832-43. PMID: WOS:000434973900007. doi: 10.1002/asi.23934.

9. Didegah F, Thelwall M. Co‐saved, co‐tweeted, and co‐cited networks. Journal of the Association for Information Science & Technology. 2018 2018;69(8):959-73. PMID: 130954752. Language: English. Entry Date: 20180731. Revision Date: 20190801. Publication Type: Article. Journal Subset: Computer/Information Science.

10. Erdt M, Aung HH, Aw AS, Rapple C, Theng YL. Analysing researchers' outreach efforts and the association with publication metrics: A case study of Kudos. PLoS One. 2017 2017;12(8):e0183217. PMID: 28817627. doi: 10.1371/journal.pone.0183217.

11. Evans P, Krauthammer M. Exploring the use of social media to measure journal article impact. 2011.

12. Haunschild R, Bornmann L. Field- and time-normalization of data with many zeros: an empirical analysis using citation and Twitter data. Scientometrics. 2018 2018;116(2):997-1012. PMID: 30147201. doi: 10.1007/s11192-018-2771-1.

13. Hoffmann CP, Lutz C, Meckel M. A relational altmetric? Network centrality on ResearchGate as an indicator of scientific impact. Journal of the Association for Information Science and Technology. 2016;67(4):765-75. PMID: 113880056. Language: English. Entry Date: In Process. Revision Date: 20170403. Publication Type: Article. Journal Subset: Computer/Information Science. doi: 10.1002/asi.23423.

14. Huang W, Wang P, Wu Q. A correlation comparison between Altmetric Attention Scores and citations for six PLOS journals. PLoS One. 2018 2018;13(4):e0194962. PMID: 29621253. doi: 10.1371/journal.pone.0194962.

15. Katchanov YL, Markova YV, Shmatko NA. Comparing the topological rank of journals in Web of Science and Mendeley. Heliyon. 2019 Jul;5(7):e02089. PMID: 31388571. doi: 10.1016/j.heliyon.2019.e02089.

16. Lamb CT, Gilbert SL, Ford AT. Tweet success? Scientific communication correlates with increased citations in Ecology and Conservation. PeerJ. 2018 2018;6:e4564. PMID: 29666750. doi: 10.7717/peerj.4564.

17. Lin J, Fenner M. The Many Faces of Article-Level Metrics. Bulletin of the Association for Information Science & Technology. 2013;39(4):27-30. PMID: 104187576. Language: English. Entry Date: 20130710. Revision Date: 20150711. Publication Type: Journal Article.

18. Maflahi N, Thelwall M. When are readership counts as useful as citation counts? Scopus versus Mendeley for LIS journals. Journal of the Association for Information Science and Technology. 2016;67(1):191-9. PMID: 112228405. Language: English. Entry Date: 20170331. Revision Date: 20170331. Publication Type: Article. Journal Subset: Computer/Information Science. doi: 10.1002/asi.23369.

19. Marashi SA, Hosseini-Nami SM, Alishah K, Hadi M, Karimi A, Hosseinian S, et al. Impact of Wikipedia on citation trends. EXCLI J. 2013;12:15-9. PMID: 27034629.

20. Mastley CP. Social Media and Information Behavior: A Citation Analysis of Current Research from 2008–2015. Serials Librarian. 2017 2017;73(3):339-51. PMID: 126670056. Language: English. Entry Date: 20180103. Revision Date: 20190425. Publication Type: Article.

21. Mohammadi E, Thelwall M. Mendeley Readership Altmetrics for the Social Sciences and Humanities: Research Evaluation and Knowledge Flows. Journal of the Association for Information Science and Technology. 2014 Aug;65(8):1627-38. PMID: WOS:000339066500008. doi: 10.1002/asi.23071.

22. Mohammadi E, Thelwall M, Haustein S, Larivière V. Who reads research articles? An altmetrics analysis of Mendeley user categories. Journal of the Association for Information Science and Technology. 2015;66(9):1832-46. PMID: 108697294. Language: English. Entry Date: 20151012. Revision Date: 20160831. Publication Type: Article. doi: 10.1002/asi.23286.

23. Mula M. The impact and dissemination of scientific research: From impact factor to social media. The Top 10 articles in Epilepsy & Behavior published in 2014. Epilepsy Behav. 2015 Sep;50:113-5. PMID: 26262999. doi: 10.1016/j.yebeh.2015.07.012.

24. Peoples BK, Midway SR, Sackett D, Lynch A, Cooney PB. Twitter Predicts Citation Rates of Ecological Research. PLoS One. 2016;11(11):e0166570. PMID: 27835703. doi: 10.1371/journal.pone.0166570.

25. Peters I, Kraker P, Lex E, Gumpenberger C, Gorraiz J. Research data explored: an extended analysis of citations and altmetrics. Scientometrics. 2016;107:723-44. PMID: 27122647. doi: 10.1007/s11192-016-1887-4.

26. Pulido CM, Redondo-Sama G, Sorde-Marti T, Flecha R. Social impact in social media: A new method to evaluate the social impact of research. PLoS One. 2018 2018;13(8):e0203117. PMID: 30157262. doi: 10.1371/journal.pone.0203117.

27. Ringelhan S, Wollersheim J, Welpe IM. I Like, I Cite? Do Facebook Likes Predict the Impact of Scientific Work? 2015.

28. Robinson-Garcia N, Arroyo-Machado W, Torres-Salinas D. Mapping social media attention in Microbiology: identifying main topics and actors. FEMS Microbiol Lett. 2019 Apr 1;366(7):01. PMID: 30977791. doi: 10.1093/femsle/fnz075.

29. Saberi MK, Ekhtiyari F. Usage, captures, mentions, social media and citations of LIS highly cited papers: an altmetrics study. Performance Measurement and Metrics. 2019 2019;20(1):37-47. PMID: WOS:000469419400004. doi: 10.1108/Pmm-10-2018-0025.

30. Shema H, Bar-Ilan J, Thelwall M. Do blog citations correlate with a higher number of future citations? Research blogs as a potential source for alternative metrics. Journal of the Association for Information Science and Technology. 2014;65(5):1018-27. PMID: 103929976. Language: English. Entry Date: 20140416. Revision Date: 20150710. Publication Type: Journal Article. doi: 10.1002/asi.23037.

31. Shema H, Bar-Ilan J, Thelwall M. How is research blogged? A content analysis approach. Journal of the Association for Information Science and Technology. 2015;66(6):1136-49. PMID: 103797671. Language: English. Entry Date: 20150522. Revision Date: 20160531. Publication Type: Journal Article. doi: 10.1002/asi.23239.

32. Shrivastava R, Mahajan P. An altmetric analysis of ResearchGate profiles of physics researchers A study of University of Delhi (India). Performance Measurement and Metrics. 2017;18(1):52-66. PMID: WOS:000401169700007. doi: 10.1108/Pmm-07-2016-0033.

33. Shuai X, Pepe A, Bollen J. How the Scientific Community Reacts to Newly Submitted Preprints: Article Downloads, Twitter Mentions, and Citations. Plos One. 2012 Nov 1;7(11). PMID: WOS:000310601300002. doi: ARTN e47523

10.1371/journal.pone.0047523.

34. Snijder R. Revisiting an open access monograph experiment: measuring citations and tweets 5 years later. Scientometrics. 2016;109(3):1855-75. PMID: 27942082. doi: 10.1007/s11192-016-2160-6.

35. Sud P, Thelwall M. Not all international collaboration is beneficial: The Mendeley readership and citation impact of biochemical research collaboration. Journal of the Association for Information Science and Technology. 2016;67(8):1849-57. PMID: 116892587. Language: English. Entry Date: 20160804. Revision Date: 20160804. Publication Type: Article. Journal Subset: Computer/Information Science. doi: 10.1002/asi.23515.

36. Sugimoto CR, Thelwall M, Lariviere V, Tsou A, Mongeon P, Macaluso B. Scientists popularizing science: characteristics and impact of TED talk presenters. PLoS One. 2013;8(4):e62403. PMID: 23638069. doi: 10.1371/journal.pone.0062403.

37. Thelwall M, Kousha K. Academia.edu: Social Network or Academic Network? Journal of the Association for Information Science and Technology. 2014 Apr;65(4):721-31. PMID: WOS:000335583300007. doi: 10.1002/asi.23038.

38. Thelwall M, Kousha K. ResearchGate: Disseminating, communicating, and measuring Scholarship? Journal of the Association for Information Science and Technology. 2015;66(5):876-89. PMID: 103787831. Language: English. Entry Date: 20150417. Revision Date: 20160502. Publication Type: Journal Article. doi: 10.1002/asi.23236.

39. Thelwall M, Kousha K. ResearchGate articles: Age, discipline, audience size, and impact. Journal of the Association for Information Science and Technology. 2017 Feb;68(2):468-79. PMID: WOS:000394974000014. doi: 10.1002/asi.23675.

40. Thelwall M, Maflahi N. Are scholarly articles disproportionately read in their own country? An analysis of mendeley readers. Journal of the Association for Information Science and Technology. 2015;66(6):1124-35. PMID: 103797664. Language: English. Entry Date: 20150522. Revision Date: 20160531. Publication Type: Journal Article. doi: 10.1002/asi.23252.

41. Thelwall M, Sud P. Mendeley readership counts: An investigation of temporal and disciplinary differences. Journal of the Association for Information Science and Technology. 2016 Dec;67(12):3036-50. PMID: WOS:000387878600015. doi: 10.1002/asi.23559.

42. Xia F, Su X, Wang W, Zhang C, Ning Z, Lee I. Bibliographic Analysis of Nature Based on Twitter and Facebook Altmetrics Data. PLoS One. 2016;11(12):e0165997. PMID: 27906981. doi: 10.1371/journal.pone.0165997.

43. Yan KK, Gerstein M. The spread of scientific information: insights from the web usage statistics in PLoS article-level metrics. PLoS One. 2011;6(5):e19917. PMID: 21603617. doi: 10.1371/journal.pone.0019917.

44. Zahedi Z, Costas R. General discussion of data quality challenges in social media metrics: Extensive comparison of four major altmetric data aggregators. PLoS One. 2018 2018;13(5):e0197326. PMID: 29772003. doi: 10.1371/journal.pone.0197326.

45. Zheng H, Aung HH, Erdt M, Peng TQ, Raamkumar AS, Theng YL. Social media presence of scholarly journals. Journal of the Association for Information Science and Technology. 2019 Mar;70(3):256-70. PMID: WOS:000458834600005. doi: 10.1002/asi.24124.

#### Research on bibliometrics only (n=15)

1. Journal performance report. Ann Emerg Med. 2014 Sep;64(3):219-21. PMID: 25149962. doi: 10.1016/j.annemergmed.2014.07.023.

2. Bar-Ilan J. JASIST 2001-2010. Bulletin of the American Society for Information Science & Technology. 2012;38(6):24-8. PMID: 104505983. Language: English. Entry Date: 20120914. Revision Date: 20150820. Publication Type: Journal Article.

3. Bernstam EV, Herskovic JR, Aphinyanaphongs Y, Aliferis CF, Sriram MG, Hersh WR. Using citation data to improve retrieval from MEDLINE. Journal of the American Medical Informatics Association. 2006 Jan-Feb;13(1):96-105. PMID: WOS:000234724000014. doi: 10.1197/jamia.M1909.

4. Bollen J, Van de Sompel H, Hagberg A, Chute R. A principal component analysis of 39 scientific impact measures. PLoS One. 2009 Jun 29;4(6):e6022. PMID: 19562078. doi: 10.1371/journal.pone.0006022.

5. Chen L, Holsapple CW, Hsiao SH, Ke ZH, Oh JY, Yang ZG. Knowledge-Dissemination Channels: Analytics of Stature Evaluation. Journal of the Association for Information Science and Technology. 2017 Apr;68(4):911-30. PMID: WOS:000396413900008. doi: 10.1002/asi.23725.

6. Chinchilla-Rodríguez Z, Benavent-Pérez M, de Moya-Anegón F, Miguel S. International collaboration in Medical Research in Latin America and the Caribbean (2003-2007). Journal of the American Society for Information Science and Technology. 2012;63(11):2223-38. PMID: 104431437. Language: English. Entry Date: 20121031. Revision Date: 20150820. Publication Type: Journal Article. doi: 10.1002/asi.22669.

7. Coughlin DM, Jansen BJ. Modeling journal bibliometrics to predict downloads and inform purchase decisions at university research libraries. Journal of the Association for Information Science and Technology. 2016 Sep;67(9):2263-73. PMID: WOS:000383602000015. doi: 10.1002/asi.23549.

8. De Souza CG, Barbastefano RG. Knowledge diffusion and collaboration networks on life cycle assessment. 2011.

9. Ebadi A, Schiffauerova A. On the Relation between the Small World Structure and Scientific Activities. Plos One. 2015 Mar 17;10(3). PMID: WOS:000351284600196. doi: ARTN e0121129

10.1371/journal.pone.0121129.

10. Stringer MJ, Sales-Pardo M, Amaral LAN. Effectiveness of Journal Ranking Schemes as a Tool for Locating Information. Plos One. 2008 Feb 27;3(2). PMID: WOS:000260586500027. doi: ARTN e1683

10.1371/journal.pone.0001683.

11. Uddin S, Hossain L, Rasmussen K. Network effects on scientific collaborations. PLoS One. 2013;8(2):e57546. PMID: 23469021. doi: 10.1371/journal.pone.0057546.

12. West JD, Jensen MC, Dandrea RJ, Gordon GJ, Bergstrom CT. Author-level Eigenfactor metrics: Evaluating the influence of authors, institutions, and countries within the social science research network community. Journal of the American Society for Information Science and Technology. 2013 Apr;64(4):787-801. PMID: WOS:000323386300009. doi: 10.1002/asi.22790.

13. Wren JD. Open access and openly accessible: a study of scientific publications shared via the internet. BMJ. 2005 May 14;330(7500):1128. PMID: 15827063. doi: 10.1136/bmj.38422.611736.E0.

14. Xu C, Ma BJ, Chen XH, Ma FC. Social tagging in the scholarly world. Journal of the American Society for Information Science and Technology. 2013 Oct;64(10):2045-57. PMID: WOS:000324100000006. doi: 10.1002/asi.22903.

15. Zhao DZ, Strotmann A. Information science during the first decade of the web: an enriched author cocitation analysis. Journal of the American Society for Information Science and Technology. 2008 Apr;59(6):916-37. PMID: WOS:000254363800006. doi: 10.1002/asi.20799.

#### Academic impact (n=7)

1. Athanasiou T, Patel V, Garas G, Ashrafian H, Hull L, Sevdalis N, et al. Mentoring perception, scientific collaboration and research performance: is there a 'gender gap' in academic medicine? An Academic Health Science Centre perspective. 2016.

2. Braun S. Supporting Research Impact Metrics in Academic Libraries: A Case Study. Portal-Libraries and the Academy. 2017 Jan;17(1):111-27. PMID: WOS:000396354300008. doi: 10.1353/pla.2017.0007.

3. Contandriopoulos D, Duhoux A, Larouche C, Perroux M. The Impact of a Researcher's Structural Position on Scientific Performance: An Empirical Analysis. PLoS One. 2016;11(8):e0161281. PMID: 27579954. doi: 10.1371/journal.pone.0161281.

4. Digiampietri LA, Mena-Chalco JP, Vaz de Melo PO, Malheiro AP, Meira DN, Franco LF, et al. BraX-Ray: an X-ray of the Brazilian computer science graduate programs. PLoS One. 2014;9(4):e94541. PMID: 24728179. doi: 10.1371/journal.pone.0094541.

5. Petr CG, Harrington D, Kim K, Black B, Cunningham-Williams RM, Bentley KJ. Quality Indicators and Expected Outcomes for Social Work PhD Programs: Perceptions of Social Work Students, Faculty, and Administrators. Journal of Social Work Education. 2015 Oct 2;51(4):648-67. PMID: WOS:000362649900004. doi: 10.1080/10437797.2015.1076272.

6. Ravenscroft J, Liakata M, Clare A, Duma D. Measuring scientific impact beyond academia: An assessment of existing impact metrics and proposed improvements. PLoS One. 2017;12(3):e0173152. PMID: 28278243. doi: 10.1371/journal.pone.0173152.

7. Talja S, Vakkari P, Fry J, Wouters P. Impact of research cultures on the use of digital library resources. Journal of the American Society for Information Science and Technology. 2007 Sep;58(11):1674-85. PMID: WOS:000249135100013. doi: 10.1002/asi.20650.

### Health research, but not discussing social media for dissemination (n=116)

#### Not mentioning social media nor research impact (n=17)

1. Balkanyi L, Schulz S, Cornet R, Bodenreider O. Medical concept representation: the years beyond 2000. Stud Health Technol Inform. 2013;192:1011. PMID: 23920785. doi: 10.3233/978-1-61499-289-9-1011.

2. Brookes PS. Internet publicity of data problems in the bioscience literature correlates with enhanced corrective action. PeerJ. 2014;2:e313. PMID: 24765564. doi: 10.7717/peerj.313.

3. Goerke K, Parke M, Horn J, Meyer C, Dormire K, White B, et al. Are results from randomized trials in anesthesiology robust or fragile? An analysis using the fragility index. Int J Evid Based Healthc. 2019 Aug 13;13:13. PMID: 31415254. doi: 10.1097/XEB.0000000000000200.

4. Pearce I. Editorial. 2015.

5. Petrin P, Baggio E, Spisni R, Rulli F. Use of virtual reality simulator in the training of postgraduated surgical residents. 2006.

6. Petrou S, Rivero-Arias O, Dakin H, Longworth L, Oppe M, Froud R, et al. Preferred reporting items for studies mapping onto preference-based outcome measures: the MAPS statement. Qual Life Res. 2016 Feb;25(2):275-81. PMID: 26231589. doi: 10.1007/s11136-015-1082-8.

7. Petrovcic R. Students' opinions on working in rural practice in Slovenia. Family Medicine and Primary Care Review. 2016;18(4):448-54. PMID: WOS:000392736900008. doi: 10.5114/fmpcr.2016.63700.

8. Peugh JL, Heck RH. Conducting Three-Level Longitudinal Analyses. Journal of Early Adolescence. 2017 Jan;37(1):7-58. PMID: WOS:000390573200002. doi: 10.1177/0272431616642329.

9. Peykari N, Tehrani FR, Malekafzali H, Hashemi Z, Djalalinia S. An Experience of Peer Education Model among Medical Science University Students in Iran. Iranian Journal of Public Health. 2011;40(1):57-62. PMID: WOS:000289127400007.

10. Phadtare A, Bahmani A, Shah A, Pietrobon R. Scientific writing: a randomized controlled trial comparing standard and on-line instruction. BMC Med Educ. 2009 May 27;9:27. PMID: 19473511. doi: 10.1186/1472-6920-9-27.

11. Phair RD. Why and How to Expand the Role of Systems Biology in Pharmaceutical Research and Development. Advances in Systems Biology. 2012;736:533-42. PMID: WOS:000300257900031. doi: 10.1007/978-1-4419-7210-1_31.

12. Prorok JC, Iserman EC, Wilczynski NL, Haynes RB. The quality, breadth, and timeliness of content updating vary substantially for 10 online medical texts: an analytic survey. J Clin Epidemiol. 2012 Dec;65(12):1289-95. PMID: 22974495. doi: 10.1016/j.jclinepi.2012.05.003.

13. Quint B. Truth: A Comeback Plan. Information Today. 2017;34(3):15-. PMID: 122530653. Language: English. Entry Date: 20170419. Revision Date: 20170419. Publication Type: Article. Journal Subset: Computer/Information Science.

14. Sidalak D, Purdy E, Luckett-Gatopoulos S, Murray H, Thoma B, Chan TM. Coached Peer Review: Developing the Next Generation of Authors. Acad Med. 2017 Feb;92(2):201-4. PMID: 27191842. doi: 10.1097/ACM.0000000000001224.

15. Tilson HA. Letter from the Editor- in-Chief. Environmental Health Perspectives. 2014 Apr;122(4):A88-A. PMID: WOS:000334069100001. doi: 10.1289/ehp.1408430.

16. Weissman RS. Letter from the Editor. 2016.

17. Wilson D. Editorial. Nursing Praxis in New Zealand. 2011;27(3):2-3. PMID: 104620901. Language: English. Entry Date: 20120619. Revision Date: 20150711. Publication Type: Journal Article.

#### Content analyses of health topics (n=7)

1. Chew C, Eysenbach G. Pandemics in the age of Twitter: content analysis of Tweets during the 2009 H1N1 outbreak. PLoS One. 2010 Nov 29;5(11):e14118. PMID: 21124761. doi: 10.1371/journal.pone.0014118.

2. Could MS, Midle JB, Insel B, Kleinman M. Suicide reporting content analysis: abstract development and reliability. 2007.

3. Delir Haghighi P, Kang YB, Buchbinder R, Burstein F, Whittle S. Investigating Subjective Experience and the Influence of Weather Among Individuals With Fibromyalgia: A Content Analysis of Twitter. 2017.

4. Hawkins JB, Brownstein JS, Tuli G, Runels T, Broecker K, Nsoesie EO, et al. Measuring patient-perceived quality of care in US hospitals using Twitter. Bmj Quality & Safety. 2016 Jun;25(6):404-13. PMID: WOS:000376924400005. doi: 10.1136/bmjqs-2015-004309.

5. Neilson C. How do Health Libraries Use Twitter? A Content Analysis. Journal of the Canadian Health Libraries Association (JCHLA). 2015;36(2):86-. PMID: 108975092. Language: English. Entry Date: 20151013. Revision Date: 20151013. Publication Type: Article.

6. Rees T, Adie E, Smith S. Bad news travels furthest: the social media impact of publications around trial disclosure and medical writing. Current Medical Research and Opinion. 2014 Apr;30:S10-S. PMID: WOS:000334146500010.

7. Wilkinson C, Bray L, Keating P, Carter B. Analysis of parent videos from the #notanurse-but campaign. 2017.

#### Social media for knowledge translation/education (n=24)

1. Belden D. Harnessing social networks to connect with audiences: if you build it, will they come 2.0? Internet Reference Services Quarterly. 2008;13(1):99-111. PMID: 105745548. Language: English. Entry Date: 20080620. Revision Date: 20150819. Publication Type: Journal Article.

2. Bender JL, O'Grady LA, Deshpande A, Cortinois AA, Saffie L, Husereau D, et al. Collaborative authoring: a case study of the use of a wiki as a tool to keep systematic reviews up to date. 2011.

3. Clark T, De Waard A, Herman I, Hovy E. The Future of Research Communication. Dagstuhl Rep. 2011;1(8):29-52. PMID: 26317061. doi: 10.4230/DagRep.1.8.29.

4. Das S, Girard L, Green T, Weitzman L, Lewis-Bowen A, Clark T. Building biomedical web communities using a semantically aware content management system. Briefings in Bioinformatics. 2009 Mar;10(2):129-38. PMID: WOS:000264388500003. doi: 10.1093/bib/bbn052.

5. de Carvalho EC, Batilana AP, Simkins J, Martins H, Shah J, Rajgor D, et al. Application description and policy model in collaborative environment for sharing of information on epidemiological and clinical research data sets. PLoS One. 2010 Feb 19;5(2):e9314. PMID: 20174560. doi: 10.1371/journal.pone.0009314.

6. Dong CY, Cheema M, Samarasekera D, Rajaratnam V. Using LinkedIn for Continuing Community of Practice Among Hand Surgeons Worldwide. Journal of Continuing Education in the Health Professions. 2015 Sum;35(3):185-91. PMID: WOS:000363878600005. doi: 10.1002/chp.21300.

7. Elzaabalawy SI, Abdelbaki MA, Abdelhakim AI, Alamir WM, Elsayed MO, Eryan MM, et al. Alternatives outreach and a new student movement for humane veterinary education and practice in Egypt. 2011.

8. Graziano M. Promoting Parkinson's specific physiotherapy practice: Association of Physiotherapists in Parkinson's Disease Europe (APPDE). Movement Disorders. 2013 Jun;28:S106-S. PMID: WOS:000320940501029.

9. Hall G, Feliz J. Spreading the word: Using podcasting to advance scientific knowledge across the spectrum of PM&R. 2016.

10. Hilton L. Social media's impact in dermatology. Dermatology Times. 2016;37(1):71-4. PMID: 112297947. Language: English. Entry Date: 20160119. Revision Date: 20160119. Publication Type: Article. Journal Subset: Biomedical.

11. Kahlon M, Yuan L, Daigre J, Meeks E, Nelson K, Piontkowski C, et al. The use and significance of a research networking system. J Med Internet Res. 2014 Feb 7;16(2):e46. PMID: 24509520. doi: 10.2196/jmir.3137.

12. Kapp JM, Hensel B, Schnoring KT. Is Twitter a forum for disseminating research to health policy makers? Ann Epidemiol. 2015 Dec;25(12):883-7. PMID: 26460202. doi: 10.1016/j.annepidem.2015.09.002.

13. Lamb LC, DiFiori MM, Jayaraman V, Shames BD, Feeney JM. Gamified Twitter Microblogging to Support Resident Preparation for the American Board of Surgery In-Service Training Examination. J Surg Educ. 2017 Nov - Dec;74(6):986-91. PMID: 28545826. doi: 10.1016/j.jsurg.2017.05.010.

14. Lei SF, Iles A, Kelly M. Characterizing the Networks of Digital Information that Support Collaborative Adaptive Forest Management in Sierra Nevada Forests. Environmental Management. 2015 Jul;56(1):94-109. PMID: WOS:000355621100008. doi: 10.1007/s00267-015-0497-x.

15. Mistry V. Critical care training: using Twitter as a teaching tool. British Journal of Nursing. 2011;20(20):1292-6. PMID: 108216886. Language: English. Entry Date: 20120113. Revision Date: 20150820. Publication Type: Journal Article.

16. Nwosu AC, Monnery D, Reid VL, Chapman L. Use of podcast technology to facilitate education, communication and dissemination in palliative care: the development of the AmiPal podcast. 2016.

17. Reuter K, Ukpolo F, Ward E, Wilson ML, Angyan P. Trial Promoter: A Web-Based Tool for Boosting the Promotion of Clinical Research Through Social Media. Journal of Medical Internet Research. 2016 Jun;18(6). PMID: WOS:000388495200030. doi: ARTN e144

10.2196/jmir.4726.

18. Riesel J, Greenberg SLM, Holmer H, Meara JG. Utilizing Social Media to Disseminate Information and Engage Feedback: Experiences from the Lancet Commission on Global Surgery. Journal of the American College of Surgeons. 2014 Sep;219(3):S61-S. PMID: WOS:000342420900116. doi: DOI 10.1016/j.jamcollsurg.2014.07.139.

19. Roupret M, Misrai V. [Exponential use of social media in medicine: example of the interest of Twitter(©) in urology]. 2015.

20. Schomberg JP, Haimson OL, Hayes GR, Anton-Culver H. Supplementing Public Health Inspection via Social Media. PLoS One. 2016;11(3):e0152117. PMID: 27023681. doi: 10.1371/journal.pone.0152117.

21. Sternberg KM, Loeb SL, Canes D, Donnelly L, Tsai MH. The use of Twitter to facilitate sharing of clinical expertise in urology. Journal of the American Medical Informatics Association. 2018 2018;25(2):183-6. PMID: 127951886. Language: English. Entry Date: In Process. Revision Date: 20190402. Publication Type: journal article. Journal Subset: Blind Peer Reviewed.

22. Sugimoto CR, Thelwall M. Scholars on soap boxes: Science communication and dissemination in TED videos. Journal of the American Society for Information Science and Technology. 2013 Apr;64(4):663-74. PMID: WOS:000323386300002. doi: 10.1002/asi.22764.

23. Teruya SA, Bazargan-Hejazi S. Social Media and Mentoring in Biomedical Research Faculty Development. J Fac Dev. 2014 Sep;28(3):13-22. PMID: 26120494.

24. Thoma B, Sanders JL, Lin M, Paterson QS, Steeg J, Chan TM. The social media index: measuring the impact of emergency medicine and critical care websites. West J Emerg Med. 2015 Mar;16(2):242-9. PMID: 25834664. doi: 10.5811/westjem.2015.1.24860.

#### Social media as sources of data (n=15)

1. Chiu K, Wagner L, Choe L, Chew C, Kremzner M. Piloting social engagement on a federal agency-administered Facebook page. J Am Pharm Assoc (2003). 2016 May-Jun;56(3):330-7. PMID: 27079138. doi: 10.1016/j.japh.2016.01.008.

2. Connor E. Using Wiki technology to build a faculty publications database. Journal of Electronic Resources in Medical Libraries. 2007;4(4):11-25. PMID: 105717740. Language: English. Entry Date: 20080516. Revision Date: 20150819. Publication Type: Journal Article.

3. Cooke DT, West H, Conway L, Freeman-Daily J, Hendrickson D, David EA, et al. Social media can be used as a qualitative research tool in surgical patient-centered outcomes research. Journal of the American College of Surgeons. 2015 Oct;221(4):E29-E. PMID: WOS:000386899000066. doi: DOI 10.1016/j.jamcollsurg.2015.08.374.

4. Du L, Rachul C, Guo Z, Caulfield T. Gordie Howe's "Miraculous Treatment": Case Study of Twitter Users' Reactions to a Sport Celebrity's Stem Cell Treatment. 2016.

5. Hansen HM, Murphy J, Richards AK, Duke J, Allen JA. Methodological considerations in analyzing twitter data. 2013.

6. Katsuki T, Mackey TK, Cuomo R. Establishing a Link Between Prescription Drug Abuse and Illicit Online Pharmacies: Analysis of Twitter Data. Journal of Medical Internet Research. 2015 Dec;17(12). PMID: WOS:000366558800001. doi: ARTN e280

10.2196/jmir.5144.

7. Kitchen H, Willgoss TG, Meysner S, Trigg A, Dickinson S, Humphrey L. Something Old, Something Borrowed, Something New: A Direct Comparison of Three Qualitative Elicitation Methods. Value in Health. 2015 Nov;18(7):A718-A. PMID: WOS:000209861400217. doi: DOI 10.1016/j.jval.2015.09.2715.

8. Meaney S, Cussen L, Greene RA, O'Donoghue K. Reaction on Twitter to a Cluster of Perinatal Deaths: A Mixed Method Study. JMIR Public Health Surveill. 2016 Jul 27;2(2):e36. PMID: 27466002. doi: 10.2196/publichealth.5333.

9. Mills I, Koeller HB, Hoover N, Youngren K, Smith S. Social media activity for optimizing publication timing in accordance with drug approval. Current Medical Research and Opinion. 2014 Apr;30:S17-S8. PMID: WOS:000334146500032.

10. Pinho-Costa L, Yakubu K, Hoedebecke K, Laranjo L, Reichel CP, Colon-Gonzalez MD, et al. Healthcare hashtag index development: Identifying global impact in social media. J Biomed Inform. 2016 Oct;63:390-9. PMID: 27645323. doi: 10.1016/j.jbi.2016.09.010.

11. Ryan G, Sfar-Gandoura H. Disseminating research information through Facebook and Twitter (DRIFT): presenting an evidence-based framework. Nurse Res. 2018 May 2;2:02. PMID: 29717844. doi: 10.7748/nr.2018.e1562.

12. Swan M. Crowdsourced health research studies: an important emerging complement to clinical trials in the public health research ecosystem. J Med Internet Res. 2012 Mar 7;14(2):e46. PMID: 22397809. doi: 10.2196/jmir.1988.

13. Valdez RS, Guterbock TM, Thompson MJ, Reilly JD, Menefee HK, Bennici MS, et al. Beyond traditional advertisements: leveraging Facebook's social structures for research recruitment. J Med Internet Res. 2014 Oct 27;16(10):e243. PMID: 25348050. doi: 10.2196/jmir.3786.

14. Woo H, Cho Y, Shim E, Lee JK, Lee CG, Kim SH. Estimating Influenza Outbreaks Using Both Search Engine Query Data and Social Media Data in South Korea. J Med Internet Res. 2016 Jul 4;18(7):e177. PMID: 27377323. doi: 10.2196/jmir.4955.

15. Yang YT, Horneffer M, DiLisio N. Mining social media and web searches for disease detection. J Public Health Res. 2013 Apr 28;2(1):17-21. PMID: 25170475. doi: 10.4081/jphr.2013.e4.

#### Social networks related to health and related analyses (n=13)

1. Abdalla M, Light-McGroary K, Tong C, Freeman AM. Launching the New American College of Cardiology Research Network Advancing High-Value Collaborative Research via "Innovative Networking". Journal of the American College of Cardiology. 2015 Mar 17;65(10):1053-5. PMID: WOS:000350635600014. doi: 10.1016/j.jacc.2015.01.020.

2. Bader GD, Kofia V, Isserlin R, Buchan AMJ. Social Network: A Cytoscape app for visualizing co-authorship networks. 2015.

3. Bian J, Xie M, Topaloglu U, Hudson T, Eswaran H, Hogan W. Social network analysis of biomedical research collaboration networks in a CTSA institution. J Biomed Inform. 2014 Dec;52:130-40. PMID: 24560679. doi: 10.1016/j.jbi.2014.01.015.

4. de Paula Fonseca EFB, Zicker F. Dengue research networks: building evidence for policy and planning in Brazil. Health Res Policy Syst. 2016 Nov 8;14(1):80. PMID: 27825383. doi: 10.1186/s12961-016-0151-y.

5. Duan Q. Research on Influence of Word-of-Mouth on Otc Drugs Purchase Intention Based on Social Network. Basic & Clinical Pharmacology & Toxicology. 2016 Jun;118:20-. PMID: WOS:000379130500065.

6. Dunn AG, Westbrook JI. Interpreting social network metrics in healthcare organisations: a review and guide to validating small networks. Soc Sci Med. 2011 Apr;72(7):1064-8. PMID: 21371798. doi: 10.1016/j.socscimed.2011.01.029.

7. Feinberg ME, Riggs NR, Greenberg MT. Social networks and community prevention coalitions. J Prim Prev. 2005 Jul;26(4):279-98. PMID: 15995800. doi: 10.1007/s10935-005-5390-4.

8. Fonseca Bde P, Sampaio RB, Fonseca MV, Zicker F. Co-authorship network analysis in health research: method and potential use. Health Res Policy Syst. 2016 Apr 30;14(1):34. PMID: 27138279. doi: 10.1186/s12961-016-0104-5.

9. Goncalves L, Souza KM. Evaluation of Dissemination of Brazilian Network for Health Technology Assessment (REBRATS). Value Health. 2014 Nov;17(7):A554. PMID: 27201813. doi: 10.1016/j.jval.2014.08.1815.

10. Heap G, Stones N. Networking to deliver research: Research radiographers within the Academic Clinical Oncology and Radiobiology Research Network (ACORRN). 2009.

11. Hofbauer S, Kalogirou C, Roghmann F, Seitz AK, Vallo S, Wezel F, et al. Modern networks: Topics in the working group "Bladder cancer research" of the GeSRU Academics. [German]. 2017.

12. Javadinia SA, Erfanian M, Abedini M, Bijari B. The Effects of Social Networks on Academic Achievement of Students, a Study in Birjand University of Medical Sciences. Iranian Journal of Medical Education. 2012;12(8):598-606. PMID: 104190408. Language: Persian. Entry Date: 20130712. Revision Date: 20150711. Publication Type: Journal Article.

13. Valente TW, Palinkas LA, Czaja S, Chu KH, Brown CH. Social network analysis for program implementation. PLoS One. 2015;10(6):e0131712. PMID: 26110842. doi: 10.1371/journal.pone.0131712.

#### Social media use among health professionals and health institutions (n=31)

1. Alotaibi NM, Badhiwala JH, Nassiri F, Guha D, Ibrahim GM, Shamji MF, et al. The Current Use of Social Media in Neurosurgery. World Neurosurgery. 2016 Apr;88:619-U90. PMID: WOS:000374649700081. doi: 10.1016/j.wneu.2015.11.011.

2. Alotaibi NM, Samuel N, Guha D, Nassiri F, Badhiwala JH, Tam J, et al. Social Media for Academic Neurosurgical Programs: The University of Toronto Experience. World Neurosurg. 2016 Sep;93:449-57. PMID: 27423202. doi: 10.1016/j.wneu.2016.06.134.

3. Alraies MC, Raza S, Ryan J. Twitter as a New Core Competency for Cardiologists. Circulation. 2018 Sep 25;138(13):1287-9. PMID: WOS:000452157200008. doi: 10.1161/Circulationaha.118.032999.

4. Alsobayel H. Use of Social Media for Professional Development by Health Care Professionals: A Cross-Sectional Web-Based Survey. 2016.

5. Benetoli A, Chen TF, Schaefer M, Chaar BB, Aslani P. Professional Use of Social Media by Pharmacists: A Qualitative Study. Journal of Medical Internet Research. 2016 Sep;18(9). PMID: WOS:000388495800013. doi: ARTN e258

10.2196/jmir.5702.

6. Bui D, Twibill K, Ee D, Mah D, Aleemullah T, Luk S, et al. Social Media and UNSW Sports Medicine Society: A new face in Sports and Exercise Medicine. 2015.

7. Chikramane A, Venkatraman L, Khanna V, Sinha S. Data sharing trends and integrity in the era of open science: a qualitative analysis of journals in top-ranked therapeutic areas. Current Medical Research and Opinion. 2017 May;33:9-. PMID: WOS:000400586700013.

8. Ciprut S, Curnyn C, Davuluri M, Sternberg K, Loeb S. Twitter Activity Associated With U.S. News and World Report Reputation Scores for Urology Departments. Urology. 2017 Oct;108(1):11-6. PMID: 28669746. doi: 10.1016/j.urology.2017.05.051.

9. Cochran A, Kao LS, Gusani NJ, Suliburk JW, Nwomeh BC. Use of Twitter to document the 2013 Academic Surgical Congress. Journal of Surgical Research. 2014 Jul;190(1):36-40. PMID: WOS:000338444700006. doi: 10.1016/j.jss.2014.02.029.

10. Collings S, Huxley L, Haworth D, Rashid W, Cairns A, Thompson G. Digital evolution of medical journals in 2013. Current Medical Research and Opinion. 2014 Apr;30:S13-S. PMID: WOS:000334146500019.

11. Desai T, Shariff A, Shariff A, Kats M, Fang XM, Christiano C, et al. Tweeting the Meeting: An In-Depth Analysis of Twitter Activity at Kidney Week 2011. Plos One. 2012 Jul 5;7(7). PMID: WOS:000306436300040. doi: ARTN e40253

10.1371/journal.pone.0040253.

12. Devine DA. Assessment of Nurse Faculty's Acceptance and Intent to Use Social Media Using the Unified Theory of Acceptance and Use of Technology 2 Model: Villanova University; 2015.

13. Dickson T, Le T, Sutch S, Woolley KL. Congress and journal practices in a digital and patient-centric era-hasten slowly? 2017.

14. Djuricich AM, Zee-Cheng JE. Live tweeting in medicine: 'Tweeting the meeting'. International Review of Psychiatry. 2015 Apr;27(2):133-9. PMID: WOS:000353525000007. doi: 10.3109/09540261.2014.1000270.

15. Ferguson C, Inglis SC, Newton PJ, Cripps PJS, Macdonald PS, Davidson PM. Social media: A tool to spread information: A case study analysis of Twitter conversation at the Cardiac Society of Australia a New Zealand 61st Annual Scientific Meeting 2013. Collegian. 2014;21(2):89-93. PMID: WOS:000337995300003. doi: 10.1016/j.colegn.2014.03.002.

16. Fernandez-Aleman JL, Sanchez Garcia AB, Lopez Montesinos MJ, Marques-Sanchez P, Bayon Darkistade E, Perez Rivera FJ. Exploring the Use of information and communication technologies and social networks among university nursing faculty staff. An opinion survey. 2014.

17. Finch C, Khan K, Verhagen E, Marino N. Symposium. 2012.

18. Gill BC, Ericson KJ, Hemal S, Babbar P, D AS. The Digital Footprint of Academic Urologists: Where Do we Stand? 2016.

19. Go PH, Klaassen Z, Chamberlain RS. Attitudes and Practices of Surgery Residency Program Directors Toward the Use of Social Networking Profiles to Select Residency Candidates: A Nationwide Survey Analysis. Journal of Surgical Education. 2012 May-Jun;69(3):292-300. PMID: WOS:000303074600005. doi: 10.1016/j.jsurg.2011.11.008.

20. Grande D, Gollust SE, Pany M, Seymour J, Goss A, Kilaru A, et al. Translating Research For Health Policy: Researchers' Perceptions And Use Of Social Media. Health Affairs. 2014 Jul;33(7):1278-85. PMID: WOS:000340469700024. doi: 10.1377/hlthaff.2014.0300.

21. Halboub E, Othathi F, Mutawwam F, Madkhali S, Somaili D, Alahmar N. Effect of social networking on academic achievement of dental students, Jazan University, Saudi Arabia. Eastern Mediterranean Health Journal. 2016;22(12):865-71. PMID: WOS:000399470900003. doi: Doi 10.26719/2016.22.12.865.

22. Hudson H, Hall J. Value of social media in reaching and engaging employers in Total Worker Health. J Occup Environ Med. 2013 Dec;55(12 Suppl):S78-81. PMID: 24284750. doi: 10.1097/JOM.0000000000000035.

23. Joshi ND, Lieber B, Wong K, Al-Alam E, Agarwal N, Diaz V. Social Media in Neurosurgery: Using ResearchGate. World Neurosurg. 2019 Jul;127:e950-e6. PMID: 30965167. doi: 10.1016/j.wneu.2019.04.007.

24. Koo K, Gormley EA. Shared Discussion or Self-Promotion? Use of Twitter by Us Urology Residency Programs. Journal of Urology. 2016 Apr;195(4):E220-E1. PMID: WOS:000375278600494. doi: DOI 10.1016/j.juro.2016.02.2788.

25. Maclure K, Brown A, Addison B, Pederson S, Stewart D. Social media: a force for inclusion. International Journal of Clinical Pharmacy. 2017 Feb;39(1):319-. PMID: WOS:000394437100304.

26. Martinez-Millana A, Fernandez-Llatas C, Basagoiti Bilbao I, Traver Salcedo M, Traver Salcedo V. Evaluating the Social Media Performance of Hospitals in Spain: A Longitudinal and Comparative Study. J Med Internet Res. 2017 May 23;19(5):e181. PMID: 28536091. doi: 10.2196/jmir.6763.

27. Mishori R, Singh LO, Levy B, Newport C. Mapping Physician Twitter Networks: Describing How They Work as a First Step in Understanding Connectivity, Information Flow, and Message Diffusion. Journal of Medical Internet Research. 2014 Apr;16(4):157-67. PMID: WOS:000336501600013. doi: ARTN e107

10.2196/jmir.3006.

28. Nair V, Khan S, Jhaveri KD. Interactive journals and the future of medical publications. Am J Med. 2012 Oct;125(10):1038-42. PMID: 22998882. doi: 10.1016/j.amjmed.2012.06.012.

29. Pearson D, Bond MC, Kegg J, Pillow T, Hopson L, Cooney R, et al. Evaluation of Social Media Use by Emergency Medicine Residents and Faculty. Western Journal of Emergency Medicine. 2015 Sep;16(5):715-20. PMID: WOS:000373117900018. doi: 10.5811/westjem.2015.7.26128.

30. Prabhu V, Rosenkrantz AB. Enriched Audience Engagement Through Twitter: Should More Academic Radiology Departments Seize the Opportunity? J Am Coll Radiol. 2015 Jul;12(7):756-9. PMID: 25979145. doi: 10.1016/j.jacr.2015.02.016.

31. Schleyer T, Spallek H, Butler BS, Subramanian S, Weiss D, Poythress ML, et al. Facebook for Scientists: Requirements and Services for Optimizing How Scientific Collaborations Are Established. Journal of Medical Internet Research. 2008;10(3). PMID: WOS:000263213500005. doi: ARTN e24

10.2196/jmir.1047.

#### Scoping, systematic reviews on social media in health topics (n=9)

1. Capurro D, Cole K, Echavarria MI, Joe J, Neogi T, Turner AM. The use of social networking sites for public health practice and research: a systematic review. J Med Internet Res. 2014 Mar 14;16(3):e79. PMID: 24642014. doi: 10.2196/jmir.2679.

2. Catala-Lopez F, Alonso-Arroyo A, Hutton B, Aleixandre-Benavent R, Moher D. Global collaborative networks on meta-analyses of randomized trials published in high impact factor medical journals: a social network analysis. BMC Med. 2014 Jan 29;12(1):15. PMID: 24476131. doi: 10.1186/1741-7015-12-15.

3. Colorafi K. Connected health: a review of the literature. Mhealth. 2016;2:13. PMID: 28293591. doi: 10.21037/mhealth.2016.03.09.

4. Holeman I, Cookson TP, Pagliari C. Digital technology for health sector governance in low and middle income countries: a scoping review. J Glob Health. 2016 Dec;6(2):020408. PMID: 27648255. doi: 10.7189/jogh.06.020408.

5. Madden K, Evaniew N, Scott T, Domazetoska E, Dosanjh P, Li CS, et al. Knowledge Dissemination of Intimate Partner Violence Intervention Studies Measured Using Alternative Metrics: Results From a Scoping Review. 2016 Jul 04.

6. Park BK, Calamaro C. A systematic review of social networking sites: innovative platforms for health research targeting adolescents and young adults. J Nurs Scholarsh. 2013 Sep;45(3):256-64. PMID: 23676115. doi: 10.1111/jnu.12032.

7. Sugimoto CR, Work S, Lariviere V, Haustein S. Scholarly Use of Social Media and Altmetrics: A Review of the Literature. Journal of the Association for Information Science and Technology. 2017 Sep;68(9):2037-62. PMID: WOS:000407793000001. doi: 10.1002/asi.23833.

8. Turk N. The impact of open access on the medical literature: A review of current literature. [Slovene]. 2016.

9. Wilson P, Petticrew M, Booth A. After the gold rush? A systematic and critical review of general medical podcasts. J R Soc Med. 2009 Feb;102(2):69-74. PMID: 19208871. doi: 10.1258/jrsm.2008.080245.

### Not original research articles (n=105)

#### Editorials/News articles on Altmetrics and social media (n=38)

1. Serials report. Serials Librarian. 2008;55(4):508-46. PMID: 105602431. Language: English. Entry Date: 20090410. Revision Date: 20150711. Publication Type: Journal Article.

2. Poster sessions. Serials Librarian. 2008;54(3-4):289-96. PMID: 105777337. Language: English. Entry Date: 20080801. Revision Date: 20150711. Publication Type: Journal Article.

3. What's new? Selected abstracts from JASIST. Bulletin of the American Society for Information Science & Technology. 2009;35(4):49-50. PMID: 105365082. Language: English. Entry Date: 20090807. Revision Date: 20150711. Publication Type: Journal Article.

4. INDUSTRY news. Online Searcher. 2013;37(6):6-9. PMID: 107938299. Language: English. Entry Date: 20131119. Revision Date: 20150712. Publication Type: Journal Article. Journal Subset: Computer/Information Science.

5. NEWS BYTES. Information Today. 2013;30(7):10-3. PMID: 107960226. Language: English. Entry Date: 20130724. Revision Date: 20150712. Publication Type: Journal Article.

6. Society News. The American Journal of Sports Medicine. 2013;41(9):2203-4. PMID: 104220337. Language: English. Entry Date: 20130905. Revision Date: 20150711. Publication Type: Journal Article. Journal Subset: Allied Health. doi: 10.1177/0363546513503447.

7. Wiley trials alternative metrics on subscription and open access articles. Information World Review. 2013:10-. PMID: 104169807. Language: English. Entry Date: 20131206. Revision Date: 20150711. Publication Type: Journal Article.

8. Alternative impact assessment trialled. CILIP Update. 2014:20-. PMID: 107885292. Language: English. Entry Date: 20140206. Revision Date: 20150712. Publication Type: Journal Article. Journal Subset: Computer/Information Science.

9. Current awareness and professional literature. Libraries for Nursing Bulletin. 2014 Winter2014/2015;34(3/4):93-5. PMID: 107773463. Language: English. Entry Date: 20150219. Revision Date: 20150712. Publication Type: Journal Article. Supplement Title: Winter2014/2015. Journal Subset: Computer/Information Science.

10. INDUSTRY news. Online Searcher. 2014;38(4):6-9. PMID: 103984803. Language: English. Entry Date: 20140805. Revision Date: 20150710. Publication Type: Journal Article. Journal Subset: Computer/Information Science.

11. NEWSBYTES. Information Today. 2014;31(3):12-5. PMID: 107899641. Language: English. Entry Date: 20140403. Revision Date: 20150712. Publication Type: Journal Article. Journal Subset: Computer/Information Science.

12. Altmetric offers insight for research news site. CILIP Update. 2015:22-. PMID: 112241625. Language: English. Entry Date: 20160211. Revision Date: 20160211. Publication Type: Article. Supplement Title: Dec2015/Jan2016. Journal Subset: Computer/Information Science.

13. INDUSTRY news. Online Searcher. 2015;39(4):6-9. PMID: 109819017. Language: English. Entry Date: 20150724. Revision Date: 20150923. Publication Type: Journal Article. Journal Subset: Computer/Information Science.

14. NEWSBYTES. Information Today. 2015;32(7):3-. PMID: 109416553. Language: English. Entry Date: 20150915. Revision Date: 20150916. Publication Type: Article. Journal Subset: Computer/Information Science.

15. Notice to Readers:MMWRReports Now Feature Altmetric Scores. MMWR Morbidity and Mortality Weekly Report. 2016;65(14):384-. PMID: 115165243. Language: English. Entry Date: In Process. Revision Date: 20160507. Publication Type: journal article. Journal Subset: Biomedical. doi: 10.15585/mmwr.mm6514a7.

16. INDUSTRY news. Online Searcher. 2016;40(6):6-9. PMID: 119486787. Language: English. Entry Date: 20161123. Revision Date: 20170313. Publication Type: Article. Journal Subset: Computer/Information Science.

17. NEWS BYTES. Information Today. 2017;34(3):3-. PMID: 122530643. Language: English. Entry Date: 20170419. Revision Date: 20170419. Publication Type: Article. Journal Subset: Computer/Information Science.

18. Anonymous. 10th Annual Meeting of the International Society for Medical Publication Professionals, ISMPP 2014. 2014.

19. Butt CA. Untitled: Tayo Adenaike. JAMA. 2016 Jul 19;316(3):252-3. PMID: 27434430. doi: 10.1001/jama.2015.14382.

20. Cabrera D, Vartabedian BS, Spinner RJ, Jordan BL, Aase LA, Timimi FK. More Than Likes and Tweets: Creating Social Media Portfolios for Academic Promotion and Tenure. Journal of graduate medical education. 2017 2017;9(4):421-5. PMID: rayyan-22796004.

21. Cawcutt K. Twitter Me This-Can Social Media Revolutionize Academic Medicine? Infect Control Hosp Epidemiol. 2017 Dec;38(12):1501-2. PMID: 29210345. doi: 10.1017/ice.2017.242.

22. Coyle JT. Changes in JAMA and the Archives of General Psychiatry: what they mean for readers and authors. Arch Gen Psychiatry. 2012 May;69(5):447-8. PMID: 22566577. doi: 10.1001/archgenpsychiatry.2012.398.

23. Crotty D. Altmetrics. Eur Heart J. 2017 Sep 14;38(35):2647-8. PMID: 28934843. doi: 10.1093/eurheartj/ehx447.

24. DeBord LC, Patel V, Braun TL, Dao H, Jr. Social media in dermatology: clinical relevance, academic value, and trends across platforms. J Dermatolog Treat. 2019 Aug;30(5):511-8. PMID: 30265614. doi: 10.1080/09546634.2018.1530444.

25. Fleischmann KR, Worrall A, Anderson TD, Goggins S, Burnett G. SIG CON Research Symposium: [Insert Title Here: Make Sure to Satisfy Titular Colonicity]. Bulletin of the Association for Information Science & Technology. 2016;42(3):33-5. PMID: 113307817. Language: English. Entry Date: 20170330. Revision Date: 20170330. Publication Type: Article. Journal Subset: Computer/Information Science. doi: 10.1002/bul2.2016.1720420311.

26. Gasparyan AY, Yessirkepov M, Voronov AA, Koroleva AM, Kitas GD. Comprehensive Approach to Open Access Publishing: Platforms and Tools. J Korean Med Sci. 2019 Jul 15;34(27):e184. PMID: 31293109. doi: 10.3346/jkms.2019.34.e184.

27. Gorodeski EZ, Rich MW, Hauptman PJ. Social Media and the Journal: Entering a New Era. J Card Fail. 2018 Jul;24(7):415-6. PMID: 30122230. doi: 10.1016/j.cardfail.2018.06.005.

28. Haustein S. SIG/MET: METRICS 2015: Workshop on Informetric and Scientometric Research. Bulletin of the Association for Information Science & Technology. 2016;42(3):24-7. PMID: 113307807. Language: English. Entry Date: 20170330. Revision Date: 20170330. Publication Type: Article. Journal Subset: Computer/Information Science. doi: 10.1002/bul2.2016.1720420308.

29. Heinemann MK. Alt(ernative) Metrics. Thorac Cardiovasc Surg. 2018 Sep;66(6):425. PMID: 30180266. doi: 10.1055/s-0038-1669912.

30. O'Neill J. The Past and Future at ARCS. Information Today. 2015;32(5):10-1. PMID: 109811241. Language: English. Entry Date: 20150707. Revision Date: 20150923. Publication Type: Journal Article.

31. Philbrick JL. Measuring Research Impact on the Social Web: A Look at Altmetrics. MLA News. 2014;54(2):13-. PMID: 107888664. Language: English. Entry Date: 20140221. Revision Date: 20150712. Publication Type: Journal Article. Journal Subset: Computer/Information Science.

32. Pike GH. Elsevier Buys SSRN: What It Means for Scholarly Publication. Information Today. 2016;33(6):1-29. PMID: 116878875. Language: English. Entry Date: 20160720. Revision Date: 20160721. Publication Type: Article. Journal Subset: Computer/Information Science.

33. Prabhu VC, Swong K, Hendler G. Publication Metrics in Neurosurgery. World Neurosurg. 2017 Sep;105:993-6. PMID: 28645594. doi: 10.1016/j.wneu.2017.06.081.

34. Riss P, Dwyer PL. The 2012 IUJ impact factor: Another step forward? 2013.

35. Serruys PW, Onuma Y. Twitterature: will social media have an impact on scientific journals? EuroIntervention. 2018 Oct 12;14(9):e962-e4. PMID: 30307395. doi: 10.4244/EIJV14I9A171.

36. Sharma N, Doherty I. Twitter for doctors - promising educational occurrences. Educ Prim Care. 2018 Jan;29(1):56-7. PMID: 28795642. doi: 10.1080/14739879.2017.1362670.

37. Susarla SM, Friedrich JB. Discussion: Alternative Metrics of Scholarly Output: The Relationship among Altmetric Score, Mendeley Reader Score, Citations, and Downloads in Plastic and Reconstructive Surgery. Plast Reconstr Surg. 2018 Mar;141(3):810-1. PMID: 29481414. doi: 10.1097/PRS.0000000000004133.

38. Tregoning J. How will you judge me if not by impact factor? Nature. 2018 Jun;558(7710):345. PMID: 29921857. doi: 10.1038/d41586-018-05467-5.

#### Editorials and opinion articles about Altmetrics in health research literature (n=31)

1. Abaci A. Scientific competition, impact factor, and Altmetrics. Anatol J Cardiol. 2017 Nov;18(5):313. PMID: 29145234. doi: 10.14744/AnatolJCardiol.2017.11.

2. Akers KG. Introducing altmetrics to the Journal of the Medical Library Association. J Med Libr Assoc. 2017 Jul;105(3):213-5. PMID: 28670207. doi: 10.5195/jmla.2017.250.

3. Araujo A, Costa a, Nascimento DP, Gonzalez GZ, Costa LOP. How to increase the visibility of scientific articles through social media? Brazilian Journal of Physical Therapy / Revista Brasileira de Fisioterapia. 2018 2018;22(6):435-6. PMID: 132991844. Language: English. Entry Date: 20181116. Revision Date: 20181119. Publication Type: Editorial. Journal Subset: Allied Health.

4. Baheti AD, Bhargava P. Altmetrics: A Measure of Social Attention toward Scientific Research. Curr Probl Diagn Radiol. 2017 Nov - Dec;46(6):391-2. PMID: 28751106. doi: 10.1067/j.cpradiol.2017.06.005.

5. Bonsaksen T. #IncreaseImpact: Added value for your published article. British Journal of Occupational Therapy. 2019 2019;82(6):323-5. PMID: 136876502. Language: English. Entry Date: 20190613. Revision Date: 20190628. Publication Type: Editorial. Journal Subset: Allied Health.

6. Boschert S. Blognosis: Outside views can shape inside scoop at NEJM. 2012.

7. Cardona-Grau D. Commentary on "The effect of social media (#SoMe) on journal impact factor and parental awareness in paediatric urology". 2017 May 20.

8. Carrasco G, Lorenzo S. Social networks and impact factor of medical journals. [Spanish]. 2012.

9. Chavda J, Patel A. Measuring research impact: bibliometrics, social media, altmetrics, and the BJGP. Br J Gen Pract. 2016 Jan;66(642):e59-61. PMID: 26719483. doi: 10.3399/bjgp16X683353.

10. Cress PE. Using altmetrics and social media to supplement impact factor: maximizing your article's academic and societal impact. Aesthet Surg J. 2014 Sep;34(7):1123-6. PMID: 25024453. doi: 10.1177/1090820X14542973.

11. Dixon A, Fitzgerald RT, Gaillard F. Letter by Dixon et al regarding article, "A randomized trial of social media from Circulation". Circulation. 2015 Mar 31;131(13):e393. PMID: 25825404. doi: 10.1161/CIRCULATIONAHA.114.014460.

12. Djuricich AM, Madanick RD. Letter by Djuricich and Madanick regarding article, "A randomized trial of social media from Circulation". Circulation. 2015 Mar 31;131(13):e395. PMID: 25825406. doi: 10.1161/CIRCULATIONAHA.115.015128.

13. Fazel S, Wolf A. What is the impact of a research publication? Evid Based Ment Health. 2017 May;20(2):33-4. PMID: 28385690. doi: 10.1136/eb-2017-102668.

14. Featherstone R. Scholarly Tweets: Measuring Research Impact via Altmetrics. Journal of the Canadian Health Libraries Association / Journal de l'Association des bibliothèques de la santé du Canada. 2014;35(2):60-3. PMID: 103885267. Language: English. Entry Date: 20140905. Revision Date: 20150819. Publication Type: Journal Article. Journal Subset: Canada. doi: 10.5596/c14-015.

15. Finch C. Embracing social media for research promotion and dissemination. 2012.

16. Fox CS, Bonaca MA, Ryan JJ, Massaro JM, Barry K, Loscalzo J. Response to letters regarding article, "A randomized trial of social media from Circulation". Circulation. 2015 Mar 31;131(13):e396. PMID: 25825407. doi: 10.1161/CIRCULATIONAHA.115.016083.

17. Karimkhani C, Gamble R, Dellavalle RP. Social media impact factor: the top ten dermatology journals on Facebook and Twitter. Dermatol Online J. 2014 Apr 16;20(4):22327. PMID: 24746295.

18. Otto CM. Heartbeat: Altmetrics and Cardiovascular Risk Factor Studies. Heart. 2015 Dec;101(24):1931-2. PMID: 26611857. doi: 10.1136/heartjnl-2015-308995.

19. Roberts J. Measuring the social media impact of your headache article. Headache. 2014 Oct;54(9):1435-6. PMID: 25324162. doi: 10.1111/head.12452.

20. Schnitzler K, Davies N, Ross F, Harris R. Using TwitterTM to drive research impact: A discussion of strategies, opportunities and challenges. 2016.

21. Scott C, Farrow P, Young F. Use of Twitter and other social media to disseminate specialist clinical research: analysis using Altmetric. Current Medical Research and Opinion. 2015 Apr;31:S18-S. PMID: WOS:000353315600041.

22. Semsarian C, Ingles J. Letter by Semsarian and Ingles regarding article, "A randomized trial of social media from Circulation". Circulation. 2015 Mar 31;131(13):e394. PMID: 25825405. doi: 10.1161/CIRCULATIONAHA.114.014628.

23. Serrano-Cobos J, Calduch-Losa Á, De Dios JG, Aleix, re-Benavent R. Scientific communication (XXXV). How to make a social media strategy for paediatricians (II). Visibility in social network. Acta Pediatrica Espanola. 2016 2016;74(7):e159-e66. PMID: rayyan-22797619.

24. Shekhawat KS, Chauhan A. Altmetrics: A new paradigm for scholarly communication. Indian J Dent Res. 2019 Jan-Feb;30(1):125-6. PMID: 30900670. doi: 10.4103/ijdr.IJDR_27_17.

25. Sinnenberg L, Buttenheim AM, Padrez K, Mancheno C, Ungar L, Merchant RM. Twitter as a Tool for Health Research: A Systematic Review. American Journal of Public Health. 2017 Jan;107(1):E1-E8. PMID: WOS:000396524500001. doi: 10.2105/Ajph.2016.303512.

26. Smith DR, Watson R. Career development tips for today's nursing academic: bibliometrics, altmetrics and social media. J Adv Nurs. 2016 Nov;72(11):2654-61. PMID: 27399604. doi: 10.1111/jan.13067.

27. Soreide K. Numbers needed to tweet: social media and impact on surgery. Eur J Surg Oncol. 2019 Feb;45(2):292-5. PMID: 30343997. doi: 10.1016/j.ejso.2018.10.054.

28. Thoma B, Cabrera D, Trueger NS. Letter by Thoma et al regarding article, "A randomized trial of social media from Circulation". Circulation. 2015 Mar 31;131(13):e392. PMID: 25825403. doi: 10.1161/CIRCULATIONAHA.114.014441.

29. Thoma B, Cabrera D, Trueger NS. Letter by Thoma et al Regarding Article, "A Randomized Trial of Social Media From Circulation". Circulation. 2015 Mar 31;131(13):E392-E. PMID: WOS:000351953300003. doi: 10.1161/Circulationaha.114.014441.

30. Truong M. Social Media: The New Impact Factor? J Minim Invasive Gynecol. 2016 Jul-Aug;23(5):655-7. PMID: 27282219. doi: 10.1016/j.jmig.2016.06.001.

31. Wijdicks EF. The Journal and Social Media. Neurocrit Care. 2017 Feb;26(1):1-2. PMID: 27966091. doi: 10.1007/s12028-016-0364-5.

#### Editorials and opinion articles about bibliometrics in health (n=15)

1. Aleixandre-Benavent R, Ferrer-Sapena A, Alonso-Arroyo A, Vidal-Lnfer A, Dominguez RL, Gonzalez De Dios J. Scientific communication (XXVI). How to increase the spread and impact of papers in Pediatrics through the open science. 2015.

2. Alfonso F. [The long pilgrimage of Spanish biomedical journals toward excellence. Who helps? Quality, impact and research merit]. Endocrinol Nutr. 2010 Mar;57(3):110-20. PMID: 20347618. doi: 10.1016/j.endonu.2010.02.003.

3. Aminpour F. How to improve webometric ranks of Iranian medical universities. [Persian]. 2011.

4. Chakravarthy M, Hetzer R, Delmo Walter EM. Ramification of indexing of medical journals. HSR Proc Intensive Care Cardiovasc Anesth. 2013;5(1):5-8. PMID: 23734283.

5. Divatia JV. The Indian Journal of Anaesthesia in 2017: Time to make an impact. Indian Journal of Anaesthesia. 2017 Jan;61(1):1-2. PMID: WOS:000394428400001. doi: 10.4103/0019-5049.198409.

6. Docherty AB, Klein AA. The fate of manuscripts rejected from Anaesthesia. Anaesthesia. 2017 Apr;72(4):427-30. PMID: 28168693. doi: 10.1111/anae.13829.

7. Ezema IJ, Onyancha OB. Citation impact of health and medical journals in Africa: does open accessibility matter? Electronic Library. 2017 2017;35(5):934-52. PMID: 125727522. Language: English. Entry Date: 20171024. Revision Date: 20171025. Publication Type: Article.

8. Farooqui MS. JCPSP - In pursuit of impact factor. 2005.

9. Hua F, Sun H, Walsh T, Worthington H, Glenny AM. Open access to journal articles in dentistry: Prevalence and citation impact. J Dent. 2016 Apr;47:41-8. PMID: 26875610. doi: 10.1016/j.jdent.2016.02.005.

10. Knowlton SE, Paganoni S, Niehaus W, Verduzco-Gutierrez M, Sharma R, Iaccarino MA, et al. Measuring the Impact of Research Using Conventional and Alternative Metrics. Am J Phys Med Rehabil. 2019 Apr;98(4):331-8. PMID: 30300231. doi: 10.1097/PHM.0000000000001066.

11. Naveed S, Waqas A, Majeed S, Zeshan M, Jahan N, Salman R. Evolution of child and adolescent psychiatry: A bibliometric analysis of the top seven journals of child and adolescent psychiatry. 2016.

12. Rhee JS. High-Impact Articles-Citations, Downloads, and Altmetric Score. JAMA Facial Plast Surg. 2015 Sep-Oct;17(5):323-4. PMID: 26226501. doi: 10.1001/jamafacial.2015.0869.

13. Smith DR, Hazelton M. Bibliometric awareness in nursing scholarship: can we afford to ignore it any longer? Nurs Health Sci. 2011 Dec;13(4):384-7. PMID: 22098385. doi: 10.1111/j.1442-2018.2011.00652.x.

14. Wells RG. Beyond the Impact Factor: Why CMGH? Cell Mol Gastroenterol Hepatol. 2015 Nov;1(6):571. PMID: 28210699. doi: 10.1016/j.jcmgh.2015.09.006.

15. Wijdicks EF. The journal and academia. Neurocrit Care. 2012 Feb;16(1):1-3. PMID: 22183702. doi: 10.1007/s12028-011-9660-2.

#### Editorials and opinion articles on social media for health professionals (n=21)

1. Allen HG, McAuley JH, Moseley GL. Missing in action? Dissemination via social media by pain-focussed and general medical journals. 2011.

2. Bacigalupe G. Is there a role for social technologies in collaborative healthcare? Fam Syst Health. 2011 Mar;29(1):1-14. PMID: 21417520. doi: 10.1037/a0022093.

3. Ban VS, Lega B, Batjer HH. Maximizing the Potential of Social Media and Social Networks in Neurosurgery. World Neurosurg. 2016 Jul;91:609-10. PMID: 26968446. doi: 10.1016/j.wneu.2016.02.117.

4. Batt-Rawden S, Flickinger T, Weiner J, Cheston C, Chisolm M. The role of social media in clinical excellence. Clin Teach. 2014 Jul;11(4):264-9. PMID: 24917094. doi: 10.1111/tct.12129.

5. Bell JM. Social Media and Family Nursing Scholars: Catching Up With 2007. J Fam Nurs. 2017 Feb;23(1):3-12. PMID: 28795932. doi: 10.1177/1074840717694524.

6. Brown S. Tap and talk. Therapy Today. 2017;28(4):8-11. PMID: 123052637. Language: English. Entry Date: 20170517. Revision Date: 20170517. Publication Type: Article. Journal Subset: Biomedical.

7. Childs J. Blogging as a tool to address communication challenges among nurses. 2015.

8. Kamel Boulos MN, Wheeler S. The emerging Web 2.0 social software: an enabling suite of sociable technologies in health and health care education. Health Info Libr J. 2007 Mar;24(1):2-23. PMID: 17331140. doi: 10.1111/j.1471-1842.2007.00701.x.

9. Leveridge MJ. The emerging role of social media in urology. Rev Urol. 2014;16(3):110-7. PMID: 25337040.

10. Navratil V, Navratil L. Expansive promotion of scientific research. 2011.

11. Patel D, Jermacane D. Social media in travel medicine: a review. Travel Med Infect Dis. 2015 Mar-Apr;13(2):135-42. PMID: 25817428. doi: 10.1016/j.tmaid.2015.03.006.

12. Peters ME, Uible E, Chisolm MS. A Twitter Education: Why Psychiatrists Should Tweet. Current Psychiatry Reports. 2015 Dec;17(12). PMID: WOS:000366807800002. doi: ARTN 94

10.1007/s11920-015-0635-4.

13. Piscotty R, Voepel-Lewis T, Lee SH, Annis-Emeott A, Lee E, Kalisch B. To tweet or not to tweet? Nurses, social media, and patient care. 2013.

14. Redsicker P. Top 3 social media metrics dermatologists should know. Dermatology Times. 2014;35(4):82-. PMID: 103885305. Language: English. Entry Date: 20140905. Revision Date: 20150710. Publication Type: Journal Article.

15. Rodriguez-Socarras ME, Gomez-Rivas J, Alvarez-Maestro M, Tortolero L, Ribal MJ, Sanz MG, et al. Spanish adaptation of the recommendations for the appropriate use of social networks in urology of the European Association of Urology. Actas Urologicas Espanolas. 2016 Sep;40(7):417-23. PMID: WOS:000381956100002. doi: 10.1016/j.acuro.2015.12.005.

16. Roupret M, Misrai V. Exponential use of social media in medicine: example of the interest of Twitter(©) in urology. [French]. 2015.

17. Sciortino JE. All about Connection: A Message from JOGC's New Managing Editor. Journal of Obstetrics and Gynaecology Canada. 2017 Mar;39(3):116-7. PMID: WOS:000443555600002. doi: 10.1016/j.jogc.2017.01.017.

18. Tippin S, Arnold L. Social networks: bringing a high-fidelity simulator to life on Facebook. Nurse Educ. 2012 Jul-Aug;37(4):148-9. PMID: 22688870. doi: 10.1097/NNE.0b013e31825a8770.

19. Youd J. Keep your wits in the Twittersphere. Emerg Nurse. 2017 Feb 10;24(9):17. PMID: 28185516. doi: 10.7748/en.24.9.17.s23.

20. Zavod RM. Establishing a presence in the digital age. 2012.

21. Zusman EE. Social Media in Academic Neurosurgery. World Neurosurg. 2016 Jul;91:606-8. PMID: 26996732. doi: 10.1016/j.wneu.2016.02.118.

### Health research articles on the topic, but not reporting all relevant outcomes (n=54)

#### Systematic reviews (n=3)

1. Davidson PM, Newton PJ, Ferguson C, Daly J, Elliott D, Homer C, et al. Rating and ranking the role of bibliometrics and webometrics in nursing and midwifery. ScientificWorldJournal. 2014;2014:135812. PMID: 24550691. doi: 10.1155/2014/135812.

2. Evaniew N, Adili AF, Ghert M, Khan M, Madden K, Smith C, et al. The Scholarly Influence of Orthopaedic Research According to Conventional and Alternative Metrics: A Systematic Review. JBJS Rev. 2017 May;5(5):e5. PMID: 28557819. doi: 10.2106/JBJS.RVW.16.00059.

3. Patthi B, Prasad M, Gupta R, Singla A, Kumar JK, Dhama K, et al. Altmetrics - A Collated Adjunct Beyond Citations for Scholarly Impact: A Systematic Review. J Clin Diagn Res. 2017 Jun;11(6):ZE16-ZE20. PMID: 28764311. doi: 10.7860/JCDR/2017/26153.10078.

#### Research reporting results on bibliometrics in health research (n=22)

1. Bazm S, Kalantar SM, Mirzaei M. Bibliometric mapping and clustering analysis of Iranian papers on reproductive medicine in Scopus database (2010-2014). Int J Reprod Biomed (Yazd). 2016 Jun;14(6):371-82. PMID: 27525320.

2. Bazrafshan A, Haghdoost AA, Zare M. A comparison of downloads, readership and citations data for the Journal of Medical Hypotheses and Ideas. Journal of Medical Hypotheses and Ideas. 2015 Mar;9(1):1-4. PMID: WOS:000218835700001. doi: 10.1016/j.jmhi.2014.06.001.

3. Bender ME, Edwards S, von Philipsborn P, Steinbeis F, Keil T, Tinnemann P. Using Co-authorship Networks to Map and Analyse Global Neglected Tropical Disease Research with an Affiliation to Germany. Plos Neglected Tropical Diseases. 2015 Dec;9(12). PMID: WOS:000368345100010. doi: ARTN e0004182

10.1371/journal.pntd.0004182.

4. Birks Y, Fairhurst C, Bloor K, Campbell M, Baird W, Torgerson D. Use of the h-index to measure the quality of the output of health services researchers. J Health Serv Res Policy. 2014 Apr;19(2):102-9. PMID: 24406401. doi: 10.1177/1355819613518766.

5. Boudry C. Web 2.0 applications in medicine: trends and topics in the literature. Med 2 0. 2015 Apr 1;4(1):e2. PMID: 25842175. doi: 10.2196/med20.3628.

6. Busch C, Taylor D, Salvadore M, Fransen C, Croce S, Patel N, et al. The leading edge: developing a multichannel approach measuring reach in medical publications. Current Medical Research and Opinion. 2015 Apr;31:S10-S. PMID: WOS:000353315600021.

7. Chen X, Lun Y, Yan J, Hao T, Weng H. Discovering thematic change and evolution of utilizing social media for healthcare research. BMC Med Inform Decis Mak. 2019 Apr 9;19(Suppl 2):50. PMID: 30961624. doi: 10.1186/s12911-019-0757-4.

8. Craig ID. The Journal of Sexual Medicine--impact factor predictions and analysis. J Sex Med. 2007 Jul;4(4 Pt 1):855-8. PMID: 17627734. doi: 10.1111/j.1743-6109.2007.00515.x.

9. Golubic R, Rudes M, Kovacic N, Marusic M, Marusic A. Calculating impact factor: How bibliographical classification of journal items affects the impact factor of large and small journals. Science and Engineering Ethics. 2008 Mar;14(1):41-9. PMID: WOS:000253683700004. doi: 10.1007/s11948-007-9044-3.

10. Hawkins CM, Hillman BJ, Carlos RC, Rawson JV, Haines R, Duszak R, Jr. The impact of social media on readership of a peer-reviewed medical journal. J Am Coll Radiol. 2014 Nov;11(11):1038-43. PMID: 25439618. doi: 10.1016/j.jacr.2014.07.029.

11. Hye Jeong K, Dae Young Y, Eun Soo K, Eun Joo Y, Hong Jun J, Jong Young L, et al. The most mentioned neurointervention articles in online media: a bibliometric analysis of the top 101 articles with the highest altmetric attention scores. Journal of NeuroInterventional Surgery. 2019 2019;11(5):528-32. PMID: 135955495. Language: English. Entry Date: 20190422. Revision Date: 20190423. Publication Type: Article. Journal Subset: Biomedical.

12. Jemielniak D, Masukume G, Wilamowski M. The Most Influential Medical Journals According to Wikipedia: Quantitative Analysis. Journal of Medical Internet Research. 2019 Jan 18;21(1):37-. PMID: WOS:000456631400001. doi: ARTN e11429

10.2196/11429.

13. Kim Y, Kim JE, Kim YH, Yoon DY, Kim YJ, Bae JS. Social attention and scientific articles on stroke: Altmetric analysis of top-50 articles. Clin Neurol Neurosurg. 2019 Aug;183:105386. PMID: 31207458. doi: 10.1016/j.clineuro.2019.105386.

14. Perchik J, Cohen H, Cobb W, Choudhri A. Citation analysis of pediatric radiology articles: 4 general radiology journals vs. pediatric radiology. 2017.

15. Ruano J, Aguilar-Luque M, Isla-Tejera B, Alcalde-Mellado P, Gay-Mimbrera J, Hernandez-Romero JL, et al. Relationships between abstract features and methodological quality explained variations of social media activity derived from systematic reviews about psoriasis interventions. J Clin Epidemiol. 2018 Sep;101:35-43. PMID: 29803759. doi: 10.1016/j.jclinepi.2018.05.015.

16. Thelwall M, Maflahi N. Guideline references and academic citations as evidence of the clinical value of health research. Journal of the Association for Information Science and Technology. 2016 Apr;67(4):960-6. PMID: WOS:000372926300015. doi: 10.1002/asi.23432.

17. Trueger NS, Bokarius AV, Carroll S, April MD, Thoma B. Impact of a Physician-Led Social Media Sharing Program on a Medical Journal's Web Traffic. J Am Coll Radiol. 2018 Jan;15(1 Pt B):184-9. PMID: 29122507. doi: 10.1016/j.jacr.2017.09.035.

18. Tyler Nix A, Smith JE. Research Impact Core: A Research Impact Initiative at the University of Michigan. Medical Reference Services Quarterly. 2019 2019;38(3):260-70. PMID: 137907081. Language: English. Entry Date: 20190808. Revision Date: 20190814. Publication Type: Article. Journal Subset: Computer/Information Science.

19. Wekerle C, Vakili N, Stewart SH, Black T. The utility of Twitter as a tool for increasing reach of research on sexual violence. Child Abuse Negl. 2018 Nov;85:220-8. PMID: 29778296. doi: 10.1016/j.chiabu.2018.04.019.

20. Yao Q, Lyu PH, Ma FC, Yao L, Zhang SJ. Global informetric perspective studies on translational medical research. BMC Med Inform Decis Mak. 2013 Jul 26;13:77. PMID: 23885955. doi: 10.1186/1472-6947-13-77.

21. Yiu SH, Dewhirst S, Lee C, Jalaili A, Frank JR. Do real-time Twitter metrics correlate with traditional emergency medicine post-conference speaker evaluations? 2016.

22. Yousefi A, Hemmat M, Gilvari A, Shahmirzadi T. Citation analysis and co-authorship of Iranian researchers in the field of immunology in ISI web of science: A brief report. [Persian]. 2012.

#### Research reporting results on Altmetrics or social media metrics (n=29)

1. Allen CG, Andersen B, Chambers DA, Groshek J, Roberts MC. Twitter use at the 2016 Conference on the Science of Dissemination and Implementation in Health: analyzing #DIScience16. Implement Sci. 2018 Feb 20;13(1):34. PMID: 29458394. doi: 10.1186/s13012-018-0723-z.

2. Chiang AL, Alakbarli J, Rabinowitz LG, Chan WW. Disparities in Social Media Exposure of Peer-Reviewed Manuscripts in Gastroenterology. Gastroenterology. 2016 Apr;150(4):S832-S. PMID: WOS:000391783500104. doi: Doi 10.1016/S0016-5085(16)32816-5.

3. Dellavalle RP, Pearson T, Tamai JM, Brewer AC, Henley J, Endly D, et al. Dermatology journal social media impact. Journal of Investigative Dermatology. 2012 Sep;132:S73-S. PMID: WOS:000307814000408.

4. Dyson MP, Newton AS, Shave K, Featherstone RM, Thomson D, Wingert A, et al. Social Media for the Dissemination of Cochrane Child Health Evidence: Evaluation Study. J Med Internet Res. 2017 Sep 1;19(9):e308. PMID: 28864427. doi: 10.2196/jmir.7819.

5. Fargen KM, Ducruet AF, Hyer M, Hirsch JA, Tarr RW. Expanding the social media presence of the Journal of Neurointerventional Surgery: editor's report. J Neurointerv Surg. 2017 Feb;9(2):215-8. PMID: 26927814. doi: 10.1136/neurintsurg-2015-012251.

6. Gamble JM, Traynor RL, Gruzd A, Mai P, Dormuth CR, Sketris IS. Measuring the impact of pharmacoepidemiologic research using altmetrics: A case study of a CNODES drug-safety article. Pharmacoepidemiol Drug Saf. 2018 Mar 24;24:24. PMID: 29575351. doi: 10.1002/pds.4401.

7. Gamble R, Gilchrest B, Dellavalle R. Trends in social media use among dermatology journals. Journal of Investigative Dermatology. 2013 May;133:S98-S. PMID: WOS:000317698900572.

8. Gates A, Featherstone R, Shave K, Scott SD, Hartling L. Dissemination of evidence in paediatric emergency medicine: a quantitative descriptive evaluation of a 16-week social media promotion. BMJ Open. 2018 Jun 6;8(6):e022298. PMID: 29880576. doi: 10.1136/bmjopen-2018-022298.

9. Haneef R, Ravaud P, Baron G, Ghosn L, Boutron I. Factors associated with online media attention to research: a cohort study of articles evaluating cancer treatments. Res Integr Peer Rev. 2017 2017;2:9. PMID: 29451556. doi: 10.1186/s41073-017-0033-z.

10. Hawkins CM, Hunter M, Kolenic GE, Carlos RC. Social Media and Peer-Reviewed Medical Journal Readership: A Randomized Prospective Controlled Trial. J Am Coll Radiol. 2017 May;14(5):596-602. PMID: 28268163. doi: 10.1016/j.jacr.2016.12.024.

11. Huang S, Martin LJ, Yeh CH, Chin A, Murray H, Sanderson WB, et al. The effect of an infographic promotion on research dissemination and readership: A randomized controlled trial. CJEM. 2018 Nov;20(6):826-33. PMID: 30289098. doi: 10.1017/cem.2018.436.

12. Karimkhani C, Gamble R, Dellavalle R. Looking for a journal with the most social media impact: JID tops facebook while JAMA Dermatology tops twitter. Journal of Investigative Dermatology. 2014 May;134:S59-S. PMID: WOS:000334560400342.

13. Kolahi J, Iranmanesh P, Khazaei S. Altmetric analysis of 2015 dental literature: a cross sectional survey. Br Dent J. 2017 May 12;222(9):695-9. PMID: 28496225. doi: 10.1038/sj.bdj.2017.408.

14. Kolahi J, Khazaei S. Altmetric: Top 50 dental articles in 2014. Br Dent J. 2016 Jun 10;220(11):569-74. PMID: 27283563. doi: 10.1038/sj.bdj.2016.411.

15. Kudlow P, Cockerill M, Toccalino D, Dziadyk DB, Rutledge A, Shachak A, et al. Online distribution channel increases article usage on Mendeley: a randomized controlled trial. Scientometrics. 2017 2017;112(3):1537-56. PMID: 28804178. doi: 10.1007/s11192-017-2438-3.

16. Lindquist LA, Ramirez-Zohfeld V. Visual Abstracts to Disseminate Geriatrics Research Through Social Media. Journal of the American Geriatrics Society. 2019 Jun;67(6):1128-31. PMID: WOS:000471133600007. doi: 10.1111/jgs.15853.

17. Maggio LA, Meyer HS, Artino AR. Beyond Citation Rates: A Real-Time Impact Analysis of Health Professions Education Research Using Altmetrics. Academic Medicine. 2017 Oct;92(10):1449-55. PMID: WOS:000411902800029. doi: 10.1097/Acm.0000000000001897.

18. Mills I, Gardner K, English M, Hoover N, Smith S, Youngren K. Social media usage by medical journals: implications for publication planning. Current Medical Research and Opinion. 2012 Apr;28:S15-S. PMID: WOS:000303248700030.

19. Niehaus WN, Silver JK, Katz MS. The PM&R Journal Implements a Social Media Strategy to Disseminate Research and Track Alternative Metrics in Physical Medicine and Rehabilitation. PM R. 2018 May;10(5):538-43. PMID: 29253533. doi: 10.1016/j.pmrj.2017.12.003.

20. Parker T, Boland G, Palmisano S, Miller A. Benchmarking Twitter usage among scientific journals. Current Medical Research and Opinion. 2012 Apr;28:S10-S. PMID: WOS:000303248700011.

21. Robinson-Garcia N, Costas R, Isett K, Melkers J, Hicks D. The unbearable emptiness of tweeting-About journal articles. PLoS One. 2017 2017;12(8):e0183551. PMID: 28837664. doi: 10.1371/journal.pone.0183551.

22. Sarwal A, Tran H, Kumar A, Wijdicks EF. Neurocritical care twitter journal club :Using social media to enhance academic engagement. Neurocritical Care. 2017 2017;27(2):S283. PMID: rayyan-22798029.

23. Skobe C, Mooney L, Kopf W, Rouhi S. Assessing industry-sponsored medical publications using alternative metrics. Current Medical Research and Opinion. 2016 2016;32:S5-S. PMID: WOS:000388103700005.

24. Thangasamy I, Woo H. International Urology Journal Club via a Social Media Microblogging Platform-An Online Pilot Project. 2013.

25. Thoma B, Chan TM, Kapur P, Sifford D, Siemens M, Paddock M, et al. The Social Media Index as an Indicator of Quality for Emergency Medicine Blogs: A METRIQ Study. Annals of Emergency Medicine. 2018 Dec;72(6):696-702. PMID: WOS:000450286400018. doi: 10.1016/j.annemergmed.2018.05.003.

26. Topf JM, Hiremath S. Social media, medicine and the modern journal club. Int Rev Psychiatry. 2015 Apr;27(2):147-54. PMID: 25906989. doi: 10.3109/09540261.2014.998991.

27. Wang J, Alotaibi NM, Ibrahim GM, Kulkarni AV, Lozano AM. The Spectrum of Altmetrics in Neurosurgery: The Top 100 "Trending" Articles in Neurosurgical Journals. World Neurosurg. 2017 Jul;103:883-95 e1. PMID: 28478251. doi: 10.1016/j.wneu.2017.04.157.

28. Wray CM, Auerbach AD, Arora VM. The Adoption of an Online Journal Club to Improve Research Dissemination and Social Media Engagement Among Hospitalists. J Hosp Med. 2018 Nov;13(11):764-9. PMID: 30484779. doi: 10.12788/jhm.2987.

29. Zhou JZ, Lemelman BT, Done N, Henderson ML, Macmillan A, Song DH, et al. Social Media and the Dissemination of Research: Insights from the Most Widely Circulated Articles in Plastic Surgery. Plast Reconstr Surg. 2018 Aug;142(2):555-61. PMID: 30045187. doi: 10.1097/PRS.0000000000004598.

### Health research discussing impact of social media for research dissemination but from the wrong study population (n=18)

#### Social media metrics and bibliometrics for journals (n=18)

1. Alotaibi NM, Guha D, Fallah A, Aldakkan A, Nassiri F, Badhiwala JH, et al. Social Media Metrics and Bibliometric Profiles of Neurosurgical Departments and Journals: Is There a Relationship? 2016.

2. Asyyed Z, McGuire C, Samargandi O, Al-Youha S, Williams JG. The Use of Twitter by Plastic Surgery Journals. Plast Reconstr Surg. 2019 May;143(5):1092e-8e. PMID: 31033839. doi: 10.1097/PRS.0000000000005535.

3. Cardona-Grau D, Sorokin I, Leinwand G, Welliver C. Introducing the Twitter Impact Factor: An Objective Measure of Urology's Academic Impact on Twitter. Eur Urol Focus. 2016 Oct;2(4):412-7. PMID: 28723474. doi: 10.1016/j.euf.2016.03.006.

4. Cosco TD. Medical journals, impact and social media: an ecological study of the Twittersphere. CMAJ. 2015 Dec 8;187(18):1353-7. PMID: 26644544. doi: 10.1503/cmaj.150976.

5. Duffy CC, Bass GA, Linton KN, Honan DM. Social media and anaesthesia journals. Oxford University Press / USA; 2015. p. 940-1.

6. Han J, Ziaeian B. Social Media Usage, Impact Factor, and Mean Altmetric Attention Scores: Characteristics and Correlates in Major Cardiology Journals. Journal of the American College of Cardiology. 2019 Mar 12;73(9):3027-. PMID: WOS:000460565903042. doi: Doi 10.1016/S0735-1097(19)33633-2.

7. Hughes H, Hughes A, Murphy C. The Use of Twitter by the Trauma and Orthopaedic Surgery Journals: Twitter Activity, Impact Factor, and Alternative Metrics. Cureus. 2017 Dec 10;9(12):e1931. PMID: 29464138. doi: 10.7759/cureus.1931.

8. Hughes H, Hughes A, Murphy CG. Correction: The Use of Twitter by Trauma and Orthopaedic Surgery Journals: Twitter Activity, Impact Factor, and Alternative Metrics. Cureus. 2018 Jun 15;10(6):c13. PMID: 29923551. doi: 10.7759/cureus.c13.

9. Kelly BS, Redmond CE, Nason GJ, Healy GM, Horgan NA, Heffernan EJ. The Use of Twitter by Radiology Journals: An Analysis of Twitter Activity and Impact Factor. J Am Coll Radiol. 2016 Nov;13(11):1391-6. PMID: 27577594. doi: 10.1016/j.jacr.2016.06.041.

10. Kolahi J, Khazaei S, Bidram E, Kelishadi R. Altmetric Analysis of Contemporary Iranian Medical Journals. Int J Prev Med. 2019 2019;10:112. PMID: 31360359. doi: 10.4103/ijpvm.IJPVM_134_19.

11. Lane P. Adoption of social media channels in leading medical journals in different therapeutic areasy. 2014.

12. McNamara P, Usher K. Share or perish: Social media and the International Journal of Mental Health Nursing. Int J Ment Health Nurs. 2019 Aug;28(4):960-70. PMID: 31257702. doi: 10.1111/inm.12600.

13. Nason GJ, O'Kelly F, Kelly ME, Phelan N, Manecksha RP, Lawrentschuk N, et al. The emerging use of Twitter by urological journals. Bju International. 2015 Mar;115(3):486-90. PMID: WOS:000350111100028. doi: 10.1111/bju.12840.

14. O'Kelly F, Nason GJ, Manecksha RP, Cascio S, Quinn FJ, Leonard M, et al. The effect of social media (#SoMe) on journal impact factor and parental awareness in paediatric urology. 2017 Apr 21.

15. Patel RR, Hill MK, Smith MK, Seeker P, Dellavalle RP. An updated assessment of social media usage by dermatology journals and organizations. Dermatol Online J. 2018 Feb 15;24(2):15. PMID: 29630149.

16. Scarlat MM, Mavrogenis AF, Pecina M, Niculescu M. Impact and alternative metrics for medical publishing: our experience with International Orthopaedics. International Orthopaedics. 2015 Aug;39(8):1459-64. PMID: WOS:000358385000001. doi: 10.1007/s00264-015-2766-y.

17. Shah K. Social Media Following and Cardiology Journal Impact Factor. Journal of the American College of Cardiology. 2017 Mar 21;69(11):2509-. PMID: WOS:000397342303431. doi: 10.1016/S0735-1097(17)35898-9.

18. Wong K, Piraquive J, Levi JR. Social media presence of otolaryngology journals: The past, present, and future. Laryngoscope. 2018 Feb;128(2):363-8. PMID: 28600839. doi: 10.1002/lary.26727.

### Other reasons (n=30)

#### Duplicate records (n=26)

1. Alotaibi NM, Badhiwala JH, Nassiri F, Guha D, Ibrahim GM, Shamji MF, et al. The Current Use of Social Media in Neurosurgery. World Neurosurgery. 2016 Apr;88:619-U90. PMID: WOS:000374649700081. doi: 10.1016/j.wneu.2015.11.011.

2. Baan CC, Dor FJMF. The Transplantation Journal on Social Media: The @TransplantJrnl Journey From Impact Factor to Klout Score. Transplantation. 2017 Jan;101(1):8-10. PMID: WOS:000391957500014. doi: 10.1097/Tp.0000000000001581.

3. Bonaca MA, Ryan JJ, Massaro JM, Barry K, Loscalzo J. A randomized trial of social media from Circulation. 2015.

4. Bonaca MA, Ryan JJ, Massaro JM, Barry K, Loscalzo J. A randomized trial of social media from Circulation. 2015.

5. Boulos MNK, Wheeler S. The emerging Web 2.0 social software: an enabling suite of sociable technologies in health and health care education. Health Information and Libraries Journal. 2007 Mar;24(1):2-23. PMID: WOS:000244613900002. doi: DOI 10.1111/j.1471-1842.2007.00701.x.

6. Carrasco G, Lorenzo S. [Social networks and impact factor of medical journals]. Rev Calid Asist. 2012 Sep-Oct;27(5):247-8. PMID: 22999006. doi: 10.1016/j.cali.2012.08.001.

7. Di Girolamo N, Reynders RM. Health care articles with simple and declarative titles were more likely to be in the Altmetric Top 100. 2016.

8. Duffy CC, Bass GA, Linton KN, Honan DM. Social media and anaesthesia journals. Br J Anaesth. 2015 Dec;115(6):940-1. PMID: 26582860. doi: 10.1093/bja/aev389.

9. Goncalves L, Souza KM, Elias FTS. Evaluation of Dissemination of Brazilian Network for Health Technology Assessment (Rebrats). Value in Health. 2013 Nov;16(7):A582-A3. PMID: WOS:000326247602201. doi: DOI 10.1016/j.jval.2013.08.1599.

10. Hall N. The Kardashian index: A measure of discrepant social media profile for scientists. Genome Biol. 2014 Jul 30;15(7):424. PMID: 25315513. doi: 10.1186/s13059-014-0424-0.

11. Haustein S, Costas R, Lariviere V. Characterizing social media metrics of scholarly papers: The effect of document properties and collaboration patterns. 2015.

12. Kim AE, Hansen HM, Murphy J, Richards AK, Duke J, Allen JA. Methodological considerations in analyzing Twitter data. J Natl Cancer Inst Monogr. 2013 Dec;2013(47):140-6. PMID: 24395983. doi: 10.1093/jncimonographs/lgt026.

13. Petrou S, Rivero-Arias O, Dakin H, Longworth L, Oppe M, Froud R, et al. Preferred reporting items for studies mapping onto preference-based outcome measures: The MAPS statement. J Med Econ. 2015;18(11):851-7. PMID: 26295698. doi: 10.3111/13696998.2015.1070554.

14. Quintana DS, Doan NT. Twitter Article Mentions and Citations: An Exploratory Analysis of Publications in the American Journal of Psychiatry. Arlington, Virginia: American Psychiatric Publishing, Inc.; 2016. p. 194-.

15. Rees T, Adie E, Smith S. Bad news travels furthest: the social media impact of publications around trial disclosure and medical writing. Current Medical Research and Opinion. 2014 Apr;30:S10-S. PMID: WOS:000334146500010.

16. Rees T, Adie E, Smith S. Bad news travels furthest: the social media impact of publications around trial disclosure and medical writing. Current Medical Research and Opinion. 2014 Apr;30:S10-S. PMID: WOS:000334146500010.

17. Rosenkrantz AB, Ayoola A, Singh K, Duszak R, Jr. Alternative Metrics ("Altmetrics") for Assessing Article Impact in Popular General Radiology Journals. Acad Radiol. 2017 Jul;24(7):891-7. PMID: 28256440. doi: 10.1016/j.acra.2016.11.019.

18. Scarlat MM, Mavrogenis AF, Pecina M, Niculescu M. Impact and alternative metrics for medical publishing: our experience with International Orthopaedics. Int Orthop. 2015 Aug;39(8):1459-64. PMID: 25947897. doi: 10.1007/s00264-015-2766-y.

19. Schnitzler K, Davies N, Ross F, Harris R. Using Twitter to drive research impact: A discussion of strategies, opportunities and challenges. Int J Nurs Stud. 2016 Jul;59:15-26. PMID: 27222446. doi: 10.1016/j.ijnurstu.2016.02.004.

20. Scotti V. Bibliometrics and web use: The birth of altmetrics. [Italian]. 2015.

21. Scotti V, De Silvestri A, Scudeller L, Abele P, Topuz F, Curti M. Novel bibliometric scores for evaluating research quality and output: a correlation study with established indexes. Int J Biol Markers. 2016 Dec 23;31(4):e451-e5. PMID: 27312588. doi: 10.5301/jbm.5000217.

22. Tonia T, Van Oyen H, Berger A, Schindler C, Kunzli N. If I tweet will you cite? The effect of social media exposure of articles on downloads and citations. Int J Public Health. 2016 May;61(4):513-20. PMID: 27193574. doi: 10.1007/s00038-016-0831-y.

23. Trueger NS, Thoma B, Hsu CH, Sullivan D, Peters L, Lin M. The Altmetric Score: A New Measure for Article-Level Dissemination and Impact. Annals of Emergency Medicine. 2015 Nov;66(5):549-53. PMID: WOS:000364621900022. doi: 10.1016/j.annemergmed.2015.04.022.

24. Trueger NS, Thoma B, Hsu CH, Sullivan D, Peters L, Lin M. The Altmetric Score: A New Measure for Article-Level Dissemination and Impact. Ann Emerg Med. 2015 Nov;66(5):549-53. PMID: 26004769. doi: 10.1016/j.annemergmed.2015.04.022.

25. Wilson P, Petticrew M, Booth A. After the gold rush? A systematic and critical review of general medical podcasts. J R Soc Med. 2009 Feb;102(2):69-74. PMID: 19208871. doi: 10.1258/jrsm.2008.080245.

26. Wise J. Promoting research on social media has little impact. 2014.

#### Citations only, full-text not available (n=4)

1. Negoita AG. Valorization of surgical research: Objectives and strategies. 2016.

2. Sketris IS, Gruzd A, Mai P, Gamble JM, Dormuth C. Analyzing the Short-Term Social Media Impact of a Drug Safety Publication-A Case Study Approach. Pharmacoepidemiology and Drug Safety. 2014 Oct;23:120-. PMID: WOS:000342763600223.

3. Thoma B, Sanders JL, Paterson Q, Steeg JB, Cadogan M, Lin M. The social media index: Curating emergency medicine blogs and podcasts. 2014.

4. Tykhonkova IO. How to become famous and popular in science (All scientometric tricks in 5 minutes for everyone). 2015.
