# Supplementary material for: The Use of Social Media to Increase the Impact of Health Research: Systematic Review
Source: J Med Internet Res. 2020 Jul 6;22(7):e15607. doi: 10.2196/15607 (PMC7380994; doi:10.2196/15607)
Supplement: Multimedia Appendix 3 [file jmir_v22i7e15607_app3.docx]

## Multimedia Appendix 3: Detailed characteristics of included studies

### Studies assessing the impact of social media interventions (n=7)

| **Author, year [reference number]**  **Type of study** | **Report characteristics** | **Intervention and control groups** | **Metrics and outcomes assessed and reported** | **Results**^[[1]](#footnote-1)^ | **Funding, Conflicts of interest and Notes^1^** |
| --- | --- | --- | --- | --- | --- |
| Allen, 2013  Quasi-experimental (before-after) | - Health Area: Clinical pain sciences - Journal: PLOS ONE - Population/Unit of analysis: 16 original research articles Sources of data: PLOS ONE, interest to the readership of bodyinmind.org blog. - Period of analysis: 1/1/2006 – 31/12/2011 | - No control group (n = 16) - Social media intervention: blog posts about the research articles further shared on Facebook, Twitter, LinkedIn and ResearchBlogging.org; - The message: around 500 words long, tag line directing the reader to the on-line version of the article. All posts were released on a Tuesday (between 6 and 7 am) or between 11 pm Thursday and 2 am Friday, Australian Eastern Summertime - Profile owner: 4 researchers in PLOS ONE (bodyinmind.org: average 2585 unique views/week. Each post has viewed a mean (SD) of 507 (160) times in the week following publication. In the 28 days after publication, a mean (SD) of 693 (135) unique visitors saw the post in their Facebook newsfeed; 35 (16) unique visitors clicked on each post; 6 (4) unique visitors) - Duration: 18 weeks - Intensity of intervention: post shared on all four outlets on the day of blog broadcast - Incentive/Paid promotion: not reported | - Social media: blog post (views); Facebook statistics and twitter comments and re-tweets. Reach, virality, and engagement. - Bibliometrics: Citation count (Scopus), nine months post-intervention and 1-2 years after the publication of the original research articles   Outcomes:   - 7-day rate of HTML views and PDF downloads - Facebook statistics (insights) - Twitter comments and re-tweets - Reach (number of unique visitors) engagement, and virality | - There was a main effect of Week on HTML views (F(1,15) = 6.27, *P* = .02) [calculated effect size: Cohen’s d = 1.25 (95% CI =0.18 – 2.23]. The rate of HTML views was also higher either side of the social media release than it was either side of the control date - The rate of PDF downloads was higher during the second week than during the first (main effect of Week – F(1,15) = 10.83, *P* = .01)” [calculated effect size: Cohen’s d = 1.65 (95% CI =0.51 – 2.78]. […] “higher either side of the social media release than it was either side of the control Date (main effect of date – F(1,15) = 6.57, *P* = .02)” (p. 4) [calculated effect size: Cohen’s d = 1.28 (95% CI =0.21 – 2.36]. - Engagement was 5.3% of reach and virality was 0.9% of engagement. None of the social media metrics related to the increase in rate of HTML views of the research article (*P* = .95 for reach; *P* = .81 for engagement; *P* = 0.54 for virality), nor to the increase in PDF downloads of the research article (*P* = .32 for reach; *P* = .86 for engagement; *P* = .93 for virality). - There was no relationship between citations on Scopus about one year after publication and any of the social media metrics (*P* = .68 for all). | - Funding: Canadian Institutes of Health Research Postdoctoral Training Fellowship, the National Health & Medical Research Council Research, Australian Government Postgraduate award. - Declared COI: "This experiment was conducted using www.bodyinmind.org. Web metrics of this website are used as evidence of the authors' research social media reach. The authors, therefore, stand to benefit indirectly should this manuscript increase the reach of that website. This does not alter the authors' adherence to all the PLOS ONE policies on sharing data and materials." |
| Cawcutt 2019  Quasi-experimental (before-after) | - Health Area: Women’s health - Journal: Various journals - Population/Unit of analysis: 8 articles - Period of analysis: April 10, 2018 (20 days before and 50 days after the chat) | - No control group - Social media intervention: n=8 articles tweeted and chatted during a Physician’s Weekly (PW) tweet chat event (#PWChat) - The message: one question per article were posted on Twitter - Profile owner: one of the authors and PW tweet account - Duration: 15 hours - Intensity of the intervention: post shared | - Social media: Twitter impressions, engagements, link clicks, likes, retweets. - Altmetric attention score, rank, counts - Bibliometrics: Article downloads | - 1500 tweets, replies, and retweets from 294 contributors, which resulted in 8.6 million impressions, potentially reaching 569785 Twitter users. - Altmetric Attention Score of each article increased (average, 126.5 points; range, 91–208 points). Within the respective journal, the Altmetric Rank of seven articles improved (range, 3 to ‡19), while the eighth maintained its #1 rank. The one article for which share and download data were available experienced a 729% increase in shares following prechat posts and another 113% bump after the chat, a 1667% increase overall (n=45–795). - Similarly, downloads, and presumably reads, increased 712% following prechat posts and another 47% bump after the chat, a 1093% increase overall (n = 394–4700) | - Funding: None reported. - Declared COI: No competing financial interests exist |
| Fox, 2015  Experimental (RCT) | - Health Area: Cardiology - Journal: Circulation - Population/Unit of analysis: 286 articles appearing in the journal (excluding those promoted through AHA press releases and author profiles: n=243) - Source of data: Journal Circulation - Period of analysis: 10/9/2013-12/10/2014 | - n= 243 (121 intervention, 122 control) - Social media intervention: Twitter and Facebook posts through the official Circulation of social media accounts. The posts provided a toll-free link to the full-text version of the article. - The message: a key figure was included in conveying the main point of the article written in simple English - Profile owner: Circulation (number of followers: 16,215 Facebook followers, 2,219 Twitter followers at the trial start; 28,177 Facebook followers, 4,759 Twitter followers by the trial end) - Duration: 52 weeks - Intensity of intervention: One post for each study report appeared online between Monday and Thursday of the weekly journal cycle - Incentive/ Paid promotion: Toll-free link to the full-text version of the article. | - Social media metrics: Twitter and Facebook metrics; unique 30-day article page views (inclusive of all HTML and pdf downloads and abstract views; through Circulation’s Google analytics account – note: article page views are an indicator used in Altmetrics. - Bibliometrics: Not reported (assumption: online page views have previously been shown to correlate with subsequent article citations).   Outcome:   - Unique 30-day article page views | - No difference in median 30-day page views between 2 groups (*P* = .80) | - Funding: Not reported - Declared COI: Not reported |
| Fox, 2016  Experimental (RCT) | - Health Area: Cardiology - Journal: Circulation - Population/Unit of analysis: 192 original articles appearing on the journal; excluding those promoted through AHA press releases and author profiles: n=152) - Source of data: Journal Circulation - Period of analysis: 13/1/2015 – 22/9/2015 | - n= 152 (74 intervention, 78 control) - Social media intervention: Twitter and Facebook - The Message: written in clear English, included a key figure or image -if possible posts contained a toll-free link to the full-text version. - Profile owner: Circulation (number of followers: 46,431 Facebook likes, 6,746 Twitter followers at the trial start; 87,607 Facebook likes, 10,072 Twitter followers by the trial end) - Duration: 34 weeks - Intensity of intervention: 2-7 posts on both Facebook and Twitter daily; all posts reposted the next day at 11 AM, 3 PM, up to a total of 3 posts. On Twitter, posts retweeted by official Circulation Twitter account. - Incentive/ Paid promotion: toll-free link to article full-text and Facebook posts were boosted for 24 hours for a total of $10 for each post using a targeted approach to individuals interested in cardiology. | - Social media metrics: unique 30-day page views (including abstract, HTML, and PDF page views; through Circulation’s Google Analytics account) – note: article page views are an indicator used in Altmetrics; 7-day page views of manuscripts boosted with Facebook advertisement versus those on Facebook without ads. - Bibliometrics: Not reported (assumption: online page views have previously been shown to correlate with subsequent article citations).   Outcomes:   - Unique 30-day article page views - Unique 7-day page views | - 30-day page views: no difference between 2 groups (*P* = .38) - 7-day page views: no difference between 2 groups (*P* = .17) | - Funding: Not reported - Declared COI: “Fox, Ryan, Bonaca, Barry, Loscalzo, and Massaro are editors at Circulation and report receiving compensation for this work from the American Heart Association. Caroline Fox became an employee of Merck Research Labs as of December 14, 2015, and reports earning income and holding stock options.'' - Notes: the trial was stopped early due to futility - Augmented Fox 2015, using a higher intensity of social media, larger followings, more postings, and boosting (promoting) posts |
| Hoang, 2015  Quasi-experimental (retrospective cohort) | - Health area: Radiology - Journal: Journal of the American College of Radiology - Population/Unit of analysis: 2 research articles appearing on the American Journal of Neuroradiology (AJNR) and the American Journal of Roentgenology (AJR) and discussed in Radiopedia.org’s blog - Sources of data: Radiopaedia.org; AJNR, AJR - Period of analysis: 4/2013-9/2014 | - n=2 articles (no control) - Social media intervention: Blog posts on Radiopedia.org; podcast shared on Twitter and Facebook - The Message: blogging about the content of the journal articles - Profile owner: - Duration: not specified - Intensity of intervention: Not clearly indicated, but initial Tweet on September 2013, then podcast in January 2014; blog post in Novemeber 2013, shared on social media in February and August 2014 - Incentive/ Paid promotion: Not paid | - Social media metrics: number of monthly page views on Blogpost (online reach) - Bibliometrics: Number of HTML page views, PDF downloads on journal websites | - Above-average increase in page views activity in August 2014 and September 2014, which coincided with Radiopaedia.org social media promotion. - No increased activity was seen beyond the publication date - AJR podcast in January did not correspond with an above-average increase in AJNR article page views for that month | - Funding: Not reported - Declared COI: Not reported |
| Thoma, 2018  Experimental (RCT) | - Health area: Emergency Medicine - Journal: Canadian Journal of Emergency Medicine - Population/Unit of analysis: 29 articles selected for intervention and control - Period of analysis: July-November 2016 | - n=29 (11 infographic intervention, 6, podcast intervention, 12 control) - Social media intervention: podcast or infographic or “standard social media promotion”. All articles included in the study received standard SoMe promotion. This included a tweet from the journal’s Twitter account (@CJEMonline) and a post on the CJEM Facebook page containing a link to the article and a brief (1- to 2-sentence) summary; Infographics were published as part of a collaboration between CJEM and CanadiEM. The infographic and article were also promoted on the CanadiEM and CJEM Twitter and Facebook accounts. odcasts were published as part of a collaboration between CJEM and the SGEM podcast (www.thesgem. com). The SGEM is a nonprofit Canadian website that critically appraises new and recent research using downloadable podcasts linked with online content. - Profile owner: CJEM (Number of followers: not reported) - Duration: 20 weeks - Intensity of intervention: 4 articles per issue ad hoc promotion (intensity not specified). - Incentive/ Paid promotion: not reported | - Social media: Twitter and Facebook impressions, engagements, link clicks, likes, retweets. - Altmetric attention score, - Bibliometrics: Article abstract and full-text views downloads | - Increased abstract readership - Podcast and infographic strategies significantly increased article Altmetric scores and abstract readership, they did not significantly increase full-text readership. | - Funding: Not reported - Declared COI: None declared |
| Tonia, 2016  Experimental (RCT) | - Health area: Public health - Journal: International Journal of Public Health (IJPH) - Population/Unit of analysis: 133 original research articles published in the journal (excluded: reviews, brief reports, editorials, and commentaries); analyzed 130 - Source of data: International Journal of Public Health; Springer citations; Web of Science - Period of analysis 12/2012-12/2014 (citations up to 31/4/2015) | - n= 130 (65 intervention and 65 control) - Social media intervention: When papers in the experimental group were published online, they received exposure in the IJPH blog, Twitter, and Facebook accounts: a short post presented the paper in the blog, and a Twitter and Facebook update summarized the main message of the paper and provided a link to it. Two weeks after the first intervention, the Tweet was repeated (second intervention). Ten weeks after the second intervention, IJPH posted about the article on Facebook and tweeted again. One of the authors (TT) posted all the notifications between Monday and Friday. All three interventions were made on the same day of the week, and time of day, for each paper. Automatic posting on FB for 3rd intervention, and 2nd and 3rd exposure on twitter - The message: Posts were written in plain language and highlighted the main point of the paper. Whenever possible, paper authors were ‘‘tagged’’ if they had Twitter accounts. - Profile owner: IJPH (Number of followers: not reported) - Duration: 12 weeks - Intensity of intervention: 3 times: first post (baseline); second post (2 weeks after baseline); third post (12 weeks after baseline). - Incentive/ Paid promotion: not reported | - Social media metrics: IJPH blog, Twitter, Facebook accounts - Bibliometrics: paper downloads from the publisher (Springer); and citations using ISI Web of Science   Outcomes:   - Number of full-text article downloads - Number of article citations from online publications up to March 31, 2015 | - There were 25,641 downloads: 12,466 in the SM exposure group, and 13,175 in the control group. The mean number of downloads per paper was 191.8 (SD 156.1, median 133.00, range 54–910) for the SM exposure group and 202.7 (SD 181.1, median 147.00, range 53–1035) for the control group. The number of downloads did not differ significantly between groups (*P* = .60) - During the follow-up period for the 130 manuscripts, there were a total of 105 citations; 55 in the exposure group, and 50 in the control group. No significant difference between the mean number of citations per paper between the two groups (*P* = .88). - The number of downloads and the number of citations significantly correlated for all papers (r = .52, *P* < .01); with the correlation being stronger in the intervention group (r = .67, *P* < .01). - All papers: significantly more downloads for nine open access articles than for the 121 non-open access articles (Wilcoxon rank correlation, *P* < .01). - The number of citations for open access papers did not differ significantly from that of non-open access papers (Wilcoxon rank-sum test *P* = .98). When we stratified by open access status, we found no effect of the SM exposure on downloads and citations. | - Funding: No funding received - Declared COI: All authors declare no conflict of interest |

### Studies assessing the correlation between social media metrics and traditional bibliometrics (n=44)

| **Author, year [reference number] Type of study** | **Report characteristics** | **Metrics** | **Methodological quality indicators** | **Results** | **Funding**  **Conflicts of interest and Notes** |
| --- | --- | --- | --- | --- | --- |
| Amath, 2017  Quantitative (cross-sectional)  Abstract and FT | - Health Area: Medical Education - Journal: Medical Education in Review - Population/Unit of analysis: all articles published on Medical Education journal (n=482) in the selected timeframe - Sources of data: Scopus, Altmetric Bookmarklet tool, journal - Period of analysis: 2012-2013 (search conducted on September 2015) | - Type of social media: Twitter, Mendeley - Social media metrics: Altmetric Attention Score - Bibliometrics: traditional citations obtained from Scopus | - Time adjustment: No - Confounders: No - Scatterplot: Yes - Type of test: Pearson r | - The correlation between Tweet counts and Altmetrics score was very strong both without (r = .96) and with (r = .98) the inclusion of the outlying data point in 2013 (Altmetric score: 104; Tweet counts: 220). - Citations strongly correlated with access counts and Mendeley downloads; weakly (for 2013 articles) to moderately (for 2012 articles) correlated with Twitter mentions, potential exposure to Twitter followers and Altmetric scores. | - Funding: Not funded - Declared COI: “None.” |
| Araujo, 2017  Qualitative (cross-sectional) | - Health Area: Parkinson’s disease - Journal: Journal of Parkinson's Disease - Population/Unit of analysis: Top 20 scientific articles with the highest Altmetric scores - Sources of data: PubMed (MeSH “Parkinson’s Disease”); Altmetric Explorer database v2; Scopus citations - Period of analysis: 2013-2016 (search conducted on 29/12/2016) | - Type of social media: Twitter, Facebook & news outlets - Social media metrics: Altmetric Attention Score - Bibliometrics: traditional citations obtained from Scopus | - Time adjustment: No - Confounders: No - Scatterplot: No - Type of test: Pearson r? (not specified) | - Mainstream media mentions and Twitter mentions comprised a majority of the attention that these articles received online - “Virtually all papers were published in journals with high impact factors, indicating a potential correlation between traditional metrics and Altmetrics; an exception is the paper in 8th position—dealing with cannabis (medical marijuana), as a possible treatment for motor and non-motor symptoms of Parkinson’s disease —with the highest number of raw mentions by far, mainly due to Twitter.” - The study published in the Journal with the highest Impact Factor ranked 16th based on Altmetric score. | - Funding: Not Reported - Declared COI: “The authors have no conflict of interest to report.” |
| Araujo, 2018  Quantitative (cross-sectional) | - Health Area: Physiotherapy - Journal: Journal of Medical Internet Research - Population/Unit of analysis: 200 randomly selected articles from Physiotherapy Evidence Database (PEDro) - Sources of data: PEDro; Altmetric scores; Web of Science - Period of analysis: 2013-2016 (search conducted on 29/12/2016) | - Type of social media: any - Social media metrics: Altmetric mentioned and reader scores - Bibliometrics: traditional citations obtained from Web of Science | - Time adjustment: Yes, time period of included studies was 1 year before the search - Confounders: Impact factor, paper published as open access (yes/no), total PEDro score, number of years since publication, normalized citation count, and type of title - Scatterplot: No - Type of test: Univariate (linear) regression | - Being published in an open access journal was not independently associated with *Altmetric mentioned* score - Altmetric mentioned score associated with: impact factor (β-coefficient=3.4 points), number of years since publication (β coefficient=–4.9), number of citations divided by years since publication (β-coefficient=5.2 points), and descriptive title (β-coefficient=–29.4 points) - *Altmetric reader* associated with number of citations divided by years since publication (β-coefficient=10.1 points, 95% CI 7.74-12.46). | - Funding: Coordenação de Aperfeiçoamento de Pessoal de Nível Superior (CAPES) for funding ACA’s PhD scholarship, and Australia’s National Health and Medical Research Council for funding CGM’s fellowship. - Declared COI: “None declared.” |
| Azer, 2019  Quantitative (cross-sectional) | - Health Area: Medical professionalism - Journal: BMJ Open - Population/Unit of analysis: 50 most-cited articles in medical professionalism identified by searching the Web of Science database - Sources of data: Web of Science; Altmetric scores; - Period of analysis: 1994-2011 | - Type of social media: any - Social media metrics: Altmetric score - Bibliometrics: traditional citations obtained from Web of Science | - Time adjustment: No - Confounders: Gender of author, grouping the identified top-cited articles into categories - Scatterplot: No - Type of test: Pearson r | - Twitter (54%) and Mendeley (62%) were the most popular Altmetric resources. - No correlation was found between the number of citations and the Altmetric scores (P = .66). | - Funding: College of Medicine Research Center, Deanship of Scientific Research, King Saud University, Riyadh, Saudi Arabia. - Declared COI: “None declared” |
| Baan, 2017  Quantitative (cross-sectional)  Message from the editors | - Health Area: Transplantation - Journal: Transplantation - Population/Unit of analysis: Articles published in 2015 (volume 99) - Sources of data: Journal transplantation - Period of analysis: 2015 | - Type of social media: Twitter - Social media metrics: Twitter mentions - Webometrics: Number of views and online downloads | - Time adjustment: No - Confounders: No - Scatterplot: No - Type of test: Mann-Whitney test - citations comparing tweeted vs. non-tweeted articles | - Articles published in 2015 were downloaded three times more often when they were tweeted (P < .01) compared to non-tweeted articles - Seventeen (34%) of the 50 most downloaded and viewed articles in 2015 (volume 99) were tweeted | - Funding: Not funded - Declared COI: None reported |
| Batooli, 2016  Quantitative (cross-sectional) | - Health Area: Medical sciences - Journal: Electronic Physician - Population/Unit of analysis: All articles (n= 533) published by scholars at Kashan University of Medical Sciences (Iran) - Sources of data: Scopus Citation Index; ResearchGate; Mendeley - Period of analysis: 1997-2014 (search conducted in March 2014) | - Type of social media: ResearchGate, Mendeley - Social media metrics: number of views in Research gate and Mendeley - Bibliometrics: Scopus citation index | - Time adjustment: No - Confounders: No - Scatterplot: No - Type of test: Spearman rho | - The significant relationship between the number of views of the articles in ResearchGate and Mendeley and citations in Scopus with 99% confidence level and an error level less than 1%. - A positive correlation between the number of views of articles in ResearchGate with citation number of the articles in Scopus (r = .31) - A positive correlation between reading frequency of the articles in Mendeley with the citation number of the articles in Scopus (r = .25) - Correlation between the numbers of views of articles in ResearchGate was associated with higher citations of reading frequency of the articles in Mendeley with the number of citations to the articles. | - Funding: Vice Chancellor for Research at the Kashan University of Medical Sciences. - Declared COI: “There is no conflict of interest to be declared.” |
| Calopedos, 2017  Quantitative (cross-sectional) | - Health Area: Urology - Journal: BJU International - Population/Unit of analysis: English-language urology articles (N=22) - Sources of data: PubMed (keyword search of urology and social media); Google Scholar citations - Period of analysis: 2010-2015 (search conducted on 15/3/2016) | - Type of social media: Any - Social media metrics: Altmetric score - Bibliometrics: Citations (Google Scholar) | - Time adjustment: Yes, two year window - Confounders: No - Scatterplot: Yes - Type of test: Pearson r | - Significant positive correlation between Altmetric and citations; Pearson correlation coefficient (r) of .72 (*P* < .01) | - Funding: Not reported - Declared COI: “none” |
| Chang 2019  Quantitative (cross-sectional) | - Health Area: Pediatric Surgery - Journal: Journal of Surgical Research - Population/Unit of analysis: 140 articles appearing on 14 core journals on pediatric surgery (Journal of Pediatric Surgery, Pediatrics, Annals of Surgery, New England Journal of Medicine, Pediatric Surgery International, Journal of Pediatrics, Journal of Trauma, Journal of Clinical Oncology, Journal of Biological Chemistry, Proceedings of the National Academy of Sciences (PNAS), Cancer, American Journal of Obstetrics and Gynecology, Archives of Surgery, and Surgery) - Period of analysis: 2012-2015 - Sources of data: 14 core journals; Scopus; JCR for impact factor | - Type of social media: Any - Social media metrics: Altmetric score - Bibliometrics: Citations from Scopus, impact factor from JCR | - Time adjustment: Yes, age of twitter account - Confounders: No - Scatterplot: Yes - Type of test: Pearson r | - In 2012, citations correlated strongly with journal IF (r = .82, *P* < .01). Altmetric scores positively correlated with citations (r = .12, *P* = .01) but did not correlate with IF (r = .08, *P* = .16). - Altmetric scores in 2015 also showed weak positive correlation with citations (r = .24, *P* < 0.01) but additionally weakly correlated with IF (r = .20, *P* =.01). - Increasing age of a journal’s Twitter account resulted in increasing correlation between Altmetric score and citations in both 2012 (r = .30, *P* = .17) and 2015 (r = .51, *P* = .04). | - Funding: Not Reported - Declared COI: “No proprietary or commercial interest in any product mentioned or concept discussed in the article” |
| Chen 2019  Quantitative (cross-sectional)  Abstract | - Health Area: Rheumatology - Journal: Rheumatology - Population/Unit of analysis: 1,460 articles appearing on Rheumatology journal - Period of analysis: 2010-2015 - Sources of data: Rheumatology journal | - Type of social media: Any - Social media metrics: Altmetrics - Bibliometrics: Citations and downloads | - Time adjustment: No Confounders: Yes, topic of disease area - Scatterplot: No - Type of test: Linear regression | - No correlations between diseases topic and downloads (R^2^ = .02, *P* = .03), citations (R^2^ = .01, *P* = .29) or Altmetric (R^2^ = .03, *P* = .02). - Positive association between number of citations and downloads (R^2^ = 0.29, *P* < .01). - Correlations between Altmetric and downloads (R^2^ .03, *P* < .01) or citations (R^2^ = .004, *P =* .45). | - Funding: Not Reported - Declared COI: Not Reported |
| Chiang, 2016  Quantitative (cross-sectional)  Abstract | - Health Area: Gastroenterology - Journal: Proceedings of the Conference: Digestive Disease Week 2016, DDW 2016 - Population/Unit of analysis: 1,671 articles appearing on five journals (Gastroenterology, Clinical Gastroenterology, and Hepatology, Gastrointestinal Endoscopy, Pancreas, American Journal of Gastroenterology); 482 being tweeted by the official journal account - Period of analysis: 1/1/2012-31/12/2012 - Sources of data: 5 gastroenterology journals; Google Scholar; | - Type of social media: Twitter - Social media metrics: Twitter mentions - Bibliometrics: Citations (Google Scholar) | - Time adjustment: No Confounders: Yes, categorized into 17 sub-topics based on subspecialty focus - Scatterplot: No - Type of test: Multivariate analysis with linear regression | - Significant overall association between article type and citations was noted on ANOVA (*P* < .01), with guidelines/technical reviews (90.23 +/- 355.57), meta-analyses/systemic reviews (75.22 +/- 124.85), and prospective studies (42.96 +/- 54.26) having the most citations. - A significant association between article type and the number of re-tweets on ANOVA (*P* < .01), with guidelines/technical reviews (1.04, CI 0.22-1.87, *P* < .05) and meta-analyses/ systemic reviews (1.04, CI 0.22-1.86, *P* < .05) being re-tweeted significantly more than basic science articles on pair-wise comparisons. - Media additions to tweets were not associated with number of citations or re-tweets. | - Funding: Not Reported - Declared COI: Not Reported |
| Cho, 2017  Quantitative (cross-sectional) | - Health Area: Medical sciences (among others: engineering, social science, humanities) - Journal: Performance Measurement and Metrics - Population/Unit of analysis: All publications from South Korean studies (N= 383; 98 in medical sciences) - Source of data: Scopus - Period of analysis: 2010-2014 | - Type of social media: ImpactStory (including Blogs, Twitter, Facebook, Google +, Mendeley) - Social media metrics: Altmetrics - Bibliometrics: Citations (Scopus) | - Time adjustment: No Confounders: Yes data were divided into four categories including “discussed”, “saved”, “viewed” and “mentioned” - Scatterplot: No - Type of test: Pearson r | - A statistically significant and positive correlation (r = .40-.70) between "saved" and "cited" in every academic field. Therefore, the more the papers cited in the journal, the more papers saved on Mendeley, and used in the diverse stage of experts' activities. - In medical science, there is a positive correlation r = .53 between “discussed” and “saved” unlike in the other fields. - “Discussed” means the extent to which the research results were exposed to social media such as Twitter and blogs and explains the extensive social impact of the research. The category of “saved” means the number of papers saved in a reference management tool such as Mendeley | - Funding: "this research received no specific grant from any funding agency in public, commercial or not-for-profit sectors - Declared COI: Not Reported |
| Costas, 2015  Quantitative (cross-sectional) | - Health Area: Biomedical and health sciences (among others: life and earth sciences, mathematics and computer science, natural sciences and engineering, social sciences, and humanities) - Journal: Journal of the Association for Information Science and Technology - Population/Unit of analysis: 718,315 records with a digital object identifier (DOI) of which analyzed 500,229, 217,115 in health sciences - Sources of data: Altmetrics, Web of Science - Period of analysis: 2011-2013 (search conducted on 14/10/2013) | - Type of social media: Facebook, Twitter, blogs, google+, news outlet (excluded Mendeley) - Social media metrics: Altmetrics - Bibliometrics: Journal Citation Score (JCS), total citation score, a journal to field impact score | - Time adjustment: Yes, Filtering of Publications for the Study between 2011-2012 - Confounders: Yes, by major fields - Scatterplot: Yes - Type of test: PCA; Spearman rho | - A clear, positive relationship between the number of Altmetrics and the average citation impact and JCS of the publications, in a way that publications with more Altmetrics also tend to have more citations. - The source that provides more Altmetric scores is Twitter, with 13% of all the publications that get some Twitter mentions, followed at an important distance by Facebook (2.5%), mentions in blogs (1.9%), Google+ accounts mentions (0.6%), and finally mentions in news outlets (0.5%) | - Funding: This work is partially supported by the EU FP7 ACUMEN project - Declared COI: Not reported |
| Dal-Ré, 2017  Quantitative (cross-sectional) | - Health Area: Medical sciences - Journal: Revista Clínica Espanola - Population/Unit of analysis: 410 articles: 228 original investigations (i.e., research articles and systematic reviews/meta-analysis) and 182 opinion articles (e.g., editorials, commentaries, viewpoints) published in the 2015 first 4 printed issues of 4 top-ranked general medicine journals (Lancet, JAMA, BMJ and Annals of Internal Medicine) and 1 top-ranked journal on five different medical specialties that provide Altmetric Attention Score of published articles (Lancet Infectious Diseases, JAMA Psychiatry, Gastroenterology, JAMA Pediatrics and Journal of Clinical Oncology) - Sources of data: Altmetrics, Google Scholar - Period of analysis: 1/2015-11/2016 | - Type of social media: Twitter - Social media metrics: Altmetrics - Bibliometrics: Google Scholar citations | - Time adjustment: Yes, articles got between 19 and 27 months to accrue citations - Confounders: Yes, different journals - Scatterplot: No - Type of test: Spearman rho | - Very strong correlations (*r* ≥ .80) were found between the occurrence of, at least, one tweet and Altmetric Attention Score and with Altmetric Attention Score belonging to both original investigations and opinion articles and with articles published in general medical journals. - Altmetric Attention Score and Twitter mentions were strongly/moderately and moderately/weakly associated with citation counts, respectively, although it widely varies depending on the journal considered. | - Funding: Not reported - Declared COI: Not reported |
| Dardas, 2019  Quantitative (cross-sectional) | - Health Area: Nursing - Journal: Advanced Nursing - Population/Unit of analysis: top 100 articles with high Altmetric attention score from nursing journals identified through JCR and WoS - Sources of data: Altmetrics, WoS, Scopus - Period of analysis: 2012-2018 | - Type of social media: Any - Social media metrics: Altmetrics - Bibliometrics: Google Scholar citations and WoS | - Time adjustment: No - Confounders: Yes, different article categories - Scatterplot: Yes - Type of test: Pearson r | - Twitter and Mendeley are the most frequently used social media tools - Significant positive and moderate relationship between articles’ AASs and their citation counts on Scopus (*r* = .34, *P <* .01) and Web of Science (*r* = .37, *P <* .01). - after excluding articles aged less than 2 years since its publication. The results still showed significant correlations between AASs and number of citations on Scopus (*r* = .44, *P <* .01) and Web of Science (*r* = 0.44, *P <* .01). - significant negative and strong relationship between publication date and citation counts on Scopus (*r* = −0.81, *P <* .01) and Web of Science (*r* = −.78, *P <* .01) and the scatterplots suggested that these are linear correlations. However, there was no significant relationship between publication date and AASs (*r* = −.14, *P =* .16). | - Funding: “This research received no specific grant from any funding agency in the public, commercial, or not-for-profit sectors” - Declared COI: “No conflict of interest has been declared by the authors.” |
| Delli, 2017  Quantitative (cross-sectional) | - Health Area: Dental Medicine - Journal: Oral Diseases - Population/Unit of analysis: Top 100 dental articles published on 91 dental journals in 2015 JCR - Sources of data: Altmetric Explorer, JCR, Scopus - Period of analysis: 2015 | - Type of social media: Any - Social media metrics: Altmetrics - Bibliometrics: Citation data from Scopus | - Time adjustment: Yes, time since publication - Confounders: Yes, different groups of articles - Scatterplot: Yes - Type of test: Spearman rho | - No correlation was detected between AAS and number of citations. - After excluding articles that had been recently published and, thus, possibly not given the chance to be cited yet (time since publication ≤ 2 years), showed no significant correlation between AAS and number of citations (*r* = .27, *P* > .05). | - Funding: Not reported - Declared COI: Not reported |
| Eysenbach, 2011  Quantitative (cross-sectional) | - Health area: not specified (JCR puts the journal in medical informatics, clinical medicine) - Journal: Journal of Medical Internet Research) - Population/Unit of analysis: 4,208 tweets including links to 286 JMIR articles - Sources of data: Twitter; Scopus; Google Scholar - Period of analysis: 7/2008-11/2010 | - Type of social media: Twitter - Social media metrics: tweetations (mentioning the article in a tweet) - Bibliometrics: Citation data from Scopus and Google Scholar | - Time adjustment: Yes Confounders: Yes - Scatterplot: Yes - Type of test: Pearson r, Spearman rho, log-linear correlation test | - The Pearson correlations between tweetations and citations were moderate and statistically significant, with correlation coefficients ranging from .42 to .72 for the log-transformed Google Scholar citations, but were less clear for Scopus citations and rank correlations - Highly tweeted articles were 11 times more likely to be highly cited than less-tweeted articles (9/12 or 75% of the highly tweeted article were highly cited, while only 3/43 or 7% of less-tweeted articles were highly cited; rate ratio 0.75/0.07 = 10.75, 95% confidence interval, 3.4–33.6. | - Funding: Crowdfunding campaign - Declared COI: "the author is editor and publisher of JMIR and is a shareholder of JMIR Publications Inc., which owns and publishes JMIR.” |
| Hassona, 2019  Quantitative (cross-sectional) | - Health Area: Dental Medicine - Journal: Oral Diseases - Population/Unit of analysis: Top 100 oral cancer articles available from Altmetric Explorer - Sources of data: Altmetric Explorer, JCR, Scopus - Period of analysis: 2018 | - Type of social media: Any - Social media metrics: Altmetrics - Bibliometrics: Citation data from Scopus and Google Scholar | - Time adjustment: No - Confounders: Yes, article type, topic domain, and article access (i.e., open access vs. pay for access) - Scatterplot: No - Type of test: Spearman rho | - Weak positive correlation between AAS and time since publication (*r* = .29, *P* > .05). - No significant correlation was detected between AAS and number of citations in Scopus or in Google Scholar. Given that some studies were recently published and might not have the chance to be cited - After excluding articles aged <2 years since publication. The results still showed no significant correlation between AAS and number of citations in Scopus (*r* = .19, *P* > .05) or Google Scholar (*r* = .23, *P* > .05). | - Funding: Not reported - Declared COI: “Authors declare no conflict of interest” |
| Haustein, 2014  Quantitative (cross-sectional) | - Health area: Biomedical and health sciences - Journal: Journal of the Association for Information Science and Technology - Population/Unit of analysis: 1,431,576 articles available from PubMed and WoS - Sources of data: PubMed Web of Science; Altmetric - Period of analysis: 2010-2012 | - Type of social media: All except Mendeley - Social media metrics: Altmetric data - Bibliometrics: Citations from WoS | - Time adjustment: Yes, year of publication - Confounders: Yes, stratified by type of journals - Scatterplot: Yes - Type of test: Spearman rho | - Spearman correlations between mean Twitter citation rate for articles tweeted at least once (*T/Ptweeted*) in 2011: positive correlation with impact factor (rho = .24, *P* < .01), eigenfactor (rho = .24, *P* < .01), article influence score (rho =.28, *P* < .01), and immediacy index (rho = .25, *P* < .01). | - Funding: “This research was part of the international Digging into Data program (funded by AHRC/ESRC/JISC (UK), SSHRC (Canada), and the National Science Foundation (US; grant #1208804); funding from the Canada Research Chair program.” - Declared COI: Not reported |
| Haustein, 2015  Quantitative (cross-sectional) | - Health area: Biomedical and health sciences - Journal: PLOS ONE - Population/Unit of analysis: 1,339,297 articles (of which 595,254 in biomedical and health sciences) - Sources of data: Web of Science; Altmetric - Period of analysis: 2012 | - Type of social media: All except Mendeley - Social media metrics: Altmetric data - Bibliometrics: Citations in Thomson Reuters’ Web of Science | - Time adjustment: No - Confounders: Yes, stratified by document characteristics - Scatterplot: Yes - Type of test: Spearman rho | - In the biomedical and health sciences field, no correlation was observed between citations and social media indices. | - Funding: Supported by the Alfred P. Sloan Foundation Grant - Declared COI: "No conflict of interest." |
| Hayon, 2019  Quantitative (cross-sectional)  Abstract and FT | - Health area: Urology - Journal: Urology. - Population/Unit of analysis: 213 articles from 7 prominent urology journals - Sources of data: Scopus; Google Scholar; Altmetric - Period of analysis: 12/ 2014- 1/2015 | - Type of social media: Twitter - Social media metrics: Twitter mentions - Bibliometrics: Scopus, Google Scholar citations | - Time adjustment: Yes, 37 months after print publication. - Confounders: No - Scatterplot: NoType of test: One-way ANOVA analysis was used to compare the presence or absence of Twitter mentions and the number Scopus and Google Scholar citation | - A positive correlation between the number of Twitter mentions and the number of Scopus citations (r = .33, *P* < .01) and Google Scholar (R = .35, *P* < .01) - Self-tweeting associated with an increased number of citations in google scholar (*P* = .02) and Scopus scores (*P* < .01) - Articles with twitter mentions were found to have two-fold more Scopus citations (*P* < .01), 2.1 fold more Google Scholar citations (*P* < .01). | - Funding: not reported - Declared COI: Not reported |
| Heydarpour, 2017  Quantitative (cross-sectional)  Abstract | - Health Area: Multiple Sclerosis research - Journal: Multiple Sclerosis Journal - Population/Unit of analysis: 5,251 articles, of which 4,693 with scholarly mentions - Sources of data: Altmetric Explorer and PubMed, WoS, SCImago Journal Rank, - Period of analysis: 2016 | - Type of social media: Any - Social media metrics: Altmetrics - Bibliometrics: Citations from WoS | - Time adjustment: No Confounders: No - Scatterplot: No - Type of test: Pearson r; linear regression | - Refined top 5 percent articles to be specific to MS research, number of news stories for these articles was not correlated with any scientific metric (*P* > .05 for IF, 5yr-IF, Citations, SNIP, SJR). - Number of blog posts was weakly correlated with IF (r = .22, *P* < .01), 5yr-IF (r = .23, *P* < .01), Citations (r = .32, *P* < .01), SNIP (r = .27, *P* < .01), and SJR (r = .16, *P* = .02). - Number of tweets was weakly correlated with IF (r = .35, *P* < .01), 5yr-IF (r = .34, *P* < .01), Citations (r = .24, *P* < .01), SNIP (r = .35, *P* < .01), and SJR (r = .28, *P* < .01). - The Altmetric attention score was very weakly correlated with IF (r = .14, *P* = .04), Citations (r = .16, *P* = .02), and SNIP (r = .17, *P* = .01). | - Funding: Not reported - Declared COI: “two authors declare: Nothing to disclose; Mohammad Ali Sahraian declares no conflict of interest regarding this study, Dr. Sahraian has received educational, research grants, lecture honorarium, travel supports to attend scientific meetings from Biogen-Idec, Merck-Serono, Bayer-Schering, Novartis, Cinnagen, Osveh, Zistdaru, Zahravi and Genzyme.” |
| Jabaley, 2018  Quantitative (cross-sectional) | - Health Area: Sepsis research - Journal: Critical Care - Population/Unit of analysis: top 50 sepsis publications available from PubMed search - Sources of data: PubMed, Altmetric - Period of analysis: 2012-2017 | - Type of social media: Any - Social media metrics: Altmetrics - Bibliometrics: Citations from WoS and Scopus | - Time adjustment: Yes, used a 60-day window for Altmetric data - Confounders: Yes, stratified by publication with highest number of citations - Scatterplot: Yes - Type of test: Pearson r? (not specified) | - 46 articles with highest citation counts, both the overall Altmetric attention score and Twitter user activity were both poorly correlated with the Web of Science citation count (expressed as citations per year) as depicted with linear modeling and two-dimensional density estimates - Publications concerning the mechanisms of sepsis and systemic inflammation had lower overall Altmetrics performance, and correlations between these and conventional citation counts were lower than those for clinically oriented publications. - Factoring journal IF against conventional citations per year modestly improved the strength of this correlation for mechanistic publications but worsened the correlation for clinical publications | - Funding:” Internal departmental funds were used to support the authors' time to conduct the current study.” - Declared COI: “The authors declare that they have no competing interests. Altmetric.com granted Dr. Jabaley unrestricted access to their database via an application programming interface in response to an investigator-initiated request but provided no conceptual or material support. Dr. Blum reported an equity stake in Intensix (Netanya, Israel).” |
| Jadhav 2019  Quantitative (cross-sectional) | - Health Area: Neurointerventional Surgery - Journal: Journal of Neurointerventional Surgery - Population/Unit of analysis: 451 articles published online first in JNIS - Sources of data: journal database, WoS - Period of analysis: 2/2015-10/2016 | - Type of social media: Twitter - Social media metrics: number of followers, clicks - Bibliometrics: Citations from WoS | - Time adjustment: No Confounders: No - Scatterplot: Yes - Type of test: Pearson r? (not specified) | - The strongest predictors of citations at 2 years were the number of clicks, (with a 1% increase in citations for every additional click) and the level of evidence presented by the article, with a 20% increase in citations for every one unit increase in higher level of evidence - Articles focusing on socio- economics are associated with the highest number of citations followed by stroke and aneurysm. - Twitter impressions and engagement did not predict conventional citations; - The level of evidence of the publication and the topic of research strongly predicts future citations. The number of clicks also appears to be a strong predictor of future citations and the number of clicks increases as the number of Twitter users also grows. | - Funding: “The authors have not declared a specific grant for this research from any funding agency in the public, commercial or not-for-profit sectors.” - Declared COI: “None declared” |
| Jeong, 2019  Quantitative (cross-sectional) | - Health Area: Coloproctology - Journal: Colorectal Diseases - Population/Unit of analysis: - 404 articles published on three journals with Twitter profile (Diseases of the Colon & Rectum, Colorectal Disease and Techniques in Coloproctology) - Sources of data: JCR - Period of analysis: 6/2015-5/2016 | - Type of social media: Twitter - Social media metrics: Twitter analytics - Bibliometrics: Citations from Google Scholar | - Time adjustment: No - Confounders: Yes, articles categorized into predetermined groups and using Twitter vs. no twitter - Scatterplot: No - Type of test: Student’s t-test and ANOVA | - Citation rates of articles that featured on Twitter were significantly higher than those that did not (11.4 ± 9.2 vs 4.1 ± 3.1, *P* < .01). - In multivariate analysis, Twitter exposure (OR 8.6, *P* < .01), European Union nationality (OR 2.4, *P* < .01), Colorectal Disease journal (OR 3.3, *P* = .01) and systematic review articles (OR 3.4, *P* = .01) were associated with higher citation levels. | - Funding: Not reported - Declared COI: “None” |
| Knight, 2014  Quantitative (cross-sectional)  Abstract and FT | - Health area: Solid-organ transplantation - Journal: Transplantation - Population/Unit of analysis: 6,979 articles with citation data available; 1,346 with social media mention - Sources of data: Medline (MeSH related to solid organ transplantation); Scopus; Altmetric - Period of analysis: 1/8/2011-31/8/2012 | - Type of social media: Twitter, Facebook, Blogs, Mendeley, CiteULike, Pinterest - Social media metrics: Altmetrics - Bibliometrics: Scopus citations | - Time adjustment: Yes, time to last mention on social media - Confounders: Yes, moderating variables included: publication language, article type (as recorded in the Medline record) and article content or topic - Scatterplot: Yes - Type of test: Spearman rho; binary logistic regression (odd ratios) between publication type and having at least one social media mention, one social bookmark | - Correlation between social media mentions and citation numbers was significant but poorly predictive (Spearman’s r = .16; *P* < .01) - The median numbers of citations were significantly higher if an article had a social media mention (*P* < .01). - Odds of an article being highly cited were significantly higher when mentioned on social media (odds ratio [OR], 2.58; *P* < .01) - Highest citation rates were seen when an article was mentioned on a blog OR for more than ten citations (*P* < .01) - Correlation between the number of social bookmarks for an article and the number of citations is significant but poorly predictive (Spearman's r = .23, *P* < .01) - Articles that are bookmarked have significantly higher mean citation counts (*P* < .01) - Odds of an article being highly cited are significantly higher if an article has social bookmark activity (*P* < .01). - Bookmarking on CiteUlike was associated with higher median citation rates than Mendeley | - Funding: Not reported - Declared COI: Not reported |
| Konstantiniuk, 2015  Quantitative (cross-sectional)  Abstract | - Health Area: Sepsis Research - Journal: Wiener Klinische Wochenschrift - Population/Unit of analysis: 12 articles on sepsis compared to 8 ICU articles - Sources of data: Twitter, Altmetrics, ResearchGate, Google Scholar and Web of Science. - Period of analysis: Not indicated | - Type of social media: Twitter, ResearchGate - Social media metrics: Altmetrics - Bibliometrics: Citations from Google Scholar and WoS | - Time adjustment: No - Confounders: Yes, article type, topic domain - Scatterplot: No - Type of test: Pearson r? (not specified) | - The Altmetric Score neither correlated with Google Citations (*r* = - .001, *P =* 1.0) nor publishing date (*r* = .02, *P =* .95). | - Funding: Not reported - Declared COI: Not reported |
| Liu, 2013  Quantitative (cross-sectional) | - Health area: not specified (PLOS is a PLOS is a nonprofit open-access science, technology and medicine publisher) - Journal: Journal of Medical Internet Research - Population/Unit of analysis: 33,128 academic articles - Source of data: Article level metrics dataset from PLOS API - Period of analysis: up to 14/12/2011 | - Type of social media: blogs (Nature, researchblogging.org), social bookmarking (Bloglines, Citeulike, Connotea) - Social media metrics: Altmetrics, Blog postings, trackbacks, Social Bookmarking - Bibliometrics: HTML views, PDF downloads, citations (Scopus, PubMed, CrossRef) | - Time adjustment: No - Confounders: No - Scatterplot: Yes - Type of test: Spearman rho | - Of the 42 correlational measures between Altmetrics and bibliometrics, 37 (88%) were significant. - The median calculated correlation of statistically significant measures was .07 with an interquartile range of 0.10 (.06-.16) | - Funding: Not reported - Declared COI: "None declared." |
| Livas, 2018  Quantitative (cross-sectional) | - Health Area: Orthodontics Medicine - Journal: European Journal of Orthodontics - Population/Unit of analysis: top 200 articles available from Altmetrics Explorer - Sources of data: Altmetric Explorer and JCR - Period of analysis: 27/1/2017-27/4/2017 | - Type of social media: Any - Social media metrics: Altmetrics - Bibliometrics: Citations from Scopus | - Time adjustment: Yes, time since publication - Confounders: Yes, Altmetric attention score plotted against: (i) article title; (ii) journal title; (iii) time interval since publication, i.e. up to 1 year, >1 and ≤2 years, >2 and ≤5 years, >5 and ≤10 years, more than 10 years; (iv) number of authors and affiliations; (v) type of the affiliation of the corresponding author, (vi) origin of the article; (viii) study type; (ix) full text availability; (x) funding, - Scatterplot: Yes - Type of test: Spearman rho | - No correlation was observed between AAS and citations in Scopus (*r* = .09, *P =* .42). | - Funding: Not reported - Declared COI: “None declared.” |
| Maggio, 2018  Quantitative (cross-sectional) | - Health Area: Health professions education - Journal: Perspectives in Medical Education - Unit of analysis: - Sources of data: 2,486 articles with Altmetrics published in health profession education - Sources of data: WoS and Altmetric Explorer - Period of analysis: 2013-2014 | - Type of social media: Any - Social media metrics: Altmetrics - Bibliometrics: Citations from WoS | - Time adjustment: Yes, time in weeks included in the model - Confounders: Yes, by IF, publicly accessible articles, different types of outlets - Scatterplot: Yes - Type of test: negative binomial and linear regression models | - Blogging was associated with the greatest increase in citations (13% increase), whereas Tweets (1.2%) and Mendeley (1%) were associated with smaller increases. - Blogging activity (IRR = 1.13, 95% IRR confidence interval [CI]=1.01, 1.25) and JIF (IRR = 1.21, 95% IRR CI = 1.13, 1.30) had the largest, positive effect on citations. - Tweets and Mendeley saves were both statistically significantly related to citations, although the effect sizes were small (Tweets (IRR = 1.01, 95% IRR CI = 1.00, 1.01), Mendeley (IRR = 1.01, 95% IRR CI = 1.00, 1.01)). | - Funding: Not reported - Declared COI: Not reported |
| Matava, 2017  Quantitative (cross-sectional)  Abstract | - Health Area: Pediatric anesthesiology - Journal: Anesthesia & Analgesia - Unit of analysis: top 100 articles with highest Altmetric attention score - Sources of data: Altmetric Explorer and Scopus - Period of analysis: 5/2016 | - Type of social media: Twitter, Mendeley - Social media metrics: Altmetrics - Bibliometrics: Citations from Scopus | - Time adjustment: No Confounders: No - Scatterplot: No - Type of test: Pearson r? (not specified) | - Altmetric scores were highly correlated with Twitter mentions (r = .81) but were not highly correlated with other social media metrics, citations, or access counts. - Strong association between mention on Mendeley with citation (r = 0.71; *P* = 0.02). - Twitter mentions were not associated with citations. | - Funding: Not reported - Declared COI: Not reported |
| Nolte, 2019  Quantitative (cross-sectional)  Abstract and FT | - Health Area: Urology - Journal: European Urology Focus - Population/Unit of analysis: 415 most promoted tweets with the #aua15 hashtag (American Urological Association 2015 meeting) related to 44 unique research articles - Sources of data: Twitter; Pubmed; JCR - Period of analysis: 1/5/2015-1/6/2015 | - Type of social media: Twitter - Social media metrics: Social media reception (Likes/Retweets), Klout score - Bibliometrics: Impact Factor | - Time adjustment: No - Confounders: Yes, subgroup analysis across strata of likes/RTs to assess the relationship with publication status - Scatterplot: Yes - Type of test: Pearson r | - There was a modest, positive correlation between the number of likes/RTs received and subsequent journal IF (r = .36). Across subgroups, posts with more likes/RTs were published more frequently (*P* < .01) and in journals with higher IF (*P* < .01). | - Funding: not reported - Declared COI: Not reported |
| O’Connor, 2017  Quantitative (cross-sectional)  Abstract and FT | - Health area: Urology - Journal: BJU International - Population/Unit of analysis: Top 5 cited articles for the top 10 ranking urology journals and top 50 highest-ranking articles as ranked by Altmetric score for 2014 and 2015. - Sources of data: Journal Citation Reports (JCR) (category “Urology and Nephrology”)/Scopus; Altmetric - Period of analysis: 2014-2015 (search conducted on 19/8/2016) | - Type of social media: not specified - Social media metrics: Altmetric - Bibliometrics: Citations (Scopus) | - Time adjustment: No - Confounders: Yes, media impact vs scientific impact group analyses - Scatterplot: Yes - Type of test: Spearman rho | - In the top 10 highest cite articles, there was a weak positive correlation between number of citations per article and Altmetric score (s = .35, 95% CI .16–.52, *P* < .01) and between Altmetric score and journal impact factor (r = .41, 95% CI .22–.56, *P* < .01) for the top 5 cited articles. - Among the top-scoring urology articles by Altmetric score, a weak negative correlation between Altmetric score and the number of citations per article (rs = .20, P = .05) and between Altmetric score and journal impact factor per article (rs = .12, P = .22). | - Funding: not reported - Declared COI: "none." |
| Punia, 2019  Quantitative (cross-sectional)  Letter | - Health Area: Neurological Research - Journal: JAMA Neurology - Unit of analysis: Top 100 articles from top 5 neurology journals out of 1050 articles - Sources of data: Altmetric Explorer and JCR - Period of analysis: 2016 | - Type of social media: Any - Social media metrics: Altmetrics Attention Score - Bibliometrics: Citations | - Time adjustment: No Confounders: No - Scatterplot: Yes - Type of test: Spearman rho | - There was a significant (*P* < .01), but weak correlation (*r* = .32) between the Altmetric Attention Score and the number of citations garnered by all 1050 research articles over 3 years | - Funding: Not reported - Declared COI: “None reported” |
| Quintana, 2016 [52]  Quantitative (cross-sectional)  Letter | - Health area: Psychiatry - Journal: American Journal of Psychiatry - Population/Unit of analysis: 438 journal print articles - Sources of data: American Journal of Psychiatry - Period of analysis: 2013-2014 (search conducted on 20/10/2015) | - Type of social media: Twitter - Social media metrics: Altmetric - Bibliometrics: Citations (Web of Science) | - Time adjustment: Yes - Confounders: No - Scatterplot: No - Type of test: Specific test: compares a given publication against the publications that chronologically appeared immediately before and after it. A “successful” test occurs when the number of Twitter mentions and citations for a given publication are both higher (or both lower) than the average of Twitter mentions and citations of the two adjacent articles | - Greater frequency of Twitter mentions is associated with more citations and with a higher rate of successful prediction compared with biomedical articles across all fields (57%). | - Funding: not reported - Declared COI: Not reported |
| Ramezani-Pakpour-Langeroudi, 2018  Quantitative (cross-sectional) | - Health Area: Clinical Medicine - Journal: Journal of Education and Health Promotion - Unit of analysis: 55 highly cited papers - Sources of data: Thomson Reuters Essential Science Indicator (ESI) - Period of analysis: 11/2015-12/2015 | - Type of social media: ResearchGate, Academia, LinkedIn, Mendeley - Social media metrics: Profile views, reads, citations RG score (ResearchGate); following, followers, documents, total views (Academia); readers (Mendeley); citations/mentions (LinkedIn) - Bibliometrics: Citations from Scopus | - Time adjustment: No Confounders: No - Scatterplot: No - Type of test: Pearson r | - A significant relationship was observed between the citation rates and RG indicators (*P* < .01). The relationship between profile views and the number of publications was not statistically signification, the relationship between reads, citations, and RG score was significant. In other words, reads, citations and RG score can be used to anticipate the citation rate (*P* < .01, *R^2^*= .76). - There was a significant relationship between the total citation and Academia indicators (*P* < .01). The relationship was significant for all 4 indicators (followers, following, total views, documents). No significant relationship was observed between the followers and following numbers and the total views with citation number, but the documents number can predict the citation rate (*P* < .01, *R*^2^= .17). | - Funding: “The present study is the partial result of the program ratified by Guilan University of Medical Sciences in Iran under the code IR.GUMS.REC.1395.184” - Declared COI: “There are no conflicts of interest” |
| Rosenkrantz, 2017  Quantitative (cross-sectional) | - Health area: Radiology - Journal: Academic Radiology - Population/Unit of analysis: 892 articles having a structured four-part abstract, indicative of an original scientific investigation - Sources of data: Journals of the Academic of Radiology, American Journal of Roentgenology, Journal of the American College of Radiology, and Radiology - Period of analysis: 2013 (June-July 2016) | - Type of social media: Any - Social media metrics: Altmetrics attention score - Bibliometrics: Citations from WoS | - Time adjustment: No - Confounders: Yes, imaging vs. non imaging articles - Scatterplot: Yes - Type of test: Nonparametric: Kruskal-Wallis; McNemar; Mann-Whitney | - The correlation was weak between the citation count and both the Altmetric Attention Score and the number of Twitter mentions (r = .17 – .20) - The correlations between the citation count and both the Altmetric Attention Score and the number of Twitter mentions were higher for articles with non-imaging rather than imaging content (r = .47–.62 vs. .13 – .19, respectively; *P* < 0.01). - In comparison, the citation count exhibited strong correlation with the number of Mendeley mentions (r = .57); this correlation was higher for articles with imaging rather than non-imaging content (r = .58 vs .42, respectively; *P* = 0.017). - Among the 92 articles within the top 10th percentile in terms of the Altmetric Attention Score, 25.0% were also in the top 10th percentile in terms of the citation count. | - Funding: Not reported - Declared COI: Not reported |
| Ruan, 2018  Quantitative (cross-sectional) | - Health Area: Plastic and Reconstructive Surgery - Journal: Plastic and Reconstructive Surgery - Unit of analysis: 55 most-cited articles published in Plastic and Reconstructive Surgery - Sources of data: Altmetric, Scopus - Period of analysis: 2014-2015 | - Type of social media: Mendeley, specifically - Social media metrics: Altmetrics - Bibliometrics: Citations from Scopus | - Time adjustment: No - Confounders: No - Scatterplot: No - Type of test: Pearson r | - No apparent correlation between Altmetric scores and Scopus citations (*P =* .58) or article subject themes (*P =* .63). - Citation was positively associated with download rates (*r* = .31, *P =* .02) and Mendeley reader number (*r* = .46, *P <* 0.01). - Mendeley reader number demonstrated high precision in identifying top-ranked citation articles (*P =* .04) despite its lack of direct association with Altmetric score (*P =* .83). | - Funding: “The authors did not receive any funding for this study. They have no financial disclosures” and report no conflicts of interest. - Declared COI: “The authors report no conflicts of interest.” |
| Ruano, 2018  Quantitative (cross-sectional) | - Health Area: Psoriasis research - Journal: PLOS One - Unit of analysis: 164 systematic reviews or meta analyses published up to July 4, 2016 - Sources of data: Medline, Embase, and Cochrane database; JCR - Period of analysis: 2016 | - Type of social media: Any - Social media metrics: Altmetrics - Bibliometrics: Citations from InCites JCR, Google Scholar | - Time adjustment: No - Confounders: Yes, subgroup analysis by methodological quality - Scatterplot: Yes - Type of test: PCA, factor analyses | - Regression analysis showed that social media activity and a journal’s impact factor had less influence than number of Mendeley and Scopus readers on the number of Google Scholar cites - Although a journal’s impact factor predicted the number of tweets (OR, 1.202; 95% CI, 1.087–1.049), the years of publication and the number of Mendeley readers predicted the number of citations in Google Scholar (OR, 1.033; 95% CI, 1.018–1.329) | - Funding: “This work has been funded in part by project ICI1400136 to JR, integrated into the National Plan of R+D+I 2008-2011 and co-financed by the ISCIII-Subdireccion General de Evaluacion nd European Regional Development Fund (ERDF), and by grant PP13/009 of Plan Propio de movilidad para investigadores del Instituto Maimonides de Investigacion Biome ́dica de Cordoba (IMIBIC). No funding was received from any pharmaceutical company. - Declared COI: “JR has received honoraria for lecturing and grants for research from Pfizer, honoraria for lecturing from Janssen-Cilag and Novartis, and other financial benefits from AbbVie and Novartis; FG-G has received honoraria for research from Pfizer, and for lecturing from AbbVie, Janssen-Cilag and Novartis; AVG-N has received honoraria for lecturing from Pfizer, Novartis, AbbVie, and Janssen-Cilag, and other financial benefits from AbbVie, Novartis, and Janssen-Cilag. MA-L, PA-M, JG-M, PJC-F, BM-L, JLS-C, JLH-R, MG-P, and BI-T have no disclosures.” |
| Scotti, 2017  Quantitative (cross-sectional) | - Health Area: Hospital/Oncology - Journal: International Journal of Biological Markers - Unit of analysis: 268 articles with Altmetric score out of 646 full-text articles published in 2013 in indexed journals (with a 2012 IF score) by researchers affiliated to the authors’ hospital - Sources of data: Altmetric, Scopus - Period of analysis: 2013 | - Type of social media: Any - Social media metrics: Altmetrics - Bibliometrics: Citations form Scopus | - Time adjustment: No - Confounders: Yes, subgroup analysis by institutional unit - Scatterplot: Yes - Type of test: Spearman rho | - The correlation between the sum of Altmetrics scores and the sum of IFs relative to all articles published in 2013 calculated for each of the departments of our institution was very high (Spearman’s rho = .88; *P* < .01). - The correlation between IF and single major components of Altmetrics such as Facebook (Spearman’s rho = .80; *P* < .01), Twitter (Spearman’s rho = .90, *P* < .01) and Mendeley (Spearman’s rho = .90, *P* < .01) was very good. | - Funding: Not reported - Declared Not: None reported |
| Shirazi, 2018  Quantitative (cross-sectional) | - Health Area: Health literacy - Journal: Payesh Health Monitor - Unit of analysis: 615 health literacy articles, published in year 2015, with digital object identifier (DOI), and indexed in Web of Science. - Sources of data: WoS - Period of analysis: 2015 | - Type of social media: Any - Social media metrics: Altmetrics - Bibliometrics: Citations from WoS | - Time adjustment: No Confounders: No - Scatterplot: No - Type of test: Spearman rho | - Mendeley and Twitter were the most used social media by health literacy scholars for sharing scientific outputs, ranked first and second by number of 492 and 487 articles respectively. - Statistically significant relationship between most Altmetrics and the number of citations in Web of Science. | - Funding: Not reported - Declared Not: None reported |
| Smith, 2019  Quantitative (cross-sectional | - Health Area: Gastrointestinal Endoscopy - Journal: Gastrointestinal Endoscopy - Unit of analysis: 2,361 original research articles published on the journal of which 2,050 cited at least once - Sources of data: Altmetric Explorer and Scopus - Period of analysis: 2010-2016 | - Type of social media: Any - Social media metrics: Altmetrics - Bibliometrics: Citations from Scopus | - Time adjustment: No Confounders: No - Scatterplot: No - Type of test: Pearson r; linear regression | - Tweetations was the strongest predictor of article citation (odds ratio, 14.16; 95% confidence interval, 8.9- 22.4; *P* < .01). - However, no significant association was observed between the number of tweeters and whether the article was cited. - Number of tweeters (b-coefficient = 2.3, P = .02), F1000 reviews (b-coefficient = 5.87, *P* < .01), policy documents (b-coefficient = 7.6, *P* < .01), and number of Mendeley readers (b-coefficient = 14.21, *P* < .01) were all significantly associated with citation rate of published articles. | - Funding: Not reported - Declared COI: Z. L. Smith, Editorial Review Board member and Social Media Board Member of Gastrointestinal Endoscopy; D. Bowman: Senior Managing Editor of Gastrointestinal Endoscopy and American Society for Gastrointestinal Endoscopy; M; B. Wallace: Editor-in-Chief of Gastrointestinal Endoscopy. A. L. Chiang: Social Media Board member at Gastrointestinal Endoscopy. All other authors disclosed no financial relationships relevant to this publication. |
| Thelwall, 2013  Quantitative (cross-sectional) | - Health area: not specified (review of PubMed records including medical sciences) - Journal: PLOS ONE - Population/Unit of analysis: 171-135,331 articles with non-zero altmetric.com score in at least one of the Altmetrics and with valid PubMed ID - Sources of data: PubMed; Altmetric; Web of Science - Period of analysis: Up to July 2011 (search completed on 1/1/2013) | - Type of social media: Twitter, Facebook, MSM, Reddits, blogs, Forums, Pinners, Linkedin, Research Highlights, Google + - Social media metrics: Altmetrics - Bibliometrics: Citations from WoS | - Time adjustment: Yes - Confounders: Yes, subgroup analysis by type of article - Scatterplot: No - Type of test: Specific test: compares a given publication against the publications that chronologically appeared immediately before and after it. A “successful” test occurs when the number of Twitter mentions and citations for a given publication are both higher (or both lower) than the average of Twitter mentions and citations of the two adjacent articles; Pearson r | - Correlations (Tweets -.19**, FbWalls .05**, RH .37**, Blogs 0.20**, Google+ .03**, MSM .09**, Reddits .06**, Forums .03**, Q&A .05**, Pinners .01**, LinkedIn .01**, ** *P* < .01) - The results provide strong evidence that six of the eleven Altmetrics (tweets, Facebook wall posts, research highlights, blog mentions, mainstream media mentions and forum posts) associated with citation counts, at least in medical and biological sciences and for articles with at least one Altmetric mention, but the methods used do not shed light on the magnitude of any correlation between the Altmetrics and citations. - The evidence also suggests that Google+ posts might have little or no association with citations, and too little data was available to be confident about whether four of the metrics (LinkedIn, pinners, questions, and Reddit's) associated with citation counts | - Funding: "funded by the Arts and Humanities Research Council/Economic and Social Research Council/Joint Information Systems Committee, Social Sciences and Humanities Research Council, and the National Science Foundation." - Declared COI: “No COI” |
| Thelwall, 2016  Quantitative (cross-sectional) | - Health area: Medical Sciences - Journal: Journal of the Association for Information Science and Technology - Population/Unit of analysis: 290,282 full-text articles in 45 subjects within Scopus Medicine - Sources of data: Scopus (Scopus Medicine category); Mendeley API via Webometric Analyst - Period of analysis: 2009 (search completed in August 2014; analytics derived on 11-15/11/2014) | - Type of social media: Mendeley - Social media metrics: Mendeley Readership - Bibliometrics: Citations from Scopus | - Time adjustment: Yes - Confounders: Yes, subgroup analysis by 45 subjects - Scatterplot: No - Type of test: Spearman rho | - The correlations between Mendeley readers and citations are significantly positive and strong (overall r = .69, *P* < .01in all cases) | - Funding: Not reported - Declared COI: Not reported |
| Wiehn, 2017  Quantitative (cross-sectional, trend)  Abstract | - Health area: Not specified - Journal: Proceedings of the Current Medical Research and Opinion. Conference: 13th Annual Meeting of the International Society for Medical Publication Professionals - Population/Unit of analysis: 36 articles - Source of data: Shire- sponsored articles; Altmetric - Period of analysis: 7/2016-12/2016 | - Type of social media: Twitter - Social media metrics: Altmetric score, ResearchGate reads, and journal-provided download information - Bibliometrics: Journal Impact Factor | - Time adjustment: No - Confounders: No - Scatterplot: No - Type of test: Pearson r? (not specified) | - No correlation was observed between the Altmetric score and journal impact factor (n=29 articles), the journal provided download (n=13), or Research Gate reads (n=36). - Articles published with open access had a higher mean Altmetric score (18.2, n=29) than those with restricted access (1.1, n=7). | - Funding: Not Reported - Declared COI: Not Reported |

1. The text reported in these columns is taken directly from the original source. [↑](#footnote-ref-1)
